# Progression of Metabolic Dysfunction–Associated Steatohepatitis in US Adults Using Linked Records and Claims

**DOI:** 10.1016/j.gastha.2026.101031

**Published:** 2026-06-10

**Authors:** Yestle Kim, Romina Fakhraei, John C. O’Donnell, Karissa Johnston, Melissa Bather, Reem Mustafa, Andrew R. Kennedy, Amreen Dinani

**Affiliations:** 1 Madrigal Pharmaceuticals, Inc, West Conshohocken, Pennsylvania; 2 Broadstreet HEOR, Vancouver, British Columbia, Canada; 3 Duke University Health System, Durham, North Carolina

**Keywords:** Metabolic Dysfunction–Associated Steatohepatitis, Cirrhosis, Progression, Health-Care Resource Use

## Abstract

**Background and Aims:**

Metabolic dysfunction–associated steatohepatitis (MASH) affects approximately 5% of adults globally and, without effective treatment, may progress to end-stage liver disease (ESLD), including compensated cirrhosis, decompensated cirrhosis (DC), hepatocellular carcinoma, and liver transplantation. Using real-world data, this study characterized the natural history and health-care impact of MASH among US adults.

**Methods:**

A retrospective cohort study was conducted using Optum’s deidentified Market Clarity Data (January 2021–March 2024). Noninvasive test (NIT) use at diagnosis (days 0–30) and during follow-up was described. Predictors of progression were estimated with modified Poisson regression, time to first ESLD was assessed with Cox models, and health-care resource utilization (HCRU) and costs with multivariable regression.

**Results:**

Among 49,983 patients with MASH, 16,359 (32.7%) had ESLD at baseline (compensated cirrhosis 3965; DC 11,406; hepatocellular carcinoma 514; liver transplantation 474). Of the 33,624 without baseline ESLD, 15.9% progressed; median time to first ESLD event was 10.6 months (interquartile range: 3.8–19.7), with DC the most frequent first event. Around diagnosis, 3771 patients (7.5%) underwent ≥1 NIT or imaging test, slightly higher with baseline ESLD than without (8.1% vs 7.3%); NIT/imaging use increased during follow-up. Older age, hypertension, type 2 diabetes, cardiovascular disease, sleep apnea, smoking, and thyroid disease were associated with higher progression risk. Patients who progressed had higher HCRU and costs than those who did not progress.

**Conclusion:**

In this large US cohort, progression from MASH to ESLD was associated with high HCRU and costs, particularly among patients with ESLD or subsequent progression. These findings highlight the need for earlier risk stratification and targeted care to mitigate burden.

## Introduction

Metabolic dysfunction-associated steatotic liver disease (MASLD) is the most common chronic liver disease worldwide, affecting approximately 30% of adult and is projected to exceed 55% by 2040.^1^^,^^2^ It is defined as hepatic steatosis with at least 1 cardiometabolic risk factor, and is strongly linked to obesity, insulin resistance, and type 2 diabetes mellitus (T2DM).^2^^,^^3^ Metabolic dysfunction–associated steatohepatitis (MASH) is the progressive form of MASLD, characterized by hepatic inflammation and fibrosis.^4^^,^^5^ Approximately 5% of adults are affected globally, with an estimated 10%–30% of patients with MASLD advancing to MASH.^3^, ^4^, ^5^ Recent data in middle-aged Americans reports a 14% prevalence of MASH, suggesting that the true prevalence may be higher.^6^ Without effective treatment and proper risk stratification, MASH may progress to end-stage liver disease (ESLD), including compensated cirrhosis (CC), decompensated cirrhosis (DC), hepatocellular carcinoma (HCC), and liver failure. Untreated MASH is also associated with elevated cardiovascular disease (CVD) risk and all-cause mortality.^7^, ^8^, ^9^, ^10^

Beyond clinical sequelae, MASH is linked to reduced health-related quality of life and substantial economic burden, with health-care resource utilization (HCRU) and costs rising sharply with advanced fibrosis and multimorbidity.^7^^,^^11^^,^^12^ Historically, MASH management has focused on lifestyle interventions such as diet, exercise, and weight loss. More recently, therapeutic advances have been made with the conditional US Food and Drug Administration approvals of semaglutide (Wegovy), a glucagon-like peptide-1 receptor agonist, and resmetirom (Rezdiffra), an oral, liver-directed thyroid hormone receptor beta agonist for adults with noncirrhotic MASH and moderate to advanced fibrosis.^13^

Despite rising prevalence, robust real-world evidence on MASH progression, its predictors, HCRU, and costs are limited.^10^ To address this evidence gap, we conducted a population-based study using Optum’s deidentified Market Clarity Data (Optum Market Clarity) to characterize the natural history and health-care impact of diagnosed MASH among US adults. Our objectives were to (1) describe patient characteristics around the time of MASH diagnosis (eg, comorbidities, cardiometabolic risk factors, and the use of noninvasive tests [NITs] and imaging), (2) evaluate disease progression over time by changes in NIT scores and incidence of advanced liver outcomes (eg, CC, DC, HCC, liver transplantation [LT]), and (3) quantify the impact of progression to ESLD on HCRU and costs.

## Methods

### Study Design and Data Source

We conducted a retrospective cohort study of US adults using Optum Market Clarity, which links electronic health records (EHRs) with administrative medical and pharmacy claims.^14^ The dataset includes demographics, encounters, diagnoses, procedures, laboratory results, and medication orders/dispensing (Supplementary Methods). The study observation window was January 1, 2019, to March 31, 2025. Patients with MASH (International Classification of Diseases, 10th Revision [ICD-10] code K75.8) were identified between January 1, 2021, and March 31, 2024. The index date (day 0) was the first recorded diagnosis of MASH. We assessed comorbidities, metabolic risk factors, and medication use during the 12-month preindex period (days −365 to 0). We ascertained exclusion criteria and prior cirrhosis or ESLD during the 24-month preindex period (days −730 to 0). Recognizing that hepatic assessments (eg, NITs) may occur shortly after diagnosis, we included an index testing window from the MASH diagnosis date (day 0) through day 30 to capture key assessments (eg, liver function tests, NITs, imaging tests, and biopsies) obtained around diagnosis. Follow-up for these assessments began on day 31 and continued until disenrollment, a >30-day coverage gap, or death (whichever came first). Follow-up for progression outcomes, HCRU, and costs began on day 1 and continued until censor. ESLD stages were ordered by severity as CC, DC, HCC, and LT. See Supplementary Figure 1 for the study schematic.

### Study Population

Eligible patients were adults aged ≥18 years with either ≥1 inpatient claim listing MASH as a primary or secondary diagnosis, or ≥2 outpatient claims for MASH on different dates, identified using ICD-10 code K75.81. Exclusions (Supplementary Table 1) were assessed during the preindex lookback (days −730 to 0) and included alternative chronic liver diseases and other prespecified conditions; patients with any resmetirom (Rezdiffra) exposure in this window were also excluded. Additional requirements included ≥24 months of enrollment prior to index and ≥12 months of continuous follow-up after index (unless deceased). Patients who developed a prespecified exclusionary condition during follow-up were removed from the cohort at the time the condition was first recorded.

The study population was stratified into 2 primary groups based on the presence or absence of ESLD at baseline. Among patients with baseline ESLD, we assigned a mutually exclusive ESLD category based on the most advanced ESLD manifestation observed in the 2-year preindex period (hierarchy: LT > HCC > DC > CC), using diagnosis and procedure codes (Supplementary Table 2). Patients without baseline ESLD were monitored for progression to ESLD stages during follow-up.

### Outcomes

Study outcomes included the use of NITs, imaging tests, blood-based biomarker tests, and liver biopsy during the index diagnosis testing window and over follow-up, stratified by ESLD status (Supplementary Table 2). Among patients without baseline ESLD, liver disease progression was defined using validated diagnosis and procedure codes as first occurrence of CC, DC, HCC, LT, or death. Progression was defined by the first qualifying event after index and assumed to be unidirectional (ie, no reversion to earlier stages). All time-to-event analyses used this “first ESLD event” definition; for descriptive tables, progressors were additionally grouped into mutually exclusive ESLD categories using the LT > HCC > DC > CC hierarchy.

Baseline characteristics and covariates were evaluated as potential predictors of progression. NIT and imaging results (eg, magnetic resonance elastography [MRE], transient elastography, enhanced liver fibrosis [ELF]) were captured during the index diagnosis testing window and over follow-up. We derived the following 2 composite indices from routinely collected laboratory data (alanine aminotransferase [ALT], aspartate aminotransferase [AST], platelet count): Fibrosis-4 index (FIB-4) and Aspartate Aminotransferase to Platelet Ratio Index (APRI; definitions in Supplementary Table 2). Indices were summarized separately around the index testing window (days 0–30) and at the first available follow-up (day ≥31) using available-case data (no imputation). For each index, we reported the number of patients with all required components within the window, the median (interquartile range [IQR]), and the proportions below and above clinically relevant thresholds defined in practice guidelines.^15^^,^^16^

A composite clinical end point was defined as first occurrence of CC (among those without baseline ESLD), DC, HCC, LT, or all-cause mortality.

HCRU included inpatient admissions, emergency department visits, outpatient encounters (generalist and specialist), and medication dispensing. Encounter definitions were standardized by claim identifier and date to reflect billing granularity (Supplementary Table 2).

### Statistical Analysis

Patient characteristics, testing patterns, treatments and outcomes were summarized descriptively; categorical variables were expressed as counts and percentages, while continuous variables were expressed as means with standard deviations or medians with IQRs. For descriptive summaries of ESLD progression, patients were grouped according to the worst ESLD stage reached during follow-up using the predefined hierarchy (LT > HCC > DC > CC), whereas all time-to-event end points were defined using time from index to the first ESLD event of any type. Kaplan–Meier methods were used to summarize time to first event and to report median time-to-event (IQR) for each progression outcome category. For first ESLD event subtypes (CC, DC, HCC, LT), cumulative incidence functions under a competing-risks framework were estimated and plotted. For evaluation of progression, a multivariable Cox proportional hazards model was used to estimate adjusted hazard ratios (aHRs) with 95% confidence intervals (CIs), adjusting for demographic, regional, comorbidity, and clinical risk factors.

Cardiometabolic risk factors, including metabolic syndrome, hypertension, dyslipidemia, T2DM, and obesity, were evaluated overall and by subgroup, and patients were further classified by the number of risk factors present. Other comorbidities such as polycystic ovary syndrome, menopause, and sleep apnea were also assessed (Supplementary Table 2).

HCRU counts were modeled with multivariable negative binomial regression (offset for follow-up person-time) to estimate marginal means. For marginal means, 95% CIs were derived using a nonparametric patient-level bootstrap with 500 resamples (refitting all model components per replicate and using percentile intervals). Costs (presented in 2024 United States Dollar) were analyzed per-person-per-year (PPPY) using generalized linear models with log link and gamma distribution; two-part models were used if zero-inflation was evident. Because cost distributions are typically right skewed, both mean and median PPPY costs were reported. Multivariable models included adjustment for prespecified covariates (baseline demographics, comorbidities, and prior health utilization) and included robust variance estimators. Missingness in labs/NITs was handled using available-case analysis; components required to compute composite indices were not imputed. Analyses were conducted using R (version 4.5.1; R Foundation for Statistical Computing, Vienna, Austria). A two-sided α = 0.05 defined statistical significance.

### Sensitivity Analyses

To ensure the robustness of our findings, various sensitivity analyses were performed. First, we expanded the index diagnosis testing window to 15 days before index through 90 days after index to better capture NITs and laboratory measures in settings without same-day testing. Next, we expanded the MASH cohort to include MASLD patients (ICD-10-CM K76.0) and repeated several analyses with this larger cohort to contextualize disease burden and progression among the broader steatotic liver disease spectrum. Lastly, we repeated the progression and HCRU/cost analyses after reclassifying patients who experienced an ESLD event within the first 30 days after index as having baseline ESLD, to assess the potential impact of misclassifying prevalent ESLD as incident progression.^17^

This study used deidentified data from Optum Market Clarity that are compliant with the Health Insurance Portability and Accountability Act; therefore, institutional review board review was not required. All work adhered to the principles of the Declaration of Helsinki. Data access was governed by a data-use agreement with Optum.

Reporting followed the Reporting of studies Conducted using Observational Routinely collected health Data statement, an extension of the Strengthening the Reporting of Observational Studies in Epidemiology guidelines for observational studies using routinely collected health data^18^; see Supplementary Methods for completed checklist.

## Results

### Baseline Characteristics

**Figure 1:**
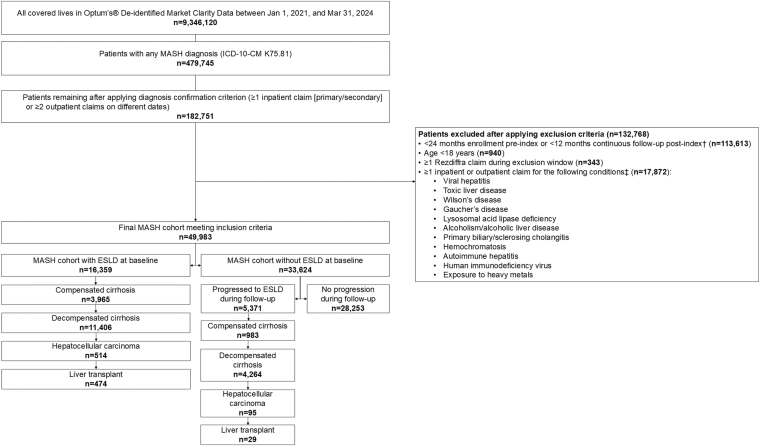
Study cohort diagram. ESLD, end-stage liver disease; ICD-10-CM, International Classification of Diseases 10th revision Clinical Modification; MASH, metabolic dysfunction–associated steatohepatitis; T2DM, type 2 diabetes mellitus. †This criteria is applied to all patients unless the patient had died during this time. ‡Diagnostic codes specified in Supplementary Table 1.

From over 9 million individuals in Optum Market Clarity during the study period, 49,983 patients with MASH met inclusion criteria and comprised the study cohort (Figure 1). At baseline, 16,359 (32.7%) had evidence of ESLD—3965 with CC, 11,406 with DC, 514 with HCC, and 474 with prior LT. Among those without ESLD at baseline (n = 33,624), 5371 (15.9%) progressed to ESLD during follow-up and 28,253 did not. Median (IQR) follow-up was slightly longer for patients without ESLD at baseline (2.3 [1.7–3.1] years) than for those with ESLD (2.1 [1.5–2.9] years).

Baseline demographic and clinical characteristics, comorbidities, and metabolic risk factors are shown in Table 1. At index, patients with ESLD were older (mean age 63.3 years vs 55.4 years) and more likely to be female (63.3% vs 57.9%) compared with those without ESLD. A higher mean (standard deviation) comorbidity burden was seen in patients with ESLD at baseline on both the weighted Elixhauser Comorbidity Index (7.1 [9.3] vs 1.7 [6.9]) as well as the Diabetes Complications Severity Index (1.6 [1.5] vs 0.7 [1.0]). These patients exhibited a higher prevalence of CVD, anemia, and abdominal/pelvic pain as compared to those without ESLD at baseline. Metabolic risk factors, including obesity, T2DM, hypertension, and dyslipidemia, were common in both groups, though hypertension and T2DM were more frequent among patients with ESLD.

**Table 1:** Baseline Characteristics of Patients Diagnosed With MASH by Baseline ESLD and Progression Status

Characteristic	Total (n = 49,983)	With ESLD at baseline (n = 16,359)	Without ESLD at baseline (n = 33,624)	With progression (n = 5371)	Without progression (n = 28,253)
	n (%)^a^	n (%)^a^	n (%)^a^	n (%)^a^	n (%)^a^
Length of follow-up (y)					
Mean (SD)	2.3 (0.9)	2.2 (1.0)	2.4 (0.9)	2.5 (0.9)	2.4 (0.9)
Median (IQR)	2.2 (1.6–3.0)	2.1 (1.5–2.9)	2.3 (1.7–3.1)	2.5 (1.8–3.3)	2.3 (1.6–3.0)
Sociodemographic information					
Age at index (y)					
Mean (SD)	58.0 (13.9)	63.3 (12.6)	55.4 (13.7)	59.0 (13.6)	54.7 (13.6)
Median (IQR)	59 (49–68)	64 (56–73)	56 (47–65)	60 (50–69)	56 (46–64)
Age category (y)					
18–44 y	8545 (17.1)	1353 (8.3)	7192 (21.4)	815 (15.2)	6377 (22.6)
45–64 y	24,799 (49.6)	6943 (42.4)	17,856 (53.1)	2589 (48.2)	15,267 (54.0)
≥65 y	16,639 (33.3)	8063 (49.3)	8576 (25.5)	1967 (36.6)	6609 (23.4)
Sex					
Female	29,809 (59.6)	10,354 (63.3)	19,455 (57.9)	3271 (60.9)	16,184 (57.3)
Male	20,139 (40.3)	5989 (36.6)	14,150 (42.1)	2097 (39.0)	12,053 (42.7)
Unknown	35 (0.1)	16 (0.1)	19 (0.1)	3 (0.1)	16 (0.1)
Race					
White	39,072 (78.2)	13,546 (82.8)	25,526 (75.9)	4241 (79.0)	21,285 (75.3)
Black	2159 (4.3)	563 (3.4)	1596 (4.7)	228 (4.2)	1368 (4.8)
Asian	1816 (3.6)	351 (2.1)	1465 (4.4)	190 (3.5)	1275 (4.5)
Other/unknown	6936 (13.9)	1899 (11.6)	5037 (15.0)	712 (13.3)	4325 (15.3)
Ethnicity					
Hispanic	6454 (12.9)	1879 (11.5)	4575 (13.6)	676 (12.6)	3899 (13.8)
Non-Hispanic	37,106 (74.2)	12,583 (76.9)	24,523 (72.9)	4032 (75.1)	20,491 (72.5)
Unknown	6423 (12.9)	1897 (11.6)	4526 (13.5)	663 (12.3)	3863 (13.7)
Geographic region					
Northeast	11,415 (22.8)	3143 (19.2)	8272 (24.6)	1218 (22.7)	7054 (25.0)
Midwest	16,667 (33.3)	6129 (37.5)	10,538 (31.3)	1779 (33.1)	8759 (31.0)
South	13,697 (27.4)	4638 (28.4)	9059 (26.9)	1492 (27.8)	7567 (26.8)
West	5925 (11.9)	1752 (10.7)	4173 (12.4)	610 (11.4)	3563 (12.6)
Unknown	2279 (4.6)	697 (4.3)	1582 (4.7)	272 (5.1)	1310 (4.6)
Insurance type					
Commercial	17,435 (34.9)	4907 (30.0)	12,528 (37.3)	1813 (33.8)	10,715 (37.9)
Medicaid	1742 (3.5)	621 (3.8)	1121 (3.3)	187 (3.5)	934 (3.3)
Medicare	6279 (12.6)	3274 (20.0)	3005 (8.9)	702 (13.1)	2303 (8.2)
Clinical characteristics					
BMI, kg/m^2^	14,203 (28.4)	4987 (30.5)	9216 (27.4)	1446 (26.9)	7770 (27.5)
Mean (SD)	34.8 (7.9)	34.8 (8.2)	34.8 (7.6)	35.4 (8)	34.7 (7.6)
Median (IQR)	33.7 (29.4–39.1)	33.6 (29–39.4)	33.7 (29.6–38.9)	34.3 (29.7–39.5)	33.6 (29.5–38.8)
BMI categories, kg/m^2^					
<25	919 (1.8)	401 (2.5)	518 (1.5)	97 (1.8)	421 (1.5)
25–<30	3078 (6.2)	1085 (6.6)	1993 (5.9)	285 (5.3)	1708 (6.0)
≥30	10,206 (20.4)	3501 (21.4)	6705 (19.9)	1064 (19.8)	5641 (20.0)
Unknown	35,780 (71.6)	11,372 (69.5)	24,408 (72.6)	3925 (73.1)	20,483 (72.5)
Comorbidities					
Cardiometabolic risk factors					
Metabolic syndrome	10,233 (20.5)	3508 (21.4)	6725 (20.0)	1070 (19.9)	5655 (20.0)
Hypertension	31,029 (62.1)	11,999 (73.3)	19,030 (56.6)	3515 (65.4)	15,515 (54.9)
Dyslipidemia	37,825 (75.7)	12,617 (77.1)	25,208 (75.0)	4129 (76.9)	21,079 (74.6)
Obesity	33,045 (66.1)	11,136 (68.1)	21,909 (65.2)	3615 (67.3)	18,294 (64.8)
T2DM	23,277 (46.6)	10,307 (63.0)	12,970 (38.6)	2595 (48.3)	10,375 (36.7)
≥1 cardiometabolic risk factor	46,980 (94.0)	15,631 (95.5)	31,349 (93.2)	5075 (94.5)	26,274 (93.0)
≥2 cardiometabolic risk factors	39,612 (79.3)	14,014 (85.7)	25,598 (76.1)	4374 (81.4)	21,224 (75.1)
≥3 cardiometabolic risk factors	28,695 (57.4)	11,133 (68.1)	17,562 (52.2)	3219 (59.9)	14,343 (50.8)
Other comorbidities of interest					
Abdominal/pelvic pain	16,415 (32.8)	6719 (41.1)	9696 (28.8)	1892 (35.2)	7804 (27.6)
Anemia	9819 (19.6)	5777 (35.3)	4042 (12.0)	975 (18.2)	3067 (10.9)
Bariatric surgery	371 (0.7)	118 (0.7)	253 (0.8)	31 (0.6)	222 (0.8)
Cardiovascular disease	21,011 (42.0)	9831 (60.1)	11,180 (33.3)	2401 (44.7)	8779 (31.1)
Fatigue/insomnia	15,737 (31.5)	6623 (40.5)	9114 (27.1)	1729 (32.2)	7385 (26.1)
Peptic ulcer disease, dyspepsia, GERD, esophagitis	21,200 (42.4)	8871 (54.2)	12,329 (36.7)	2498 (46.5)	9831 (34.8)
Renal impairment	7758 (15.5)	4554 (27.8)	3204 (9.5)	800 (14.9)	2404 (8.5)
Sleep apnea	15,715 (31.4)	5960 (36.4)	9755 (29.0)	1868 (34.8)	7887 (27.9)
Smoking (current or past)	10,775 (21.6)	4923 (30.1)	5852 (17.4)	1274 (23.7)	4578 (16.2)
T1DM	1307 (2.6)	724 (4.4)	583 (1.7)	165 (3.1)	418 (1.5)
Thyroid disease	12,373 (24.8)	4821 (29.5)	7552 (22.5)	1428 (26.6)	6124 (21.7)
Thyroid cancer	355 (0.7)	116 (0.7)	239 (0.7)	40 (0.7)	199 (0.7)
Vitamin D deficiency	14,561 (29.1)	4691 (28.7)	9870 (29.4)	1672 (31.1)	8198 (29.0)
PCOS^b^	1026 (2.1)	217 (1.3)	809 (2.4)	97 (1.8)	712 (2.5)
Menopause^b^	2926 (5.9)	913 (5.6)	2013 (6.0)	325 (6.1)	1688 (6.0)
End-stage renal disease	563 (1.1)	471 (2.9)	92 (0.3)	27 (0.5)	65 (0.2)
Elixhauser Comorbidity Index					
Unweighted					
Mean (SD)	3.9 (2.7)	5.6 (2.9)	3.1 (2.1)	4 (2.4)	3 (2)
Median (IQR)	3 (2–5)	5 (3–7)	3 (2–4)	4 (2–5)	3 (2–4)
Weighted					
Mean (SD)	3.5 (8.2)	7.1 (9.3)	1.7 (6.9)	3.2 (7.8)	1.4 (6.7)
Median (IQR)	2 (−2 to 7)	6 (0–13)	1 (−3 to 6)	2 (−2 to 7)	0 (−3 to 5)
Diabetes Complications Severity Index (DCSI)					
Mean (SD)	1.0 (1.3)	1.6 (1.5)	0.7 (1.0)	1.1 (1.3)	0.6 (1.0)
Median (IQR)	1.0 (0–2)	1.0 (0–2)	0 (0–1)	1.0 (0–2)	0 (0–1)
Medication use at baseline					
GLP-1 receptor agonists (any)	7748 (15.5)	2925 (17.9)	4823 (14.3)	917 (17.1)	3906 (13.8)
Semaglutide	3832 (7.7)	1333 (8.1)	2499 (7.4)	442 (8.2)	2057 (7.3)
Liraglutide	1383 (2.8)	529 (3.2)	854 (2.5)	149 (2.8)	705 (2.5)
Dulaglutide	2879 (5.8)	1158 (7.1)	1721 (5.1)	368 (6.9)	1353 (4.8)
Tirzepatide	425 (0.9)	132 (0.8)	293 (0.9)	36 (0.7)	257 (0.9)
Exenatide	312 (0.6)	124 (0.8)	188 (0.6)	45 (0.8)	143 (0.5)
Lixisenatide	101 (0.2)	53 (0.3)	48 (0.1)	10 (0.2)	38 (0.1)
Statin use (any)	22,961 (45.9)	8260 (50.5)	14,701 (43.7)	2639 (49.1)	12,062 (42.7)
SGLT2 inhibitors (any)	5876 (11.8)	2615 (16)	3261 (9.7)	704 (13.1)	2557 (9.1)
Canagliflozin	434 (0.9)	178 (1.1)	256 (0.8)	49 (0.9)	207 (0.7)
Dapagliflozin	1326 (2.7)	561 (3.4)	765 (2.3)	145 (2.7)	620 (2.2)
Empagliflozin	2920 (5.8)	1219 (7.5)	1701 (5.1)	339 (6.3)	1362 (4.8)
Ertugliflozin	147 (0.3)	55 (0.3)	92 (0.3)	14 (0.3)	78 (0.3)
Bexagliflozin	0 (0.0)	0 (0.0)	0 (0.0)	0 (0.0)	0 (0.0)
Clopidogrel	1677 (3.4)	904 (5.5)	773 (2.3)	231 (4.3)	542 (1.9)

BMI, body mass index; DCSI, Diabetes Complications Severity Index; ESLD, end-stage liver disease; GERD, gastroesophageal reflux disease; GLP-1, glucagon-like peptide-1; IQR, interquartile range; MASH, metabolic dysfunction–associated steatohepatitis; PCOS, polycystic ovary syndrome; SD, standard deviation; T1DM, type 1 diabetes mellitus; T2DM, type 2 diabetes mellitus.

a n (%) unless otherwise specified.

b PCOS and menopause were identified among women only.

Among patients without ESLD at baseline, those who progressed to ESLD during follow-up were generally older (59.0 vs 54.7 years), more likely to be female (60.9% vs 57.3%) and had longer median (IQR) length of follow-up (2.5 [1.8–3.3] vs 2.3 [1.6–3.0] years) compared with those without ESLD. Patients who progressed to ESLD also had higher rates of most cardiometabolic risk factors, including hypertension (65.4% vs 54.9%), T2DM (48.3% vs 36.7%), obesity (67.3% vs 64.8%), and dyslipidemia (76.9% vs 74.6%) compared with those who remained free of progression.

### Treatment Patterns at Baseline

At baseline, 15.5% of the entire cohort had evidence of glucagon-like peptide-1 receptor agonists use, most commonly semaglutide (7.7%) and dulaglutide (5.8%), with other agents used infrequently (Table 1). Nearly half of the cohort were on a statin (45.9%), whereas SGLT2 inhibitor use was lower (11.8%), with empagliflozin (5.8%) being the most frequently used drug in that class. Use of clopidogrel was uncommon (3.4%). Across all classes, medication use was consistently higher among patients with baseline ESLD than those without ESLD, including any glucagon-like peptide-1 receptor agonists (17.9% vs 14.3%), any SGLT2 inhibitor (16.0% vs 9.7%), any statin (50.5% vs 43.7%), and clopidogrel (5.5% vs 2.3%; Table 1).

### Noninvasive Tests, Imaging, Liver Tests, Biopsy, and Fibrosis Indices

The use of NITs, imaging, liver function blood tests, and liver biopsies during the index diagnosis window (days 0–30) varied by baseline ESLD status (Table 2). Overall, 3771 (7.5%) patients received ≥1 NIT or imaging test; uptake was higher with baseline ESLD (1318/16,359; 8.1%) than without (2453/33,624; 7.3%). Of those patients, 868 received only magnetic resonance imaging (MRI; 1.7%); uptake was higher with baseline ESLD (549/16,359; 3.4%) than without (319/33,624; 1.0%). Transient elastography was seen in 4.0% and 5.5%, respectively, while ELF was not observed.

**Table 2:** Use of Noninvasive Tests (NITs), Liver Function Blood Tests, and Liver Biopsies Within MASH Index Diagnosis Testing Window (Day 0, 30) and During Follow-Up by Baseline ESLD Status

Test type	Within index diagnosis testing window (day 0, 30)		During follow-up (day 31, end of follow-up)	
	With ESLD at baseline (n = 16,359)	Without ESLD at baseline (n = 33,624)	With ESLD at baseline (n = 16,359)	Without ESLD at baseline (n = 33,624)
	n (%)^a^	n (%)^a^	n (%)^a^	n (%)^a^
Noninvasive tests (NITs) and imaging				
At least 1 NIT or imaging test (any)	1318 (8.1)	2453 (7.3)	4333 (26.5)	6050 (18.0)
Transient elastography	650 (4.0)	1835 (5.5)	1623 (9.9)	3817 (11.4)
Enhanced liver fibrosis (ELF)	0 (0.0)	0 (0.0)	19 (0.1)	64 (0.2)
Magnetic resonance elastography (MRE)	37 (0.2)	85 (0.3)	212 (1.3)	366 (1.1)
LiverMultiScan	n < 5^b^	n < 5^b^	7 (<0.1)	44 (0.1)
Fibrosure/Fibrotest	87 (0.5)	236 (0.7)	214 (1.3)	535 (1.6)
Magnetic resonance imaging (MRI)	603 (3.7)	414 (1.2)	2881 (17.6)	2168 (6.4)
Liver biopsy	694 (4.2)	1253 (3.7)	746 (4.6)	1242 (3.7)
Liver function blood tests				
Albumin and total protein	2389 (14.6)	3841 (11.4)	3779 (23.1)	6647 (19.8)
Bilirubin	2373 (14.5)	3868 (11.5)	3721 (22.7)	6552 (19.5)
Alkaline phosphatase (ALP)	2286 (14.0)	3639 (10.8)	3655 (22.3)	6455 (19.2)
Aspartate aminotransferase (AST)	2312 (14.1)	3764 (11.2)	3677 (22.5)	6506 (19.3)
Alanine aminotransferase (ALT)	2326 (14.2)	3788 (11.3)	3686 (22.5)	6521 (19.4)
Gamma-glutamyltransferase (GGT)	177 (1.1)	355 (1.1)	1901 (11.6)	3027 (9.0)
L-lactate dehydrogenase (LD)	222 (1.4)	114 (0.3)	1811 (11.1)	2703 (8.0)
Platelet count (PLT)	1701 (10.4)	2270 (6.8)	3492 (21.3)	6712 (20.0)
Prothrombin time (PT)	1288 (7.9)	702 (2.1)	2711 (16.6)	3334 (9.9)
Fasting glucose	21 (0.1)	67 (0.2)	111 (0.7)	314 (0.9)
Laboratory-based indices				
FIB-4, computable n (%)	1942 (11.8)	2662 (7.9)	3053 (18.7)	5963 (17.7)
Median (IQR)	2.6 (1.4–5.3)	1.2 (0.8–1.8)	2.3 (1.3–4.4)	1.2 (0.8–1.7)
FIB-4 <1.3^c^	410 (21.1)	1503 (56.5)	765 (25.0)	3311 (55.5)
FIB-4 ≥2.67^c^	960 (49.4)	282 (10.6)	1305 (42.7)	487 (8.2)
APRI, computable n (%)	1947 (11.9)	2678 (8.0)	3062 (18.7)	5971 (17.7)
Median (IQR)	0.5 (0.3–1.1)	0.3 (0.2–0.5)	0.4 (0.3–0.9)	0.3 (0.2–0.4)
APRI <1^c^	1433 (73.6)	2444 (91.3)	2438 (79.6)	5722 (95.8)
APRI ≥2^c^	203 (10.4)	107 (4.0)	192 (6.3)	46 (0.8)

ALP, alkaline phosphatase; ALT, alanine aminotransferase; APRI, Aspartate Aminotransferase to Platelet Ratio Index; AST, aspartate aminotransferase; CT, computed tomography; ELF, enhanced liver fibrosis; ESLD, end-stage liver disease; FIB-4, Fibrosis-4 index; GGT, gamma-glutamyltransferase; LD, L-lactate dehydrogenase; MRE, magnetic resonance elastography; MRI, magnetic resonance imaging; NIT, noninvasive test; PLT, platelet count; PT, prothrombin time.

a n (%) unless otherwise specified.

b Per agreement with data provider, results with n < 5 have been redacted.

c Percentages are calculated among patients with a computable index in the given window/stratum (ie, the denominator is those patients). The extreme categories (eg, FIB-4 <1.3 and ≥2.67; APRI <1 and ≥2) will not sum to 100% because the indeterminate band is not shown.

During follow-up (day 31+), NIT and imaging test utilization increased. Among patients with baseline ESLD, 26.5% (4333/16,359) had ≥1 NIT or imaging test vs 18.0% (6050/33,624) without baseline ESLD (Table 2). Of those patients, 14.4% (2360/16,369) received only MRI with baseline ESLD vs 4.4% (1465/33,624) without baseline ESLD. MRI was frequently performed in patients with baseline ESLD (17.6%), whereas transient elastography predominated without baseline ESLD (11.4%). Other modalities—including MRE (1.3% and 1.1%), FibroSure/FibroTest (1.3% and 1.6%), LiverMultiScan (<0.1% and 0.1%), and ELF (0.1% and 0.2%)—were rare. Liver biopsy was slightly higher with baseline ESLD (4.6%) than without (3.7%).

Laboratory assessments were consistently more common in patients with baseline ESLD (Table 2). Within the index window, bilirubin and albumin/total protein were obtained in 14.5% and 14.6% with ESLD vs 11.5% and 11.4% without; AST and ALT were each measured in 14.1% and 14.2% vs 11.2% and 11.3%. Liver biopsies were similar in both groups (4.2% vs 3.7%). During follow-up, frequency of testing increased, including albumin/total protein (23.1% vs 19.8%), bilirubin (22.7% vs 19.5%), AST (22.5% vs 19.3%), and ALT (22.5% vs 19.4%), with the largest differential for prothrombin time (16.6% with ESLD vs 9.9% without). Additionally, the proportion of patients with computable indices increased at follow-up: both FIB-4 and Aspartate Aminotransferase to Platelet Ratio Index were calculable for 18.7% of patients with baseline ESLD and 17.7% without.

### Disease Progression Events and Predictors of Disease Progression

Among patients without ESLD at baseline (n = 33,624), 15.9% (n = 5371) progressed to an ESLD event during follow-up. DC was the most frequent first event (12.7%; n = 4264), followed by CC (2.9%; n = 983), HCC (0.3%; n = 95), and LT (0.1%; n = 29). The median time to first ESLD event was 10.6 months (IQR: 3.8–19.7), with time to CC shortest (8.1 months, 2.5–16.9) and time to LT longest among ESLD components (14.7 months [IQR: 9.2–24.4]). All-cause death occurred in 1.8% (n = 601; median 22.3 months [IQR: 10.5–33.5]).

Progression was slightly higher among patients with multiple comorbidities and occurred on a similar timeline (Table 3). The proportion progressing to any ESLD event was 11.0% among those with ≥2 cardiometabolic risk factors (n = 4374/39,612; median time from index 10.9 months [IQR: 4.0–20.1]), 11.2% with ≥3 risk factors (n = 3219/28,695; 10.7 months [IQR: 3.8–19.8]), 10.9% with obesity (n = 3615/33,045; 10.3 months [IQR: 3.6–19.7]), and 11.1% with T2DM (n = 2595/23,277; 10.8 months [IQR: 3.9–19.5]). Within these subgroups, DC remained the dominant first event (∼8%–9%), CC occurred in ∼2%, and HCC and LT were rare (≤0.2% and ≤0.1%, respectively); median times from index were closely aligned with the overall cohort (CC ∼8 months; DC ∼11 months).

**Table 3:** Progression Events Among Patients With MASH Without Baseline ESLD, Overall, and in High-Risk Subgroups

Progression category	Definition	Without ESLD at baseline (n = 33,624)		≥2 cardiometabolic risk factors (n = 39,612)^a^		≥3 cardiometabolic risk factors (n = 28,695)^a^		Obesity (n = 33,045)		T2DM (n = 23,277)	
		n (%)	Median (IQR)^b^	n (%)	Median (IQR)^b^	n (%)	Median (IQR)^b^	n (%)	Median (IQR)^b^	n (%)	Median (IQR)^b^
Compensated cirrhosis (CC)	CC, no prior DC/HCC/LT	983 (2.9)	8.1 (2.5–16.9)	799 (2.0)	8.3 (2.5–17.5)	602 (2.1)	7.9 (2.2–16.0)	672 (2.0)	8.1 (2.5–16.4)	530 (2.3)	8.2 (2.7–16.2)
Decompensated cirrhosis (DC)	DC, no prior HCC/LT	4264 (12.7)	11.1 (4.1–20.3)	3475 (8.8)	11.4 (4.5–20.7)	2546 (8.9)	11.3 (4.3–20.5)	2862 (8.7)	10.8 (3.9–20.3)	2001 (8.6)	11.4 (4.4–20.2)
Hepatocellular carcinoma (HCC)	HCC, no prior LT	95 (0.3)	10.2 (4.2–18.5)	79 (0.2)	10.6 (4.8–18.5)	56 (0.2)	10.7 (5.9–19.4)	64 (0.2)	10.1 (5.0–18.9)	49 (0.2)	10.6 (5.6–22.2)
Liver transplantation (LT)	LT, regardless of previous state	29 (0.1)	14.7 (9.2–24.4)	21 (0.1)	18.1 (12.1–24.9)	15 (0.1)	21.0 (18.0–28.6)	17 (0.1)	19.1 (13.6–26.7)	15 (0.1)	19.1 (12.4–25.8)
Death	All-cause	601 (1.8)	22.3 (10.5–33.5)	535 (1.4)	22.7 (11.4–33.8)	399 (1.4)	23.5 (12.3–34.1)	363 (1.1)	23.8 (12.1–34.6)	350 (1.5)	22.2 (11.3–33.9)
Composite ESLD progression	First of CC, DC, HCC, LT	5371 (15.9)	10.6 (3.8–19.7)	4374 (11.0)	10.9 (4.0–20.1)	3219 (11.2)	10.7 (3.8–19.8)	3615 (10.9)	10.3 (3.6–19.7)	2595 (11.1)	10.8 (3.9–19.5)

CC, compensated cirrhosis; DC, decompensated cirrhosis; ESLD, end-stage liver disease; HCC, hepatocellular carcinoma; IQR, interquartile range; LT, liver transplantion; T2DM, type 2 diabetes mellitus.

a Cardiometabolic risk factors include metabolic syndrome, hypertension, dyslipidemia, T2DM, and obesity.

b Median time-to-event (months).

**Figure 2:**
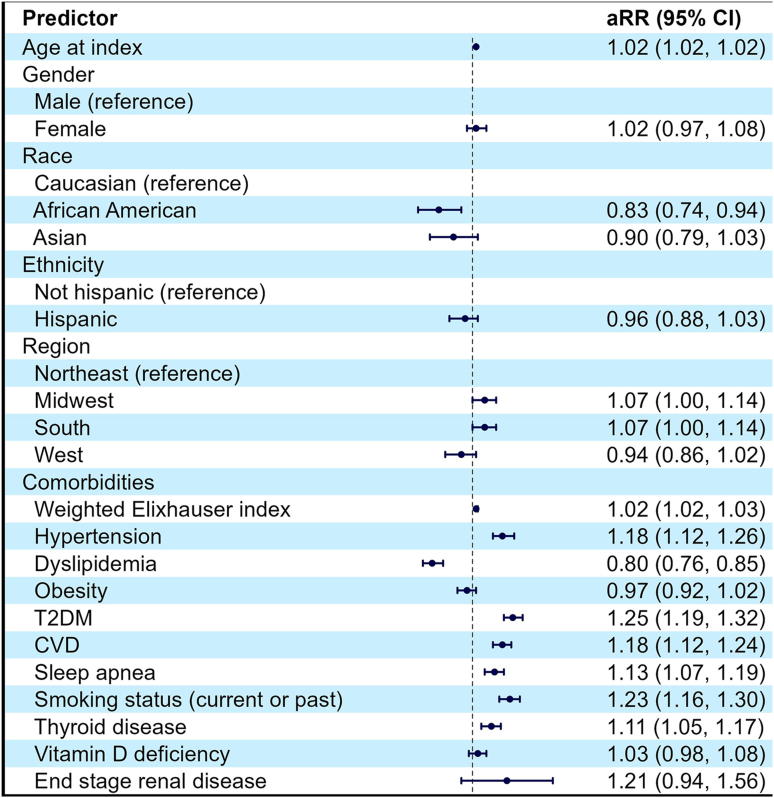
Predictors of composite liver disease progression: adjusted relative risks (aRR)^†^. aRR, adjusted relative risk; CI, confidence interval; CVD, cardiovascular disease; RR, relative risk; T2DM, type 2 diabetes mellitus. ^†^Model adjusted for age, gender, race, ethnicity, region, Elixhauser Comorbidity Index, hypertension, dyslipidemia, obesity, T2DM, smoking, CVD, sleep apnea, thyroid disease, vitamin D deficiency, and end-stage renal disease. See Supplementary Table 3 for unadjusted values.

In adjusted analyses (Figure 2; Supplementary Table 9), older age was associated with a slightly higher risk of progression (adjusted risk ratio [aRR]: 1.02 [95% CI: 1.02–1.02]). Compared with Caucasian patients, African American patients had lower risk of disease progression (aRR: 0.83 [95% CI: 0.74–0.94]). Relative to the Northeast, progression risk was modestly higher in the Midwest (aRR: 1.07 [95% CI: 1.00–1.14]) and South regions (aRR: 1.07 [95% CI: 1.00–1.14]). Presence of metabolic and clinical factors independently associated with higher progression risk included hypertension (aRR: 1.18 [95% CI: 1.12–1.26]), type 2 diabetes (aRR: 1.25 [95% CI: 1.19–1.32]), CVD (aRR: 1.18 [95% CI: 1.12–1.24]), sleep apnea (aRR: 1.13 [95% CI: 1.07–1.19]), smoking (aRR: 1.23 [95% CI: 1.16–1.30]), and thyroid disease (aRR: 1.11 [95% CI: 1.05–1.17]). Obesity, vitamin D deficiency and end-stage renal disease were not associated with a higher risk of progression after adjustment (Supplementary Table 9). Dyslipidemia showed an inverse association after adjustment (aRR: 0.80 [95% CI: 0.76–0.85]).

### Cumulative Incidence of Death and First End-Stage Liver Disease Events

**Figure 3:**
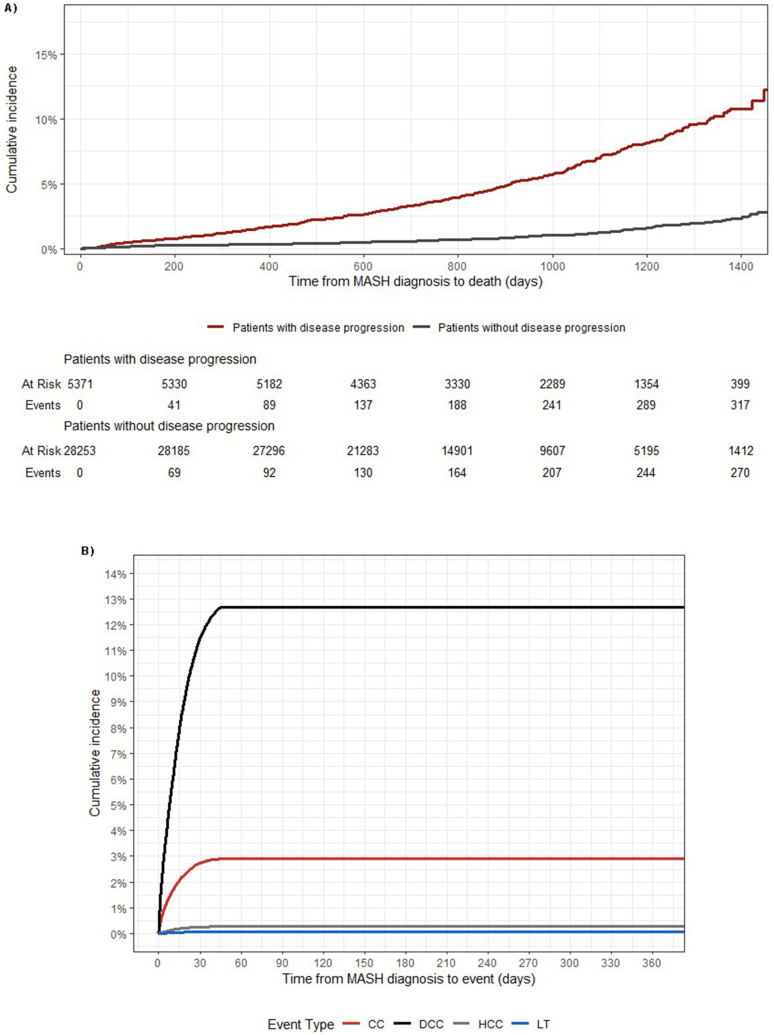
Cumulative incidence of (A) all-cause death after MASH diagnosis, by progression status and (B) first progression event type after MASH diagnosis among those without ESLD at baseline. CC, compensated cirrhosis; DCC, decompensated cirrhosis; ESLD, end-stage liver disease; HCC, hepatocellular carcinoma; LT, liver transplantation; MASH, metabolic dysfunction–associated steatohepatitis. Note: (A) X-axis truncated at 1,420 days for illustration purposes to avoid small–risk-set artifacts near study end; (B) X-axis truncated at 365 days for illustration purposes; curves plateau thereafter.

Cumulative incidence of all-cause mortality was consistently higher among MASH patients who progressed than among nonprogressors (Figure 3A). The curves separated early and continued to diverge over follow-up. By 24 months (730 days), mortality was 3.51% in progressors vs 0.59% in nonprogressors; by 47 months (1420 days), it was 10.75% vs 2.64%. Despite a smaller at-risk population at baseline (5371 vs 28,253), more deaths accrued among progressors by approximately 4 years (317 vs 270).

Within the first year after MASH diagnosis, the cumulative incidence of first progression was highest for DC (∼13%), followed by CC (∼3%), HCC (<0.5%), and LT (<0.2%). Most events occurred early, within 30–60 days of diagnosis, after which the curves plateaued (Figure 3B).

### Health-Care Resource Utilization and Costs

During follow-up, patients with ESLD at baseline (n = 16,359) incurred substantially higher PPPY total costs than those without ESLD (n = 33,624): mean $134,674 (median $71,527 [IQR: 27,483–164,022]) vs $70,159 (median $30,237 [IQR: 10,108–75,334]) (Supplementary Figure 2A); adjusted mean $27,372 (95% CI: 25,975–28,554) vs $21,489 (95% CI: 20,483–22,401) (Supplementary Table 4).

HCRU was consistently higher with baseline ESLD (Supplementary Figure 2B): adjusted inpatient admissions 0.6 vs 0.3 PPPY; ED visits 2.2 vs 1.5; outpatient visits 54.8 vs 45.0; GP visits 5.3 vs 4.3; specialist visits 47.5 vs 38.8; and total medication dispensations 93.8 vs 88.2 (Supplementary Table 4;). Component costs (PPPY-adjusted mean) showed the same pattern, ESLD vs no ESLD: inpatient $11,001 vs $6497; ED $1155 vs $843; outpatient $8156 vs $6710; GP $208 vs $179; specialist $8033 vs $6622; total medications $21,014 vs $13,289.

Among patients without ESLD at baseline, those who progressed (n = 5371) had higher total costs than nonprogressors (n = 28,253) (Supplementary Figure 2A and B): adjusted mean $36,498 (95% CI 34,444–38,358) vs $16,305 (15,438–17,027) and greater utilization: inpatient admissions 0.7 vs 0.2 PPPY; ED visits 2.3 vs 1.1; outpatient visits 57.8 vs 40.4; GP visits 4.6 vs 3.2; specialist visits 51.8 vs 35.6; and total medication dispensations 89.1 vs 78.5 (Supplementary Table 5). Component costs (PPPY-adjusted mean) were likewise higher with progression: inpatient $18,506 vs $3308; ED $1647 vs $618; outpatient $11,326 vs $5826; GP $262 vs $172; specialist $11,194 vs $5729; and medications $8471 vs $6300 (Supplementary Table 5).

### Sensitivity Analyses

In the sensitivity analysis for which an extended index diagnosis testing window was considered (day −15 to day 90), overall ascertainment of testing increased but the relative patterns by ESLD status were unchanged compared with the main 0 to 30-day baseline window (Supplementary Table 6). Among patients with baseline ESLD, any NIT or imaging test rose from 8.1% (1318/16,359) to 13.8% (2258/16,359), with MRI remaining the predominant modality (3.7%–7.5%); liver biopsy increased slightly (4.2%–5.8%). Among those without ESLD, any NIT or imaging test rose from 7.3% (2453/33,624) to 11.1% (3740/33,624), with transient elastography remaining most common (5.5%–7.8%); liver biopsy increased from 3.7% to 4.8%. Liver function test capture increased across analytes in both groups, but fasting glucose remained rarely recorded (<1%).

In the MASLD sensitivity cohort, 462,224 patients met the inclusion criteria (Supplementary Figure 3). At baseline, 76,950 (16.6%) had ESLD: 7328 with CC; 67,658 with DC; 965 with HCC; and 999 with LT. The remaining 385,274 had no baseline ESLD; of these, 57,873 (15.0%) progressed to ESLD during follow-up (CC n = 4355 [7.5%]; DC n = 52,459 [90.6%]; HCC n = 695 [1.2%]; LT n = 364 [0.6%]), while 327,401 did not progress.

Testing patterns in the MASLD cohort paralleled the primary MASH analysis, with higher use of NITs, imaging tests, liver biopsy, and liver function tests among patients with baseline ESLD and increased testing from the diagnosis window to follow-up (see Supplementary Table 7, Supplementary Results). Among patients without baseline ESLD, progression patterns in the MASLD cohort were directionally consistent with the MASH cohort (Supplementary Table 8, Supplementary Results). The median time to the first ESLD event was 9.7 months. Among individual outcomes, LT occurred earliest (median 8.1 months; IQR: 3.0–18.3), while HCC occurred latest (median 11.4 months; IQR: 3.5–21.5). All-cause death was observed in 1.9% of patients (n = 6300), with a median time of 18.9 months (IQR: 8.6–30.8).

Broadening to MASLD yielded a similar pattern of predictors of progression as in the primary MASH cohort: higher risk with older age, greater comorbidity burden, hypertension, T2DM, CVD, sleep apnea, and smoking. Relative to MASH, 2 differences were notable as follows: Asian race showed a stronger protective association (aRR: 0.77 [95% CI: 0.73–0.80]), and end-stage renal disease exhibited a stronger positive association with progression (aRR: 1.46 [95% CI: 1.36–1.56]). Overall, the direction and magnitude of most associations were consistent with our primary analysis (Supplementary Table 9).

Patterns in health-care use and costs in the larger MASLD cohort mirrored primary MASH findings. Patients with baseline ESLD had higher adjusted total PPPY costs than those without ESLD, with consistently greater spending across components (Supplementary Table 10). Among patients without baseline ESLD, those who progressed had markedly higher adjusted total costs and higher HCRU across all settings than nonprogressors (Supplementary Table 11).

In a sensitivity analysis that reclassified patients who experienced an ESLD event within 30 days of MASH diagnosis (n = 563) as having baseline ESLD, the proportion with progression to ESLD decreased from 15.9% to 14.5%. Among those without ESLD at baseline, the median time to first ESLD progression event increased from 10.6 months (IQR: 3.8–19.7) in the primary analysis to 12.1 months (IQR: 5.8–21.0) after reclassifying these early events. Predictors of ESLD progression were consistent with primary analysis findings (Supplementary Table 12). HCRU and cost outcomes showed a similar pattern to the primary findings, with even higher utilization and costs among patients with ESLD at baseline and among those who progressed after reclassifying early ESLD events as baseline ESLD (Supplementary Tables 13 and 14).

Lastly, in a post-hoc sensitivity analysis of the MASH cohort with additional adjustment for baseline statin use, the association between dyslipidemia and risk of progression was unchanged, with a similar risk estimate to the primary analysis (aRR: 0.81 [0.76–0.86]).

## Discussion

In this large, real-world cohort of 49,983 US adults with MASH, one-third had evidence of ESLD at baseline, and among those without ESLD, 16% progressed during follow-up. DC was the most common first progression event, and the median time to first ESLD event was short (∼11 months). This rapid progression in observed diagnoses is in contrast to prior natural history studies that have reported overall progression from MASH to cirrhosis or ESLD of approximately 7%–12% over follow-up periods of 7–20 years.^19^, ^20^, ^21^, ^22^ The observed substantially higher prevalence and timing of progression status in our study likely reflects the capture of clinical recognition, rather than true disease onset and reflects real-world risk following identification. Thus, these findings highlight a rapid trajectory from MASH identification to clinically meaningful liver disease and echo prior evidence linking advancing fibrosis with stepwise increases in decompensation and mortality.^23^ From a health-system perspective, patients with baseline ESLD and those who progressed incurred substantially higher utilization and costs across care settings, consistent with prior US real-world studies showing sharp cost increases at advanced fibrosis, decompensation, and HCC.^24^ Importantly, among patients without ESLD at baseline, those who progressed reached mean annual costs comparable to baseline ESLD patients within a short interval after diagnosis. These data underscore the clinical and economic urgency of earlier identification and risk-directed management.

Use of NITs and imaging tests around diagnosis was modest overall, though higher with baseline ESLD; during follow-up, NIT and imaging test utilization increased in both strata. Given current guidance that recommends NIT utilization when MASH is suspected and subsequent routine first-line fibrosis risk stratification (eg, FIB-4) in primary and specialty care,^15^^,^^25^^,^^26^ these data suggest a gap between guidance and practice and an opportunity to standardize early risk assessment, particularly for high-risk patients. Furthermore, liver biopsy was infrequently used in this cohort, with only 4% of patients undergoing the procedure for diagnosis. This is consistent with prior reports that biopsy is performed in only about 10% of patients before a MASH diagnosis, despite its role as the reference standard for confirming liver disease.^15^^,^^27^^,^^28^ Other economic analyses demonstrate that sequential use of NITs in primary care is an effective way to rationalize secondary care referrals and is associated with substantial cost savings of over 40%.^29^ Further evidence suggests that when primary care physicians identify patients at risk of advanced fibrosis via NITs and refer them to specialists, diagnosis of the condition increases fourfold.^30^ When promptly followed by intensive lifestyle interventions or pharmacological treatments, research supports this as a cost-effective approach to managing MASH.^30^

Several baseline characteristics were independently associated with higher progression risk in our study, including older age, hypertension, T2DM, CVD, sleep apnea, smoking and thyroid disease. These findings are consistent with the broader MASLD literature. A multicenter, biopsy-based study (n = 511) reported that hypertension and T2DM were independently associated with advanced fibrosis (≥F3).^31^ An individual-participant meta-analysis linked obstructive sleep apnea severity with hepatic steatosis.^32^ Cohort and meta-analytic data associate smoking with greater fibrosis severity and worse outcomes.^33^ CVD reflects a higher-risk cardiometabolic phenotype that tracks with advanced liver disease.^34^ Thyroid dysfunction has been associated with MASLD and higher odds of advanced fibrosis.^35^ Lastly, the inverse association with dyslipidemia is unlikely to reflect true biological protection, as noted in prior observational analyses.^36^^,^^37^ Although the post-hoc sensitivity analysis that additionally adjusted for baseline statin use yielded a risk estimate similar to the primary analysis, residual confounding is likely and may explain the observed protective association.

Mortality diverged sharply by progression status as follows: cumulative mortality separated early and continued to widen, and despite a smaller at-risk population, more deaths accrued among progressors than nonprogressors. Preventing progression, and identifying patients approaching decompensation, is therefore critical to reduce death and downstream health-care use.^16^ These observations align with a US multicenter prospective cohort showing stepwise increases in decompensation and mortality with advancing fibrosis and ∼7-fold higher all-cause mortality after any decompensation.^38^

Strengths of this study include leveraging a large, real-world cohort of 49,983 adults with clinically identified MASH drawn from a well-validated database linking EHR and claims, enabling comprehensive capture of diagnoses, procedures, laboratory tests, imaging/NITs, health-care utilization, and costs. The use of linked EHR claims further supports laboratory and baseline risk profiles observed in prior studies and provides actionable insight into patient stratification, gaps between clinical guidance and real-world practice, and opportunities to refine management strategies. Unlike prior research,^10^^,^^17^ we used a 24-month preindex lookback to better identify prevalent cirrhosis and ESLD, reducing misclassification at index and yielding a more stable baseline for risk stratification and cost estimation. In addition, we excluded patients exposed to resmetirom (Rezdiffra) to characterize natural history and economic burden in the pretreatment era in the United States. We applied a rigorous cohort design with an extended baseline/exclusion horizon to minimize misclassification of prevalent ESLD and to define a cleaner at-risk population, alongside a standardized first-event ordering for progression end points (CC, DC, HCC, and LT). Importantly, the patterns of progression, HCRU, and costs were robust when the cohort was expanded to MASLD, with similar gradients by baseline ESLD and by progression status.

### Limitations

This study has several limitations. First, although we used a large, well-validated EHR–claims database, data accuracy depends on the quality and completeness of source documentation and coding. Misclassification is possible for exposures, comorbidities, and outcomes; BMI and some laboratory measures were sparsely populated, which may impair risk adjustment and subgroup definitions. In particular, very early ESLD “progression” events (eg, within 1 month of MASH diagnosis) likely reflect a lag between true disease onset and when an ICD code is first recorded, or patients whose first presentation is with decompensation, rather than rapid progression from incident MASH. To address this, we conducted a sensitivity analysis reclassifying early ESLD events as baseline ESLD, which produced results similar to the primary analysis, but some residual misclassification of undiagnosed or uncoded MASH is still likely. Second, based on a source population of more than 9 million individuals in Optum Market Clarity, we would expect more than 49,983 MASH diagnoses assuming a prevalence of 14% in middle-aged Americans with a similar median age to this cohort.^6^ It is likely that ICD-10-coded MASH diagnoses are under ascertained and preferentially recorded in patients with more advanced or clinically apparent disease. Consequently, the study cohort likely represents a higher severity population rather than the full spectrum of MASH. However, results of our sensitivity analysis using a broader MASLD population (n = 462,224; closely aligned with expected patient numbers) yielded similar results, which supports the robustness of our findings to alternative cohort definitions and partially mitigates concerns about underascertainment of ICD-coded MASH. As a result, observed event rates and time-to-event should be interpreted with caution as they may overstate progression rates in the general MASH population. Instead, these findings highlight a real-world signal for the need of earlier identification and intervention in routine care.

Additionally, as with any claims-based analysis, misclassification due to coding errors, potentially including upcoding to meet coverage criteria, may have biased estimates, particularly for ESLD component outcomes, by inflating apparent disease severity and progression. Next, differential surveillance (eg, imaging, NIT uptake) may have led to ascertainment bias, with higher test intensity increasing the likelihood of detecting progression. Although liver MRI was more frequently observed among patients with baseline ESLD than among those without, these findings should be interpreted with caution when assessing NIT and imaging utilization. In this population, MRI use likely reflects a mix of cross-sectional imaging for HCC surveillance, evaluation of portal hypertension, and MRI-PDFF for MASH assessment, which cannot be reliably differentiated due to the absence of specific CPT codes.^39^ Therefore, NIT and imaging utilization were not separated, and as a result NIT usage without imaging in the baseline ESLD cohort is likely lower. Consistent with this interpretation, MRE was infrequently used in the overall cohort. Generalizability may also be limited, as the study primarily reflects commercially insured US patients and may not represent uninsured, publicly insured, or non-US populations or other care settings. Finally, the study period overlaps with the COVID-19 pandemic, during which disruptions in health-care delivery and shifts in coding and utilization patterns could confound observed utilization and progression rates.

## Conclusion

In this large US real-world MASH cohort, progression risk was higher with older age and presence of cardiometabolic comorbidities. MASH was associated with substantial health-care costs, which increased markedly among patients with cirrhosis at baseline and among those who progressed during follow-up. The relatively low uptake of NITs and imaging tests at diagnosis highlights a practical opportunity to strengthen clinical care pathways, particularly in high-risk patients, to identify advanced disease earlier and expedite referral.

From a health-system perspective, prioritizing targeted fibrosis assessment and longitudinal monitoring in patients with obesity, T2DM, CVD, and related metabolic disorders may improve risk stratification and enable earlier intervention. Standardizing early testing, optimizing risk-factor control, and timely initiation of effective pharmacologic therapy may reduce decompensation, hospitalizations, and downstream costs. Future work should evaluate the cost-effectiveness of these strategies, assess whether patients receiving MASH-indicated therapies experience slower progression and better clinical outcomes, and examine subgroups to guide implementation at scale.

## Supplementary Materials

Supplementary Material

Extended PDF
