# Supplementary material for: Progression of Metabolic Dysfunction–Associated Steatohepatitis in US Adults Using Linked Records and Claims
Source: Gastro Hep Adv. 2026 Jun 10;5(9):101031. doi: 10.1016/j.gastha.2026.101031 (PMC13351128; doi:10.1016/j.gastha.2026.101031)
Supplement: Supplementary Material [file mmc1.pdf]

SUPPLEMENTARY MATERIALS

Supplementary Figure 1. Study design diagram

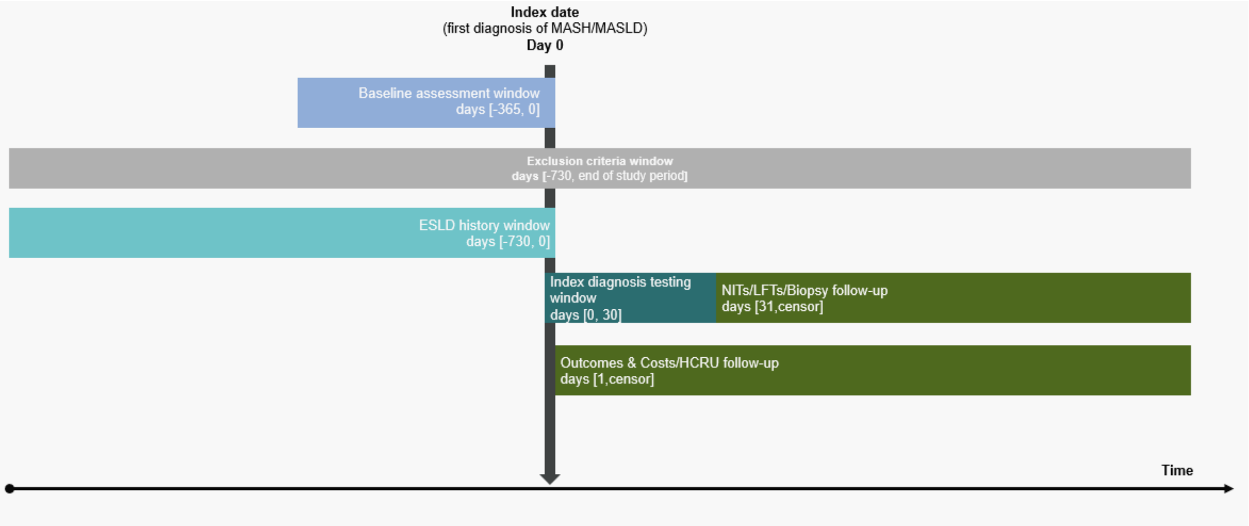

Abbreviations: HCRU, healthcare resource use; LFT, liver function test; MASH, metabolic dysfunction-associated steatohepatitis; MASLD, metabolic dysfunction-associated steatotic liver disease; NIT, non-invasive test.

Supplementary Figure 2a. Mean annual unadjusted per-person per-year healthcare costs by baseline ESLD and progression status

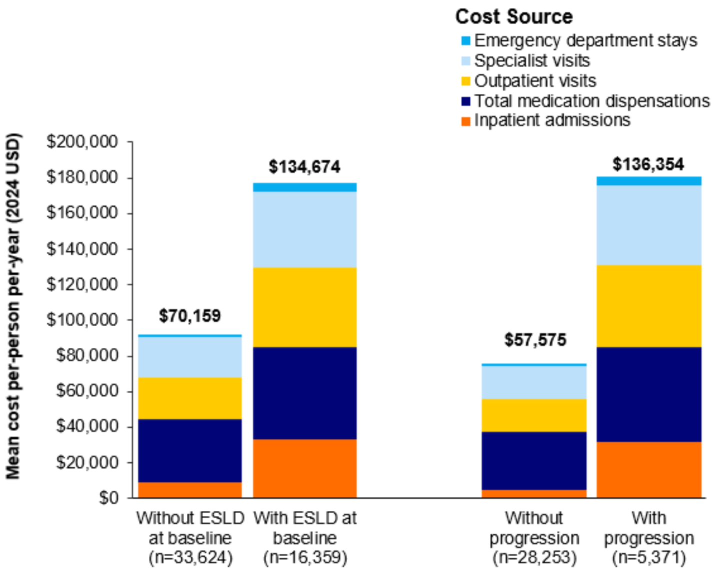

Abbreviations: ESLD, end stage liver disease; USD, United States dollar.

Supplementary Figure 2b. Unadjusted healthcare resource use by baseline ESLD and progression status

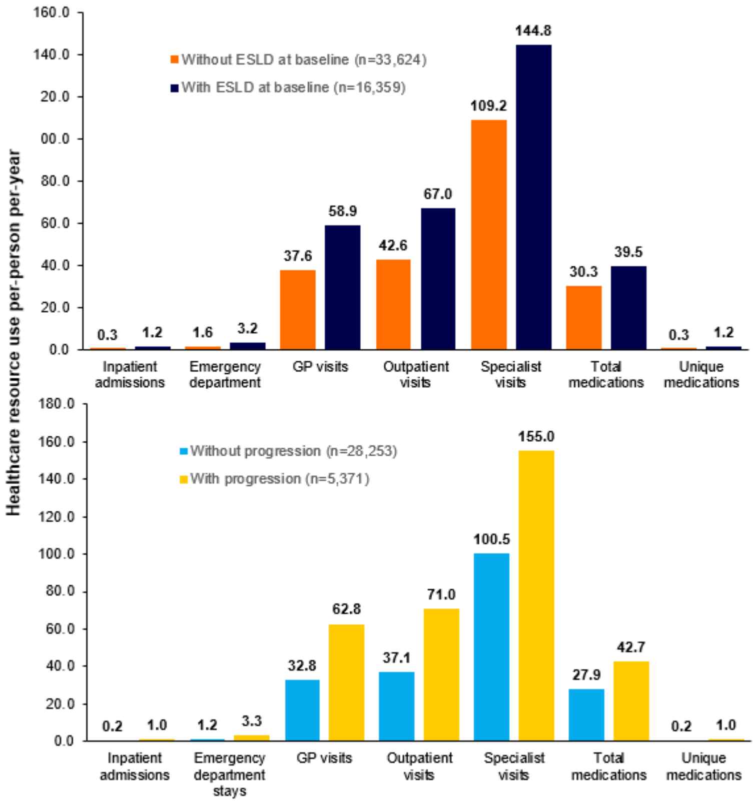

Abbreviations: ESLD, end stage liver disease; GP, general practitioner.

Supplementary Figure 3. Study flow diagram for sensitivity cohort creation (MASH and MASLD)

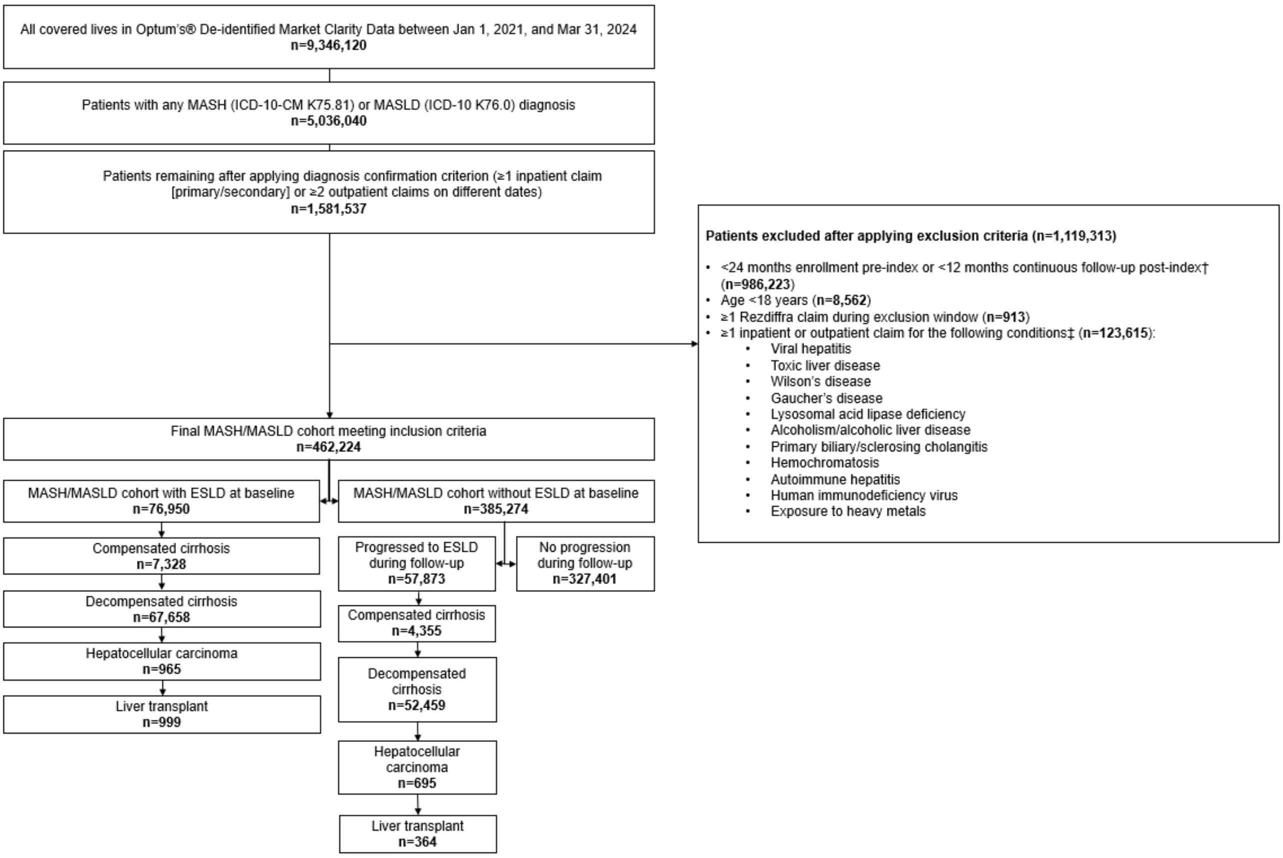

Abbreviations: ESLD, end stage liver disease; ICD-10-CM, International Classification of Diseases 10th revision Clinical Modification; MASH, metabolic dysfunction-associated steatohepatitis; MASLD, metabolic dysfunction-associated steatotic liver disease; T2DM, type 2 diabetes mellitus.
†This criteria is applied to all patients unless the patient had died during this time; ‡Diagnostic codes specified in Supplement Table 1.

Supplementary Table 1. Diagnostic codes for conditions that were excluded from study cohort

Exclusion criteria	Code type	Codes
Viral hepatitis[1]	ICD-10-CM	K75.3, B16.0, B16.1, B16.2, B16.9, B17.0, B18.0, B18.1, B19.10, B19.11, Z22.51, B17.10, B17.11, B18.2, B19.20, B19.21, Z22.52, B00.81, B15.0, B15.9, B17.2, B17.8, B17.9, B18.8, B18.9, B19.0, B19.9, B25.1, B26.81, B94.2, O98.411, O98.412, O98.413, O98.419, O98.42, O98.43, Z22.50, Z22.59
	HCPCS	G8459, G8461, G8463
Toxic liver disease[1]	ICD-10-CM	K71.0, K71.10, K71.11, K71.2, K71.3, K71.4, K71.50, K71.51, K71.6, K71.7, K71.8, K71.9
Wilson's disease[1]	ICD-10-CM	E83.01
Gaucher's disease[1]	ICD-10-CM	E75.22
Lysosomal acid lipase deficiency[1]	ICD-10-CM	E75.5, E75.6
Alcoholism / alcoholic liver disease[1]	ICD-10-CM	E24.4, F10.10, F10.120, F10.121, F10.129, F10.14, F10.150, F10.151, F10.159, F10.180, F10.181, F10.182, F10.188, F10.19, F10.20, F10.21, F10.220, F10.221, F10.229, F10.230, F10.231, F10.232, F10.239, F10.24, F10.250, F10.251, F10.259, F10.26, F10.27, F10.280, F10.281, F10.282, F10.288, F10.29, F10.920, F10.921, F10.929, F10.94, F10.950, F10.951, F10.959, F10.96, F10.97, F10.980, F10.981, F10.982, F10.988, F10.99, G31.2, G62.1, G72.1, I42.6, K29.20, K29.21, K70.0, K70.10, K70.11, K70.2, K70.3, K70.30, K70.31, K70.40, K70.41<br>K70.9, K85.2, K86.0, O35.4XX0, O35.4XX1, O35.4XX2, O35.4XX3, O35.4XX4, O35.4XX5, O35.4XX9, O99.310, O99.311, O99.312, O99.313, O99.314, O99.315
Primary biliary/sclerosing cholangitis[1]	ICD-10-CM	K74.3, K74.4, K74.5, K83.0
Hemochromatosis[1]	ICD-10-CM	E83.118, E83.119
Autoimmune hepatitis[2]	ICD-10-CM	K73.2, K75.4
Human immunodeficiency virus[3]	ICD-10-CM	B20.X, B21.X, B22.X, B23.X, B24, R75, Z21
Exposure to heavy metals[4]	ICD-10-CM	T56.X

Abbreviations: HCPCS, Healthcare Common Procedure Coding System; ICD-10-CM, International Classification of Diseases 10th revision Clinical Modification.

Supplementary Table 2. Codes and definitions used to define study variables

Study variable	Code type(s)	Code(s) or definitions
**End stage liver disease events**		
Compensated cirrhosis (CC)	ICD-10-CM	K74.60, K74.6, K74.4, K74.5, K74.69
Decompensated cirrhosis (DC)	CPT	49083, 49082, 43460, 43244, 43243, 43205, 43227, 43204, 49427, 49425, 37182, 43400, 49084, 78291, 49080, 49081, 49426, 37183, 43401, 43255, 37145, 37160, 37140, 37181, 37180
	HCPCS	C1040
	ICD-10-CM Diagnosis	K72.01, K72.00, K76.2, K72.11, K72.10, K22.8, I85.0, I85.00, I85.01, I85.10, I86.4, I98.20, I98.3, K92.2, K92.0, K72.91, K72.90, K76.81, K76.82, K76.6, K76.7, R17, R18.8, K92.1, J90, I85.11, K65.2, G93.40, G93.41, G93.49, K67, K65.9
	ICD-10-CM Procedures	0D9W00Z, 0D9W30Z, 0D9W40Z, 0D9W0ZZ, 0D9W0ZX, 0D9W3ZZ, 0D9W3ZX, 0D9W4ZZ, 0D9W4ZX, 0DL57DZ, 0DL58DZ
Liver transplant (LT)	CPT	47135, 47136
	DRG	005, 006, 405, 406, 407
	HCPCS	S2053
	ICD-10-CM Diagnosis	T86.40, T86.41, T86.42, T86.43, T86.49, Z48.23, Z94.4
	ICD-10-CM Procedures	0FY00Z0, 0FY00Z1, 0FY00Z2
Hepatic cell carcinoma (HCC)	ICD-10-CM Diagnosis	C22.0, C22.1, C22.8, C24.0
**Fibrosis staging tests**		
Liver biopsy[5]	CPT	37200, 47100, 47001, 47562, 47379, 47000
**Non-invasive tests (NITs)**		
Transient elastography[6]	CPT	91200
Abdominal ultrasound[6]	CPT	76700, 76705
Computed tomography[6]	CPT	74150, 74160, 74170, 74174, 74175, 74176, 74177, 74178
Magnetic resonance imaging[6]	CPT	74181, 74182, 74183, 74185
Magnetic resonance elastography[7†]	CPT	76391
LiverMultiScan[8]	CPT	0648T,0649T
ELF	CPT	81517
Fibrosure/Fibrotest	CPT	81596
**Laboratory-based indices**		
Fibrosis-4 (FIB-4)	Computed using laboratory values	$FIB4 = \frac{Age\ (years) \times AST\ level\ (U/L)}{Platelet\ count\ (10^9/L) \times \sqrt{ALT\ level\ (U/L)}}$
Aspartate aminotransferase-to-platelet ratio index (APRI)	Computed using laboratory values	$APRI = \frac{AST\ level\ (U/L)/AST\ (Upper\ Limit\ of\ Normal\ ‡)(U/L)}{Platelet\ count\ (10^9/L)} \times 100$
**Care settings**		
Emergency department[9]	Revenue code	0450, 0451, 0452, 0456, 0459, 0981
Observation unit[10-12]	Revenue code	0762
**Metabolic risk factors**		
Metabolic syndrome (MetS)[13,14]	ICD-10-CM	E88.810
	Laboratory values	Fasting glucose (≥5.6 mmol/L)<br>Triglycerides (≥1.7 mmol/L)

Study variable	Code type(s)	Code(s) or definitions
		HDL-c (<1.29 mmol/L in females; <1.03 mmol/L in males)<br>Systolic blood pressure (≥130 mmHg)<br>Diastolic blood pressure (≥85 mmHg)
	Anthropometric values	Waist circumference (> ethnicity specific values)<br>BMI (≥30kg/m^2)
Hypertension[15-17]	ICD-10-CM	I10, I11, I12, I13, I15
	Laboratory values	Systolic blood pressure (≥140 mmHg)<br>Diastolic blood pressure (≥90 mmHg)
Dyslipidemia[18-20]	ICD-10-CM	E78.0x, E78.1, E78.2, E78.3, E78.4x, E78.5, E78.6, E78.70, E78.89, E78.9, E88.2
	Laboratory values	LDL-c (>3.4 mmol/L)<br>HDL-c (<1.0 mmol/L)<br>Total cholesterol (>5.2 mmol/L)<br>Triglycerides (>1.7 mmol/L)
Obesity*[21,22]	ICD-10-CM	Z68.3x, Z68.4x, E66.x, E65
	Anthropometric values	BMI (≥30kg/m^2)<br>Waist circumference (> ethnicity specific values)
Type 2 diabetes[23,24]	ICD-10-CM	E11.x
	Laboratory values	HbA1c (≥48 mmol/mol)<br>Fasting plasma glucose (≥7.0 mmol/L)<br>2-hour plasma glucose on OGTT (≥11.1 mmol/L)
**Other comorbidities**		
Type 1 diabetes mellitus[23]	ICD-10-CM	E10, E10.3x (excluding E**.34X and E**.35x)
Anemia[25]	ICD-10-CM	D50.x, D53.9, D64.9
Cardiovascular disease[26]		
Rheumatic heart disease	ICD-10-CM	I01-I01.9, I02.0, I05-I09.9
Ischemic heart disease	ICD-10-CM	I20-I21.6, I21.9-I25.9, Z82.4-Z82.49
Cerebrovascular disease	ICD-10-CM	G45-G46.8, I60-I64, I64.1, I65-I69.998, Z82.3
Ischemic stroke	ICD-10-CM	G45-G46.8, I63-I63.9, I65-I66.9, I67.2-I67.848, I69.3-I69.4
Hemorrhagic stroke	ICD-10-CM	I60-I62.9, I67.0-I67.1, I69.0- I69.298
Hypertensive heart disease	ICD-10-CM	I11-I11.2, I11.9
Cardiomyopathy and myocarditis	ICD-10-CM	B33.2-B33.20, B33.22-B33.24, D86.85, I40-I41.8, I42-I43.8, I51.4-I51.6
Myocarditis	ICD-10-CM	B33.2-B33.20, B33.22-B33.24, D86.85, I40-I41.8, I51.4-I51.6
Alcoholic cardiomyopathy	ICD-10-CM	I42.6
Other cardiomyopathy	ICD-10-CM	I42.0-I42.5, I42.7-I43.8
Atrial fibrillation and flutter	ICD-10-CM	I48-I48.92
Peripheral artery disease	ICD-10-CM	I70.2-I70.92, I73-I73.9
Endocarditis	ICD-10-CM	B33.21, I33-I33.9, I38-I38.0, I39-I39.9
Other cardiovascular and circulatory diseases	ICD-10-CM	I30-I32.8, I34-I37.9, I51-I51.3, I51.7-I52.8, I72-I72.9, I77-I83.93, I86-I89.0, I89.9, I95.0-I95.1, I98, I98.8-I99.9, K75.1
Bariatric surgery[27]	ICD-10-PCS	0DB64Z3, 0D16479, 0D1647A, 0D1647B, 0D1647L, 0D164J9, 0D164JA, 0D164JB, 0D164JL, 0D164K9, 0D164KA, 0D164KB, 0D164KL, 0D164Z9, 0D164ZA, 0D164ZB, 0D164ZL, 0D16879, 0D1687A, 0D1687B, 0D1687L, 0D168J9, 0D168JA, 0D168JB, 0D168JL, 0D168K9, 0D168K9, 0D168KA, 0D168KB, 0D168KL, 0D168Z9, 0D168ZA, 0D168ZB, 0D168ZL

Study variable	Code type(s)	Code(s) or definitions
Fatigues/insomnia[§]	ICD-10-CM	R53.0, R53.1, R53.8x, F51, F51.0, F51.1, F51.11, F51.13, F51.19, F51.2, G47.0
Peptic ulcer disease, dyspepsia, GERD, esophagitis	ICD-10-CM	K20, K21, K25, K26, K27, K28, K30
	HCPCS	PBL, PBU, DR016, DR024
Sleep apnea[28]	ICD-10-CM	G47.3, G47.30, G47.30o, G47.31, G47.310, G47.33, G47.34, G47.37, G47.38o, G47.39, R06.81
Smoking, current or past[†29]	ICD-10-AM	F17.1, F17.2, Z72.0, Z86.43, F17.2, Z72.0
Thyroid disease[30]	ICD-10-CM	E02, E03, E05, E05.0, E06, E06.3,
Thyroid cancer[30]	ICD-10-CM	C73
Vitamin D deficiency[31]	ICD-10-CM	E55
PCOS[32]	ICD-10-CM	E28.2
Menopause[33¶]	ICD-10-CM	N95
	NDC (drug classes/generic drug names)[1]	Natural and semisynthetic estrogens
Renal impairment[34]	ICD-10-CM	N18, N18.1, N18.2, N18.3, N18.4, N18.5, N17, Z99.2, N49.2
	HCPCS codes	PBL, PBU, DR016, DR024
	NDC (drug classes/generic drug names)[1]	Dialysis solutions, electrolyte depleters, electrolyte maintenance, erythropoiesis-stimulating agents, kidney stone agents, mineral replacement, multivitamin preparations
End-stage-renal disease[35]	ICD-10-CM	I12.0, I13.11, N18.5, N18.6, Z49.31, Z49.32, Z91.151, Z91.158, Z99.2

Abbreviations: ALT, alanine aminotransferase; AST, aspartate aminotransferase; BMI, body mass index; CC, compensated cirrhosis; CKD, chronic kidney disease; CPT, Current Procedural Terminology; DC, decompensated cirrhosis; DRG, diagnosis-related group; ELF, enhanced liver fibrosis; HbA1c, hemoglobin A1c; HCC, hepatocellular carcinoma; HCPCS, Healthcare Common Procedure Coding System; HDL-c, high-density lipoprotein cholesterol; ICD-10-CM, International Classification of Diseases 10th Revision Clinical Modification; ICD-10-PCS, International Classification of Diseases 10th Revision Procedure Coding System; LDL-c, low-density lipoprotein cholesterol; LT, liver transplant; LSM, liver stiffness measure; NDC, National Drug Code; OGTT, oral glucose tolerance test; PCOS, polycystic ovary syndrome.

[†]Captures magnetic resonance imaging derived proton density fat fraction (MRIDPPFF), since MRIDPPFF does not have unique CPT codes[6,36]; [‡]A fixed value of 40 U/L was used for the Upper Limit of Normal; [§]Note that there will likely be limitations to capturing these with claims data, where positive predictive value is high but negative predictive value is low[22]; [¶]this algorithm used International Classification of Diseases, 9th Revision, Clinical Modification and these codes were converted to ICD-10-CM to reflect the current nomenclature.

Supplementary Table 3. Unadjusted and adjusted risk ratios (RRs) for predictors of disease progression (composite outcome) among patients with MASH

Predictor	Unadjusted RR (95% CI)	Adjusted RR (95% CI)†
**Age**		
Age at index	1.02 (1.02, 1.03)	1.02 (1.02, 1.02)
**Gender**		
Male (reference)	-	-
Female	1.13 (1.07, 1.18)	1.02 (0.97, 1.08)
**Race**		
Caucasian (reference)	-	-
African American	0.86 (0.77, 0.97)	0.83 (0.74, 0.94)
Asian	0.75 (0.66, 0.86)	0.90 (0.79, 1.03)
Other/Unknown	0.82 (0.77, 0.89)	0.96 (0.89, 1.08)
**Ethnicity**		
Not Hispanic (reference)	-	-
Hispanic	0.87 (0.81, 0.94)	0.96 (0.88, 1.03)
Unknown	0.87 (0.81, 0.94)	0.96 (0.88, 1.04)
**Region**		
Northeast (reference)	-	-
Midwest	1.17 (1.10, 1.25)	1.07 (1.00, 1.14)
South	1.14 (1.07, 1.22)	1.07 (1.00, 1.14)
West	0.99 (0.91, 1.08)	0.94 (0.86, 1.02)
Other/Unknown	1.18 (1.05, 1.33)	1.15 (1.03, 1.29)
**Comorbidities**		
Weighted Elixhauser index	1.03 (1.03, 1.04)	1.02 (1.02, 1.03)
Hypertension	1.50 (1.42, 1.58)	1.18 (1.12, 1.26)
Dyslipidemia	1.13 (1.07, 1.19)	0.80 (0.76, 0.85)
Obesity	1.07 (1.02, 1.13)	0.97 (0.92, 1.02)
T2DM	1.51 (1.44, 1.58)	1.25 (1.19, 1.32)
CVD	1.70 (1.63, 1.79)	1.18 (1.12, 1.24)
Sleep apnea	1.30 (1.24, 1.37)	1.13 (1.07, 1.19)
Smoking status (current or past)	1.51 (1.43, 1.60)	1.23 (1.16, 1.30)
Thyroid disease	1.27 (1.21, 1.34)	1.11 (1.05, 1.17)
Vitamin D deficiency	1.08 (1.03, 1.14)	1.03 (0.98, 1.08)
End stage renal disease	2.21 (1.69, 2.88)	1.21 (0.94, 1.56)

Abbreviations: CI, confidence interval; CVD, cardiovascular disease; N/A, not applicable; RR, risk ratio; T2DM, type 2 diabetes mellitus.
†Risk ratios adjusted for age, gender, race, ethnicity, region, Elixhauser index, hypertension, dyslipidemia, obesity, T2DM, smoking, CVD, sleep apnea, thyroid disease, vitamin D deficiency, and end-stage renal disease.

Supplementary Table 4. Healthcare resource utilization and costs during follow-up for MASH cohort (n=49,983), stratified by baseline ESLD

Category	With ESLD at baseline (n=16,359)				Without ESLD at baseline (n=33,624)			
	n (%)	Mean (SD)	Median (IQR)	Adjusted Mean (CI)†	n (%)	Mean (SD)	Median (IQR)	Adjusted Mean (CI)†
**Healthcare resource utilization (PPPY)**								
Inpatient admissions	4,924 (30.1)	1.2 (3.2)	0 (0, 1)	0.6 (0.5, 0.7)	2,754 (8.2)	0.3 (1.5)	0 (0, 0)	0.3 (0.3, 0.3)
ED	8,804 (53.8)	3.2 (6.5)	1 (0, 4)	2.2 (2.0, 2.3)	9,951 (29.6)	1.6 (4.3)	0 (0, 2)	1.5 (1.4, 1.6)
Outpatient visits	16,220 (99.2)	67.0 (72.8)	47 (23, 87)	54.8 (52.9, 56.9)	32,990 (98.1)	42.6 (48.7)	28 (14, 54)	45.0 (43.4, 46.6)
GP visits	16,114 (98.5)	58.9 (66.5)	40 (19, 76)	5.3 (4.9, 5.7)	32,532 (96.8)	37.6 (45.1)	24 (11, 48)	4.3 (4.0, 4.7)
Specialist visits	15,325 (93.7)	144.8 (154.5)	102 (44, 193)	47.5 (45.6, 49.3)	31,314 (93.1)	109.2 (123.2)	74 (32, 143)	38.8 (37.4, 40.3)
Medication dispensations (total)	15,325 (93.7)	39.5 (31.9)	33 (18, 54)	93.8 (90.1, 97.6)	31,314 (93.1)	30.3 (25.4)	25 (13, 41)	88.2 (84.9, 91.7)
Medication dispensations (unique)	4,924 (30.1)	1.2 (3.2)	0 (0, 1)	32.2 (31.2, 33.3)	2,754 (8.2)	0.3 (1.5)	0 (0, 0)	29.5 (28.6, 30.4)
**Healthcare costs (PPPY, 2024 USD)**								
Inpatient admissions	6,359 (38.9)	33,362 (83,640)	0 (0, 28,632)	11,001 (10,164, 11,929)	5,208 (15.5)	8,929 (40,854)	0 (0, 0)	6,497 (6,043, 6,999)
ED stays	10,714 (65.5)	4,989 (11,030)	1,468 (0, 5,663)	1,155 (1,093, 1,215)	15,275 (45.4)	1,947 (5,344)	0 (0, 1868)	843 (798, 891)
Outpatient visits	16,226 (99.2)	44,542 (70,696)	22,839 (8,839, 52,560)	8,156 (7,703, 8,629)	33,224 (98.8)	23,399 (43,871)	10,537 (4,024, 25,815)	6,710 (6,378, 7,092)
GP visits	10,816 (66.1)	2,051 (5,804)	428 (0, 2,007)	208 (181, 237)	19,704 (58.6)	1,179 (4,436)	183 (0, 1,142)	179 (156, 204)
Specialist visits	16,151 (98.7)	42,491 (69,575)	21,156 (7,871, 49,531)	8,033 (7,609, 8508)	32,996 (98.1)	22,220 (43,012)	9,649 (3,497, 24,451)	6,622 (6,294, 6,999)

Category	With ESLD at baseline (n=16,359)				Without ESLD at baseline (n=33,624)			
	n (%)	Mean (SD)	Median (IQR)	Adjusted Mean (CI)†	n (%)	Mean (SD)	Median (IQR)	Adjusted Mean (CI)†
Medication dispensations (total)‡	15,455 (94.5)	51,781 (166,379)	15,285 (2,457, 50,379)	21,014 (20,529, 21,472)	31,996 (95.2)	35,885 (103,226)	7,047 (1,098, 32,148)	13,289 (13,150, 13,433)
Total healthcare cost	16,337 (99.9)	134,674 (222,532)	71,527 (27,483, 164,022)	27,372 (25,975, 28,554)	33,579 (99.9)	70,159 (132,823)	30,237 (10,108, 75,334)	21,489 (20,483, 22,401)

Abbreviations: CI, confidence interval; ED, emergency department; ESLD, end stage liver disease; IQR, interquartile range; GP, general practitioner; PPPY, per-person-per-year; SD, standard deviation; USD, United States Dollar.
†Multivariable models were adjusted for adjusted for age, gender, race, ethnicity, region, Elixhauser index, hypertension, dyslipidemia, obesity, T2DM, smoking, CVD, sleep apnea, thyroid disease, vitamin D deficiency, end-stage renal disease and prior cost and healthcare resource utilization. ‡For medication dispensations, the full multivariable cost model did not converge because of overdispersion; therefore, adjusted means for this row are based on a model including only the exposure (i.e., history of ESLD at baseline) and not the full set of covariates.

Supplementary Table 5. Healthcare resource utilization and costs during follow-up for MASH cohort without baseline ESLD (n=33,624), stratified by progression status

Category	Progression ESLD (n=5,371)				No progression ESLD (n=28,253)			
	n (%)	Mean (SD)	Median (IQR)	Adjusted Mean (CI)†	n (%)	Mean (SD)	Median (IQR)	Adjusted Mean (CI)†
**Healthcare resource utilization (PPPY)**								
Inpatient admissions	729 (13.6)	1.0 (2.9)	0 (0, 1)	0.7 (0.6, 0.9)	2,025 (7.2)	0.2 (0.9)	0 (0, 0)	0.2 (0.1, 0.2)
ED	2,083 (38.8)	3.3 (6.9)	1 (0, 4)	2.3 (2.0, 2.7)	7,868 (27.8)	1.2 (3.5)	0 (0, 1)	1.1 (1.0, 1.3)
Outpatient visits	5,312 (98.9)	71 (71.4)	51 (27, 90)	57.8 (53.7, 62.3)	27,678 (98.0)	37.1 (40.8)	25 (12, 48)	40.4 (37.6, 43.4)
GP visits	5,266 (98.0)	62.8 (65.8)	44 (23, 81)	4.6 (3.9, 5.4)	27,266 (96.5)	32.8 (38.1)	22 (10, 42)	3.2 (2.7, 3.8)
Specialist visits	5,062 (94.2)	155 (156.6)	110 (55, 208)	51.8 (47.9, 56.1)	26,252 (92.9)	100.5 (113.8)	68 (30, 132)	35.6 (33.0, 38.5)
Medication dispensations (total)	5,062 (94.2)	42.7 (32.1)	36 (21, 58)	89.1 (81.9, 97.0)	26,252 (92.9)	27.9 (23.2)	23 (12, 38)	78.5 (72.3, 85.3)
Medication dispensations (unique)	729 (13.6)	1.0 (2.9)	0 (0, 1)	29.8 (27.9, 31.9)	2,025 (7.2)	0.2 (0.9)	0 (0, 0)	25.6 (24.0, 27.4)
**Healthcare costs (PPPY, 2024 USD)**								
Inpatient admissions	1,983 (36.9)	31,948 (82,020)	0 (0, 24,442)	18,506 (16,912, 20,263)	3,225 (11.4)	4,552 (24,244)	0 (0, 0)	3,308 (2,986, 3,632)
ED stays	3,613 (67.3)	4,917 (9,810)	1,594 (0, 5,738)	1,647 (1,561, 1,740)	11,662 (41.3)	1,382 (3,702)	0 (0, 1,354)	618 (580, 659)
Outpatient visits	5,349 (99.6)	46,414 (74,980)	24,600 (11,034, 53,481)	11,326 (10,384, 12,210)	27,875 (98.7)	19,024 (33,199)	8,911 (3,507, 21,301)	5,826 (5,348, 6,308)
GP visits	3,614 (67.3)	2,168 (7,187)	468 (0, 2,126)	262 (210, 423)	16,090 (56.9)	991 (3,657)	152 (0, 1,010)	172 (137, 273)
Specialist visits	5,329 (99.2)	44,245 (73,699)	22,967 (9,811, 50,664)	11,194 (10,267, 12,068)	27,667 (97.9)	18,033 (32,551)	8,130 (2,954, 20,042)	5,729 (5,273, 6,198)

Category	Progression ESLD (n=5,371)				No progression ESLD (n=28,253)			
	n (%)	Mean (SD)	Median (IQR)	Adjusted Mean (CI)†	n (%)	Mean (SD)	Median (IQR)	Adjusted Mean (CI)†
Medication dispensations (total)‡	5,169 (96.2)	53,075 (126,018)	15,178 (2,770, 51,347)	8,471 (8,458, 8,484)	26,827 (95.0)	32,617 (97,959)	5,896 (952, 28,943)	6,300 (6,300, 6,301)
Total healthcare cost	5,371 (100)	136,354 (191,352)	72,254 (29,287, 163,163)	36,498 (34,444, 38,358)	28,208 (99.8)	57,575 (114,214)	25,587 (8,616, 63,020)	16,305 (15,438, 17,027)

Abbreviations: CI, confidence interval; ESLD, end stage liver disease; IQR, interquartile range; GP, general practitioner; PPPY, per-person-per-year; SD, standard deviation; USD, United States Dollar.

†Multivariable models were adjusted for adjusted for age, gender, race, ethnicity, region, Elixhauser index, hypertension, dyslipidemia, obesity, T2DM, smoking, CVD, sleep apnea, thyroid disease, vitamin D deficiency, end-stage renal disease and prior cost and healthcare resource utilization. ‡For medication dispensations, the full multivariable cost model did not converge because of overdispersion; therefore, adjusted means for this row are based on a model including only the exposure (i.e., ESLD progression) and not the full set of covariates.

Supplementary Table 6. Sensitivity analysis - Use of non-invasive tests, liver function blood tests and liver biopsies with extended index diagnosis window [day -15, day 90]

Test type	Total (n=49,983)	With ESLD at baseline (n=16,359)	Without ESLD at baseline (n=33,624)
	n (%)	n (%)	n (%)
**Non-invasive tests (NITs) and imaging**			
At least one NIT or imaging test (any)	5,998 (12)	2,258 (13.8)	3,740 (11.1)
Transient elastography	3,619 (7.2)	978 (5.9)	2,641 (7.8)
Enhanced Liver Fibrosis (ELF)	0 (0)	0 (0)	0 (0)
Magnetic resonance elastography (MRE)	122 (0.24)	37 (0.2)	85 (0.3)
LiverMultiScan	8 (0.02)	n<5†	6 (0.02)
Fibrosure / Fibrotest	481 (1.0)	127 (0.8)	354 (1.1)
Magnetic resonance imaging (MRI)	2,105 (4.2)	1,231 (7.5)	874 (2.6)
**Liver biopsy**	2,560 (5.1)	945 (5.8)	1,615 (4.8)
**Liver function blood tests**			
Albumin and total protein	7,743 (15.5)	2,905 (17.8)	4,838 (14.4)
Bilirubin	7,679 (15.4)	2,864 (17.5)	4,815 (14.3)
Alkaline phosphatase (ALP)	7,515 (15.0)	2,815 (17.2)	4,700 (14.0)
Aspartate transaminase (AST)	7,613 (15.2)	2,841 (17.4)	4,772 (14.2)
Alanine transaminase (ALT)	7,644 (15.3)	2,850 (17.4)	4,794 (14.3)
Gamma-glutamyltransferase (GGT)	4,580 (9.2)	1,781 (10.9)	2,799 (8.3)
L-lactate dehydrogenase (LD)	4,273 (8.6)	1,702 (10.4)	2,571 (7.7)
Platelet count (PLT)	6,455 (12.9)	2,525 (15.4)	3,930 (11.7)
Prothrombin time (PT)	4,993 (10.0)	2,159 (13.2)	2,834 (8.4)
Fasting glucose	213 (0.4)	51 (0.31)	162 (0.5)

Abbreviations: ALP, alkaline phosphatase; ALT, alanine transaminase; AST, aspartate transaminase; CT, computed tomography; ELF, enhanced liver fibrosis; ESLD, end stage liver disease; GGT, gamma-glutamyltransferase; LD, L-lactate dehydrogenase; NIT, non-invasive test; PLT, platelet count; PT, prothrombin time.†Per agreement with data provider, results with n<5 have been redacted.

Supplementary Table 7. Sensitivity analysis - Use of non-invasive tests (NITs), liver function blood tests and liver biopsies at MASH/MASLD index diagnosis testing window (day 0,30) and during follow-up, by baseline ESLD status

Test type	Within index diagnosis testing window (day 0, 30)		During follow-up (day 31, end of follow-up)	
	With ESLD at baseline (n=76,950)	Without ESLD at baseline (n=385,274)	With ESLD at baseline (n=76,950)	Without ESLD at baseline (n=385,274)
	n (%)	n (%)	n (%)	n (%)
**Non-invasive tests (NITs) and imaging**				
At least one NIT or imaging test (any)	6,891 (9.0)	26,675 (6.9)	14,157 (18.4)	53,111 (13.8)
Transient elastography	2,574 (3.3)	11,434 (2.9)	5,323 (6.9)	24,920 (6.5)
Enhanced Liver Fibrosis (ELF)	0 (0.0)	0 (0.0)	62 (0.1)	310 (0.1)
Magnetic resonance elastography (MRE)	138 (0.2)	446 (0.1)	527 (0.7)	1,959 (0.5)
LiverMultiScan	n<5†	12 (<0.1)	32 (<0.1)	131 (<0.1)
Fibrosure / Fibrotest	415 (0.5)	1,954 (0.5)	866 (1.1)	4,668 (1.2)
Magnetic resonance imaging (MRI)	4,038 (5.2)	13,751 (3.6)	9,112 (11.8)	27,447 (7.1)
**Liver biopsy**	1,815 (2.4)	6,585 (1.7)	3,250 (4.2)	14,313 (3.7)
**Liver function blood tests**				
Albumin and total protein	10,349 (13.4)	42,257 (11.0)	17,218 (22.4)	74,655 (19.4)
Bilirubin	10,543 (13.7)	43,624 (11.3)	17,017 (22.1)	73,650 (19.1)
Alkaline phosphatase (ALP)	9,961 (12.9)	40,785 (10.6)	16,698 (21.7)	72,398 (18.8)
Aspartate transaminase (AST)	10,112 (13.1)	41,699 (10.8)	16,817 (21.9)	73,047 (19.0)
Alanine transaminase (ALT)	10,174 (13.2)	41,985 (10.9)	16,828 (21.9)	73,153 (19.0)
Gamma-glutamyltransferase (GGT)	711 (0.9)	3,178 (0.8)	8,369 (10.9)	34,047 (8.8)
L-lactate dehydrogenase (LD)	877 (1.1)	1,680 (0.4)	8,143 (10.6)	31,786 (8.3)
Platelet count (PLT)	7,680 (10.0)	27,839 (7.2)	15,947 (20.7)	75,753 (19.7)
Prothrombin time (PT)	4,104 (5.3)	7,090 (1.8)	10,302 (13.4)	36,897 (9.6)
Fasting glucose	106 (0.1)	716 (0.2)	547 (0.7)	3,104 (0.8)

Abbreviations: ALP, alkaline phosphatase; ALT, alanine transaminase; AST, aspartate transaminase; CT, computed tomography; ELF, enhanced liver fibrosis; ESLD, end stage liver disease; GGT, gamma-glutamyltransferase; LD, L-lactate dehydrogenase; MRE, magnetic resonance elastography; MRI, magnetic resonance imaging; NIT, non-invasive test; PLT, platelet count; PT, prothrombin time. †Per agreement with data provider, results with n<5 have been redacted.

Supplementary Table 8. Sensitivity analysis - Progression events among patients with MASH/MASLD without ESLD at baseline, overall and in high-risk subgroups

Progression category	Definition	Without ESLD at baseline (n=385,274)		≥2 cardiometabolic risk factors (n=132,966)†		≥3 cardiometabolic risk factors (n=243,094)†		Obesity (n=180,935)		T2DM (n=308,780)	
		n (%)	Median (IQR)‡	n (%)	Median (IQR)‡	n (%)	Median (IQR)‡	n (%)	Median (IQR)‡	n (%)	Median (IQR)‡
Compensated Cirrhosis (CC)	CC, no prior DC/HCC/LT	4,355 (0.9)	9.0 (2.8, 18.6)	3,403 (1.0)	9.5 (3.0, 19.2)	2,459 (1.1)	9.5 (3.0, 19.2)	2,769 (1.0)	9.3 (3.0, 18.7)	2,074 (1.4)	9.2 (2.9, 18.8)
Decompensated Cirrhosis (DC)	DC, no prior HCC/LT	52,459 (11.3)	9.7 (2.9, 19.0)	38,758 (11.8)	10.1 (3.1, 19.3)	26,787 (12.2)	10.1 (3.2, 19.3)	32,019 (11.4)	9.6 2.9, 18.9)	19,365 (12.6)	10.4 (3.4, 19.5)
Hepatocellular carcinoma (HCC)	HCC, no prior LT	695 (0.2)	11.4 (3.5, 21.5)	550 (0.2)	12.2 (4.0, 22.3)	401 (0.2)	12.3 (3.8, 23.0)	416 (0.1)	11.3 (3.4, 22.0)	338 (0.2)	12.4 (4.6, 22.7)
Liver Transplant (LT)	LT, regardless of previous state*	364 (0.1)	8.1 (3.0, 18.3)	275 (0.1)	8.7 (3.1, 18.3)	204 (0.1)	8.2 (2.9, 18.2)	232 (0.1)	9.1 (3.4, 18.5)	164 (0.1)	9.3 (3.2, 18.7)
Death	All-cause	7,592 (1.6)	19.1 (8.6, 30.7)	6,300 (1.9)	18.9 (8.6, 30.8)	4,654 (2.1)	19.0 (8.5, 31.0)	4,380 (1.6)	19.1 (8.3, 30.7)	3,771 (2.5)	19.3 (8.9, 31.6)
Composite ESLD progression	First of CC, DC, HCC, LT	57,873 (12.5)	9.7 (2.9, 19.0)	42,986 (13.1)	10.1 (3.1, 19.3)	29,851 (13.6)	10.1 (3.2, 19.4)	35,436 (12.6)	9.6 (3.0, 18.9)	21,941 (14.3)	10.3 (3.4, 19.5)

Abbreviations: DC, decompensated cirrhosis; ESLD, end stage liver disease; HCC, hepatocellular carcinoma; IQR, interquartile range; LT, liver transplant; T2DM, type 2 diabetes mellitus.

†Cardiometabolic risk factors include metabolic syndrome, hypertension, dyslipidemia, T2DM, and obesity; ‡Median time-to-event (months)

Supplementary Table 9. Sensitivity analysis - Unadjusted and adjusted risk ratios (RRs) for predictors of disease progression (composite outcome) among patients with MASH/MASLD

Predictor	Unadjusted RR (95% CI)	Adjusted RR (95% CI)†
**Age**		
Age at index	1.02 (1.02, 1.02)	1.01 (1.01, 1.02)
**Gender**		
Male (reference)	-	-
Female	1.05 (1.04, 1.07)	1.03 (1.01, 1.04)
**Race**		
Caucasian (reference)	-	-
African American	1.00 (0.97, 1.03)	0.94 (0.91, 0.97)
Asian	0.67 (0.64, 0.70)	0.77 (0.73, 0.80)
Other/Unknown	0.82 (0.80, 0.84)	0.94 (0.92, 0.96)
**Ethnicity**		
Not Hispanic (reference)	-	-
Hispanic	0.85 (0.83, 0.87)	0.92 (0.90, 0.94)
Unknown	0.88 (0.86, 0.89)	0.97 (0.95, 1.00)
**Region**		
Northeast (reference)	-	-
Midwest	1.05 (1.03, 1.07)	0.98 (0.96, 1.00)
South	1.06 (1.04, 1.08)	0.99 (0.98, 1.01)
West	0.96 (0.94, 0.99)	0.93 (0.91, 0.95)
Other/Unknown	1.10 (1.06, 1.14)	1.06 (1.02, 1.10)
**Comorbidities**		
Weighted Elixhauser index	1.04 (1.04, 1.04)	1.03 (1.03, 1.03)
Hypertension	1.46 (1.43, 1.48)	1.21 (1.19, 1.23)
Dyslipidemia	1.13 (1.11, 1.15)	0.83 (0.82, 0.85)
Obesity	1.03 (1.02, 1.05)	0.92 (0.91, 0.94)
T2DM	1.40 (1.38, 1.42)	1.18 (1.16, 1.19)
CVD	1.74 (1.71, 1.76)	1.23 (1.21, 1.25)
Sleep apnea	1.25 (1.23, 1.27)	1.10 (1.08, 1.11)
Smoking status (current or past)	1.48 (1.46, 1.50)	1.22 (1.20, 1.24)
Thyroid disease	1.20 (1.18, 1.22)	1.06 (1.04, 1.08)
Vitamin D deficiency	1.03 (1.01, 1.04)	0.98 (0.96, 0.99)
End stage renal disease	2.59 (2.42, 2.79)	1.46 (1.36, 1.56)

Abbreviations: CI, confidence interval; CVD, cardiovascular disease; N/A, not applicable; RR, risk ratio; T2DM, type 2 diabetes mellitus.
†Risk ratios adjusted for age, gender, race, ethnicity, region, Elixhauser index, hypertension, dyslipidemia, obesity, T2DM, smoking, CVD, sleep apnea, thyroid disease, vitamin D deficiency, and end-stage renal disease.

Supplementary Table 10. Sensitivity analysis - Healthcare resource utilization and costs during follow-up for MASH/MASLD cohort (n=462,224), stratified by baseline ESLD

Category	With ESLD at baseline (n=76,950)				Without ESLD at baseline (n=385,274)			
	n (%)	Mean (SD)	Median (IQR)	Adjusted Mean (CI)†	n (%)	Mean (SD)	Median (IQR)	Adjusted Mean (CI)†
**Healthcare resource utilization (PPPY)**								
Inpatient admissions	25,454 (33.1)	1.2 (3.2)	0 (0, 1)	0.6 (0.5, 0.6)	40,308 (10.5)	0.4 (1.5)	0 (0, 0)	0.4 (0.4, 0.4)
Emergency department stays	46,354 (60.2)	3.9 (9.4)	1 (0, 4)	2.2 (2.2, 2.3)	144,661 (37.5)	1.9 (4.7)	1 (0, 2)	1.8 (1.7, 1.8)
Outpatient visits	76,258 (99.1)	65 (73.2)	44 (21, 84)	47.8 (47.1, 48.5)	377,343 (97.9)	41.2 (48)	27 (13, 53)	41.6 (41.0, 42.3)
GP visits	75,740 (98.4)	57.4 (67.4)	38 (17, 74)	4.5 (4.4, 4.6)	371,841 (96.5)	36.2 (44.3)	23 (10, 46)	4.0 (3.9, 4.1)
Specialist visits	72,467 (94.2)	137.8 (156.1)	92 (39, 181)	41.9 (41.2, 42.6)	357,244 (92.7)	99.7 (116.5)	66 (27, 130)	36.3 (35.8, 36.9)
Medication dispensations (total)	72,467 (94.2)	39 (32.7)	32 (17, 53)	84.1 (82.6, 85.5)	357,244 (92.7)	29.2 (25)	23 (12, 40)	81.7 (80.4, 83.1)
Medication dispensations (unique)	25,454 (33.1)	1.2 (3.2)	0 (0, 1)	30.2 (29.8, 30.6)	40,308 (10.5)	0.4 (1.5)	0 (0, 0)	28.7 (28.3, 29.0)
**Healthcare costs (PPPY, 2024 USD)**								
Inpatient admissions	28,586 (37.1)	32,669 (89,634)	0 (0, 24,613)	11,000 (10,164, 11,929)	69,950 (18.2)	10,095 (41,824)	0 (0, 0)	6,497 (6,043, 6,999)
Emergency department stays	45,696 (59.4)	4,179 (10,835)	784 (0, 4,309)	1,155 (1,093, 1,215)	168,344 (43.7)	1,724 (5,152.5)	0 (0, 1,632)	843 (798, 891)
Outpatient visits	71,990 (93.6)	28,006 (65,870)	8,276 (1,347, 28,245)	8,156 (7,703, 8,629)	344,876 (89.5)	14,465 (41,985)	3,295 (331, 13,577)	6,710 (6,378, 7,092)
GP visits	32,968 (42.8)	816 (5,518)	0 (0, 134)	208 (181, 237)	141,799 (36.8)	448 (3,605)	0 (0, 27)	179 (156, 204)
Specialist visits	70,832 (92.0)	27,190 (65,168)	7,705 (1,145, 27,124)	8,034 (7,609, 8,508)	335,369 (87.0)	14,016 (41,542)	3,020 (227, 12,968)	6,622 (6,294, 6,999)

Category	With ESLD at baseline (n=76,950)				Without ESLD at baseline (n=385,274)			
	n (%)	Mean (SD)	Median (IQR)	Adjusted Mean (CI)†	n (%)	Mean (SD)	Median (IQR)	Adjusted Mean (CI)†
Medication dispensations (total)‡	73,076 (95.0)	43,491 (139, 713)	9,943 (1,603, 36,898)	21,014 (20,529, 21,472)	366,276 (95.1)	29,524 (106,752)	4,525 (793, 23,724)	13,289 (13,150, 13,433)
Total healthcare cost	76,334 (99.2)	108,345 (201,249)	44,778 (12,861, 122,077)	27,372 (25,975, 28,554)	382,131 (99.2)	55,807 (133,117)	19,090 (4,422, 55,854)	21,489 (20,483, 22,401)

Abbreviations: CI, confidence interval; ESLD, end stage liver disease; IQR, interquartile range; GP, general practitioner; PPPY, per-person-per-year; SD, standard deviation; USD, United States Dollar.

†Multivariable models were adjusted for adjusted for age, gender, race, ethnicity, region, Elixhauser index, hypertension, dyslipidemia, obesity, T2DM, smoking, CVD, sleep apnea, thyroid disease, vitamin D deficiency, end-stage renal disease and prior cost and healthcare resource utilization. ‡For medication dispensations, the full multivariable cost model did not converge because of overdispersion; therefore, adjusted means for this row are based on a model including only the exposure (i.e., history of ESLD at baseline) and not the full set of covariates.

Supplementary Table 11. Sensitivity analysis - Healthcare resource utilization and costs during follow-up for MASH/MASLD cohort without baseline ESLD (n=385,274), stratified by progression status

Category	Progression ESLD (n=57,873)				No progression ESLD (n=327,401)			
	n (%)	Mean (SD)	Median (IQR)	Adjusted Mean (CI)†	n (%)	Mean (SD)	Median (IQR)	Adjusted Mean (CI)†
**Healthcare resource utilization (PPPY)**								
Inpatient admissions	10,235 (17.7)	1.2 (3.1)	0 (0, 1)	0.9 (0.9, 1.0)	30,073 (9.2)	0.2 (0.9)	0 (0, 0)	0.2 (0.2, 0.3)
Emergency department stays	27,790 (48.0)	4 (7.8)	2 (0, 5)	2.9 (2.8, 3.0)	116,871 (35.7)	1.5 (3.9)	0 (0, 2)	1.4 (1.4, 1.5)
Outpatient visits	57,105 (98.7)	69.5 (69.9)	50 (26, 90)	56.7 (55.4, 57.9)	320,238 (97.8)	36.2 (40.9)	24 (12, 47)	37.6 (36.8, 38.4)
GP visits	56,575 (97.8)	61.6 (64.3)	43 (22, 80)	4.9 (4.7, 5.1)	315,266 (96.3)	31.8 (38)	21 (9, 41)	3.5 (3.4, 3.7)
Specialist visits	54,266 (93.8)	142.8 (153.5)	99 (46, 187)	50.4 (49.2, 51.6)	302,978 (92.5)	92.1 (106.8)	61 (25, 121)	32.8 (32.1, 33.6)
Medication dispensations (total)	54,266 (93.8)	41.6 (32.5)	35 (19, 56)	88.4 (86.2, 90.6)	302,978 (92.5)	27 (22.8)	22 (11, 37)	75.3 (73.5, 77.1)
Medication dispensations (unique)	10,235 (17.7)	1.2 (3.1)	0 (0, 1)	31.5 (30.9, 32.1)	30,073 (9.2)	0.2 (0.9)	0 (0, 0)	26.0 (25.5, 26.5)
**Healthcare costs (PPPY, 2024 USD)**								
Inpatient admissions	24,783 (42.8)	36,703 (86,816)	0 (0, 33,997)	18,507 (16,912, 20,263)	45,167 (13.8)	5,391 (24,061)	0 (0, 0)	3,308 (2,987, 3,633)
Emergency department stays	37,864 (65.4)	4,306 (8,996)	1,355 (0, 5,076)	1,648 (1,561, 1,740)	130,480 (39.9)	1,267 (3,943)	0 (0, 1185)	619 (581, 659)
Outpatient visits	55,411 (95.7)	30,534 (69,462)	10,534 (2,306, 30,919)	11,326 (10,385, 12,210)	289,465 (88.4)	11,624 (34,172)	2,622 (209, 11,149)	5,826 (5,348, 6,309)
GP visits	25,515 (44.1)	829 (5,632)	0 (0, 151)	262 (210, 424)	116,284 (35.5)	381 (3,108)	0 (0, 14)	172 (137, 273)
Specialist visits	54,752 (94.6)	29,705 (68,812)	9,947 (2,081, 29,727)	11,194 (10,267, 12,069)	280,617 (85.7)	11,243 (33,802)	2,377 (132, 10,574)	5,730 (5,273, 6,198)

Category	Progression ESLD (n=57,873)				No progression ESLD (n=327,401)			
	n (%)	Mean (SD)	Median (IQR)	Adjusted Mean (CI)†	n (%)	Mean (SD)	Median (IQR)	Adjusted Mean (CI)†
Medication dispensations (total)‡	55,620 (96.1)	43,979 (149,736)	10,126 (1,853, 37,223)	8,471 (8,458, 8,485)	310,656 (94.9)	26,969 (96,973)	3,858 (699, 21,485)	6,301 (6,301, 6,301)
Total healthcare cost	57,749 (99.8)	115,521 (206,512)	52,190 (16,904, 135,065)	36,498 (34,444, 38,358)	324,382 (99.1)	45,251 (112,126)	15,842 (3,647, 46,239)	16,306 (15,438, 17,027)

Abbreviations: CI, confidence interval; ESLD, end stage liver disease; IQR, interquartile range; GP, general practitioner; PPPY, per-person-per-year; SD, standard deviation; USD, United States Dollars

†Multivariable models were adjusted for adjusted for age, gender, race, ethnicity, region, Elixhauser index, hypertension, dyslipidemia, obesity, T2DM, smoking, CVD, sleep apnea, thyroid disease, vitamin D deficiency, end-stage renal disease and prior cost and healthcare resource utilization. ‡For medication dispensations, the full multivariable cost model did not converge because of overdispersion; therefore, adjusted means for this row are based on a model including only the exposure (i.e., ESLD progression) and not the full set of covariates.

Supplementary Table 12. Sensitivity analysis - Unadjusted and adjusted risk ratios (RRs) for predictors of disease progression (composite outcome) after reclassifying progression events within the first 30 days as baseline ESLD

Predictor	Primary analysis results		Sensitivity analysis results	
	Unadjusted RR (95% CI)	Adjusted RR (95% CI)†	Unadjusted RR (95% CI)	Adjusted RR (95% CI)†
**Age**				
Age at index	1.02 (1.02, 1.03)	1.02 (1.02, 1.02)	1.03 (1.02, 1.03)	1.02 (1.02, 1.02)
**Gender**				
Male (reference)	-	-	-	-
Female	1.13 (1.07, 1.18)	1.02 (0.97, 1.08)	1.15 (1.09, 1.21)	1.04 (0.98, 1.10)
**Race**				
Caucasian (reference)	-	-	-	-
African American	0.86 (0.77, 0.97)	0.83 (0.74, 0.94)	0.87 (0.77, 0.99)	0.84 (0.74, 0.96)
Asian	0.75 (0.66, 0.86)	0.90 (0.79, 1.03)	0.77 (0.67, 0.89)	0.93 (0.81, 1.07)
Other/Unknown	0.82 (0.77, 0.89)	0.96 (0.89, 1.08)	0.82 (0.76, 0.89)	0.97 (0.89, 1.05)
**Ethnicity**				
Not Hispanic (reference)	-	-	-	-
Hispanic	0.87 (0.81, 0.94)	0.96 (0.88, 1.03)	0.87 (0.80, 0.94)	0.96 (0.88, 1.04)
Unknown	0.87 (0.81, 0.94)	0.96 (0.88, 1.04)	0.87 (0.80, 0.94)	0.96 (0.88, 1.05)
**Region**				
Northeast (reference)	-	-	-	-
Midwest	1.17 (1.10, 1.25)	1.07 (1.00, 1.14)	1.15 (1.08, 1.24)	1.06 (0.99, 1.13)
South	1.14 (1.07, 1.22)	1.07 (1.00, 1.14)	1.10 (1.02, 1.18)	1.03 (0.96, 1.11)
West	0.99 (0.91, 1.08)	0.94 (0.86, 1.02)	0.97 (0.89, 1.07)	0.92 (0.83, 1.00)
Other/Unknown	1.18 (1.05, 1.33)	1.15 (1.03, 1.29)	1.18 (1.04, 1.33)	1.15 (1.02, 1.29)
**Comorbidities**				
Weighted Elixhauser index	1.03 (1.03, 1.04)	1.02 (1.02, 1.03)	1.03 (1.03, 1.04)	1.02 (1.02, 1.03)

Predictor	Primary analysis results		Sensitivity analysis results	
	**Unadjusted RR (95% CI)**	**Adjusted RR (95% CI)†**	**Unadjusted RR (95% CI)**	**Adjusted RR (95% CI)†**
Hypertension	1.50 (1.42, 1.58)	1.18 (1.12, 1.26)	1.53 (1.45, 1.61)	1.19 (1.12, 1.26)
Dyslipidemia	1.13 (1.07, 1.19)	0.80 (0.76, 0.85)	1.16 (1.09, 1.23)	0.81 (0.76, 0.87)
Obesity	1.07 (1.02, 1.13)	0.97 (0.92, 1.02)	1.06 (1.01, 1.12)	0.96 (0.91, 1.02)
T2DM	1.51 (1.44, 1.58)	1.25 (1.19, 1.32)	1.54 (1.46, 1.62)	1.26 (1.20, 1.34)
CVD	1.70 (1.63, 1.79)	1.18 (1.12, 1.24)	1.72 (1.64, 1.81)	1.17 (1.11, 1.24)
Sleep apnea	1.30 (1.24, 1.37)	1.13 (1.07, 1.19)	1.32 (1.25, 1.39)	1.15 (1.09, 1.21)
Smoking status (current or past)	1.51 (1.43, 1.60)	1.23 (1.16, 1.30)	1.52 (1.43, 1.61)	1.23 (1.16, 1.30)
Thyroid disease	1.27 (1.21, 1.34)	1.11 (1.05, 1.17)	1.28 (1.21, 1.36)	1.11 (1.05, 1.17)
Vitamin D deficiency	1.08 (1.03, 1.14)	1.03 (0.98, 1.08)	1.08 (1.03, 1.14)	1.03 (0.97, 1.09)
End stage renal disease	2.21 (1.69, 2.88)	1.21 (0.94, 1.56)	2.32 (1.75, 3.07)	1.27 (0.97, 1.65)

Abbreviations: CI, confidence interval; CVD, cardiovascular disease; N/A, not applicable; RR, risk ratio; T2DM, type 2 diabetes mellitus.

†Risk ratios adjusted for age, gender, race, ethnicity, region, Elixhauser index, hypertension, dyslipidemia, obesity, T2DM, smoking, CVD, sleep apnea, thyroid disease, vitamin D deficiency, and end-stage renal disease.

Supplementary Table 13. Sensitivity analysis of follow-up healthcare resource utilization and costs in the MASH cohort (n=49,983), by baseline ESLD status, after reclassifying progression events within 30 days as baseline ESLD

Category	With ESLD at baseline (n=16,922)				Without ESLD at baseline (n=33,061)			
	n (%)	Mean (SD)	Median (IQR)	Adjusted Mean (CI)†	n (%)	Mean (SD)	Median (IQR)	Adjusted Mean (CI)†
**Healthcare resource utilization (PPPY)**								
Inpatient admissions	5,019 (29.7)	1.2 (3.1)	0 (0, 1)	0.6 (0.5, 0.7)	2,659 (8.0)	0.3 (1.5)	0 (0, 0)	0.3 (0.3, 0.3)
ED	9,063 (53.6)	3.2 (6.5)	1 (0, 4)	2.2 (2.0, 2.4)	9,692 (29.3)	1.6 (4.3)	0 (0, 2)	1.5 (1.4, 1.6)
Outpatient visits	16,778 (99.1)	66.6 (72.4)	47 (23, 86)	55.0 (53.1, 57.1)	32,432 (98.1)	42.3 (48.4)	28 (13, 54)	44.8 43.2, 46.4)
GP visits	16,665 (98.5)	58.6 (66.1)	40 (19, 76)	5.3 (4.9, 5.7)	31,981 (96.7)	37.4 (44.9)	24 (11, 48)	4.3 (4.0, 4.7)
Specialist visits	15,859 (93.7)	144.2 (153.8)	102 (44, 192)	47.6 (45.8, 49.5)	30,780 (93.1)	108.9 (123.1)	74 (32, 143)	38.7 (37.2, 40.2)
Medication dispensations (total)	15,859 (93.7)	39.4 (31.7)	33 (18, 54)	93.9 (90.2, 97.7)	30,780 (93.1)	30.2 (25.3)	24 (13, 41)	88.1 (84.7, 91.6)
Medication dispensations (unique)	5,019 (29.7)	1.2 (3.1)	0 (0, 1)	32.3 (31.3, 33.4)	2,659 (8.0)	0.3 (1.5)	0 (0, 0)	29.4 (28.5, 30.4)
**Healthcare costs (PPPY, 2024 USD)**								
Inpatient admissions	6,534 (38.6)	33,003 (83,015)	0 (0, 27,786)	11,757 (9,682, 13,680)	5,033 (15.2)	8,696 (40,378)	0 (0, 0)	4,459 (3,637, 5,219)
ED stays	11,049 (65.3)	4,936 (10,911)	1,437 (0, 5,601)	1,846 (1,613, 2,104)	14,940 (45.2)	1,923 (5,322)	0 (0, 1,835)	1,044 (905, 1,203)
Outpatient visits	16,785 (99.2)	44,109 (70,094)	22,640 (8,806, 51,909)	16,004 (14,636, 17,386)	32,665 (98.8)	23,260 (43,788)	10,425 (3,978, 25,596)	10,986 (10,045 11,963)
GP visits	11,174 (66.0)	2,054 (5,929)	425 (0, 1,994)	786 (682, 887)	19,346 (58.5)	1,163 (4,320)	180 (0, 1,135)	617 (539, 697)
Specialist visits	16,704 (98.7)	42,055 (68,973)	20,964 (7,827, 48,845)	15,417 (14,067, 16,807)	32,443 (98.1)	22,098 (42,939)	9,533 (3,464, 24,310)	10,517 (9,599, 11,503)

Category	With ESLD at baseline (n=16,922)				Without ESLD at baseline (n=33,061)			
	n (%)	Mean (SD)	Median (IQR)	Adjusted Mean (CI)†	n (%)	Mean (SD)	Median (IQR)	Adjusted Mean (CI)†
Medication dispensations (total)‡	15,993 (94.5)	51,588 (164,961)	15,053 (2,421, 50,085)	25,215 (24,090, 26,445)	31,458 (95.2)	35,712 (102,979)	7,021 (1,094, 32,103)	15,622 (15,154, 16,118)
Total healthcare cost	16,900 (99.9)	133,635 (220,659)	70,659 (27,268, 162,886)	41,118 (37,384, 45,086)	33,016 (99.9)	69,592 (132,366)	29,854 (10,013, 74,751)	27,336 (25,002, 29,964)

Abbreviations: CI, confidence interval; ED, emergency department; ESLD, end stage liver disease; IQR, interquartile range; GP, general practitioner; PPPY, per-person-per-year; SD, standard deviation; USD, United States Dollar.
†Multivariable models were adjusted for adjusted for age, gender, race, ethnicity, region, Elixhauser index, hypertension, dyslipidemia, obesity, T2DM, smoking, CVD, sleep apnea, thyroid disease, vitamin D deficiency, end-stage renal disease and prior cost and healthcare resource utilization. ‡For medication dispensations, the full multivariable cost model did not converge because of overdispersion; therefore, adjusted means for this row are based on a model including only the exposure (i.e., history of ESLD at baseline) and not the full set of covariates.

Supplementary Table 14. Sensitivity analysis of follow-up healthcare resource utilization and costs in the MASH cohort without baseline ESLD, stratified by progression status, after reclassifying progression events within 30 days as baseline ESLD

Category	Progression ESLD (n=4,808)				No progression ESLD (n=28,253)			
	n (%)	Mean (SD)	Median (IQR)	Adjusted Mean (CI)†	n (%)	Mean (SD)	Median (IQR)	Adjusted Mean (CI)†
**Healthcare resource utilization (PPPY)**								
Inpatient admissions	634 (13.2)	1.1 (3.0)	0 (0, 1)	0.7 (0.6, 0.9)	2,025 (7.2)	0.2 (0.9)	0 (0, 0)	0.2 (0.1, 0.2)
ED	1,824 (37.9)	3.4 (7.1)	2 (0, 4)	2.4 (2.0, 2.8)	7,868 (27.8)	1.2 (3.5)	0 (0, 1)	1.1 (1.0, 1.3)
Outpatient visits	4,754 (98.9)	72.8 (72.5)	52 (28, 93)	58.6 (54.4, 63.2)	27,678 (98.0)	37.1 (40.8)	25 (12, 48)	40.7 (37.9, 43.8)
GP visits	4,715 (98.1)	64.4 (66.8)	45 (24, 82)	4.6 (3.9, 5.4)	27,266 (96.5)	32.8 (38.1)	22 (10, 42)	3.2 (2.7, 3.8)
Specialist visits	4,528 (94.2)	158.4 (159.0)	115 (57, 212)	52.6 (48.5, 57.0)	26,252 (92.9)	100.5 (113.8)	68 (30, 132)	36.0 (33.3, 38.9)
Medication dispensations (total)	4,528 (94.2)	43.5 (32.5)	37 (22, 58)	89.2 (81.8, 97.2)	26,252 (92.9)	27.9 (23.2)	23 (12, 38)	78.3 (72.1, 85.2)
Medication dispensations (unique)	634 (13.2)	1.1 (3.0)	0 (0, 1)	29.7 (27.8, 31.8)	2,025 (7.2)	0.2 (0.9)	0 (0, 0)	25.6 (24.0, 27.3)
**Healthcare costs (PPPY, 2024 USD)**								
Inpatient admissions	1,808 (37.6)	33,046 (84,051)	0 (0, 25,678)	11,800 (8,232, 15,493)	3,225 (11.4)	4,552 (24,244)	0 (0, 0)	2,147 (1,410, 2,969)
ED stays	3,278 (68.2)	5,098 (10,123)	1,713 (0, 5,941)	1,791 (1,497, 2,076)	11,662 (41.3)	1,382 (3,702)	0 (0, 1,354)	720 (569, 867)
Outpatient visits	4.790 (99.6)	48,158 (77,353)	25,450 (11,494, 56,071)	16,239 (12,969, 19,899)	27,875 (98.7)	19,024 (33,199)	8,911 (3,507, 21,301)	9,030 (7,257, 10,962)
GP visits	3,256 (67.7)	2,173 (6,970)	487 (0, 2,186)	706 (560, 860)	16,090 (56.9)	991 (3,657)	152 (0, 1,010)	485 (387, 590)
Specialist visits	4,776 (99.3)	45,985 (76,059)	23,960 (10,239, 53,001)	15,662 (12,398, 19,297)	27,667 (97.9)	18,033 (32,551)	8,130 (2,954, 20,042)	8,624 (6,869, 10,558)

Category	Progression ESLD (n=4,808)				No progression ESLD (n=28,253)			
	n (%)	Mean (SD)	Median (IQR)	Adjusted Mean (CI)†	n (%)	Mean (SD)	Median (IQR)	Adjusted Mean (CI)†
Medication dispensations (total)‡	4,631 (96.3)	53,904 (127,078)	16,108 (3,024, 53,165)	21,849 (20,379, 23,305)	26,827 (95.0)	32,617 (97,959)	5,896 (952, 28,943)	14,615 (14,122, 15,083)
Total healthcare cost	4,808 (100.0)	140,205 (194.921)	74,870 (30,607, 167,543)	37,728 (32,235, 43,956)	28,208 (99.8)	57,575 (114,214)	25,587 (8,616, 63,020)	19,782 (16,861, 22,673)

Abbreviations: CI, confidence interval; ESLD, end stage liver disease; IQR, interquartile range; GP, general practitioner; PPPY, per-person-per-year; SD, standard deviation; USD, United States Dollar.

†Multivariable models were adjusted for adjusted for age, gender, race, ethnicity, region, Elixhauser index, hypertension, dyslipidemia, obesity, T2DM, smoking, CVD, sleep apnea, thyroid disease, vitamin D deficiency, end-stage renal disease and prior cost and healthcare resource utilization. ‡For medication dispensations, the full multivariable cost model did not converge because of overdispersion; therefore, adjusted means for this row are based on a model including only the exposure (i.e., ESLD progression) and not the full set of covariates.

SUPPLEMENTARY METHODS

Information on data source:

Optum's de-identified Market Clarity Data (Optum® Market Clarity) is an integrated, multi-source medical claims, pharmacy claims, and electronic health records data set. Optum® Market Clarity links electronic health record data - including lab results, vital signs and measurements, diagnoses, procedures and information derived from unstructured clinical notes using natural language processing - with historical, linked administrative claim data - including pharmacy claims, physician claims, clinical information facility claims and medications prescribed and administered. Optum® Market Clarity is statistically de-identified under the HIPAA Privacy Rule's Expert Determination method and managed according to Optum® customer data use agreements.

The RECORD checklist

	Item number	STROBE items	RECORD items	Location in manuscript
**Title and abstract**				
	1	(a) Indicate the study's design with a commonly used term in the title or the abstract. (b) Provide in the abstract an informative and balanced summary of what was done and what was found.	RECORD 1.1: The type of data used should be specified in the title or abstract. When possible, the name of the databases used should be included. RECORD 1.2: If applicable, the geographic region and time frame within which the study took place should be reported in the title or abstract. RECORD 1.3: If linkage between databases was conducted for the study, this should be clearly stated in the title or abstract.	Title, Abstract
**Introduction**				
Background rationale	2	Explain the scientific background and rationale for the investigation being reported.		Introduction
Objectives	3	State specific objectives, including any prespecified hypotheses.		Introduction
**Methods**				
Study design	4	Present key elements of study design early in the paper.		Methods
Setting	5	Describe the setting, locations, and relevant dates, including periods of recruitment, exposure, follow-up, and data collection.		Methods, Supplementary Methods
Participants	6	(a) Cohort study: Give the eligibility criteria and the sources and methods of selection of participants. Describe	RECORD 6.1: The methods of study population selection (such as codes or algorithms used to	Methods, Supplementary Table 1 and 2

		methods of follow-up. Case-control study: Give the eligibility criteria and the sources and methods of case ascertainment and control selection. Give the rationale for the choice of cases and controls. Cross-sectional study: Give the eligibility criteria and the sources and methods of selection of participants. (b) Cohort study: For matched studies, give matching criteria and number of exposed and unexposed. Case-control study: For matched studies, give matching criteria and the number of controls per case.	identify subjects) should be listed in detail. If this is not possible, an explanation should be provided. RECORD 6.2: Any validation studies of the codes or algorithms used to select the population should be referenced. If validation was conducted for this study and not published elsewhere, detailed methods and results should be provided. RECORD 6.3: If the study involved linkage of databases, consider use of a flow diagram or other graphical display to demonstrate the data linkage process, including the number of individuals with linked data at each stage.	
Variables	7	Clearly define all outcomes, exposures, predictors, potential confounders, and effect modifiers. Give diagnostic criteria, if applicable.	RECORD 7.1: A complete list of codes and algorithms used to classify exposures, outcomes, confounders, and effect modifiers should be provided. If these cannot be reported, an explanation should be provided.	Methods, Supplementary Table 2
Data sources/ measurement	8	For each variable of interest, give sources of data and details of methods of assessment (measurement). Describe comparability of assessment methods if there is more than one group.		Methods
Bias	9	Describe any efforts to address potential sources of bias.		Methods & Results (Sensitivity Analyses)
Study size	10	Explain how the study size was arrived at.		Methods, Figure 1

Quantitative methods	11	Explain how quantitative variables were handled in the analyses. If applicable, describe which groupings were chosen and why.		Methods, Supplementary Table 2
Statistical methods	12	(a) Describe all statistical methods, including those used to control for confounding. (b) Describe any methods used to examine subgroups and interactions. (c) Explain how missing data were addressed. (d) Cohort study: If applicable, explain how loss to follow-up was addressed. Case-control study: If applicable, explain how matching of cases and controls was addressed. Cross-sectional study: If applicable, describe analytical methods taking account of sampling strategy. (e) Describe any sensitivity analyses.		Methods (Statistical Analysis)
Data access and cleaning methods		N/A	RECORD 12.1: Authors should describe the extent to which the investigators had access to the database population used to create the study population. RECORD 12.2: Authors should provide information on the data cleaning methods used in the study.	Methods, Figure 1
Linkage		N/A	RECORD 12.3: State whether the study included person-level, institutional-level, or other data linkage across two or more databases. The methods of linkage and methods of linkage	Methods, Supplementary Methods

			quality evaluation should be provided.	
**Results**				
Participants	13	(a) Report the numbers of individuals at each stage of the study (e.g., numbers potentially eligible, examined for eligibility, confirmed eligible, included in the study, completing follow-up, and analysed). (b) Give reasons for nonparticipation at each stage. (c) Consider use of a flow diagram.	RECORD 13.1: Describe in detail the selection of the persons included in the study (i.e., study population selection), including filtering based on data quality, data availability, and linkage. The selection of included persons can be described in the text and/or by means of the study flow diagram.	Figure 1
Descriptive data	14	(a) Give characteristics of study participants (e.g., demographic, clinical, and social) and information on exposures and potential confounders. (b) Indicate the number of participants with missing data for each variable of interest. (c) Cohort study: summarise follow-up time (e.g., average and total amount).		Results, Table 1
Outcome data	15	Cohort study: Report numbers of outcome events or summary measures over time. Case-control study: Report numbers in each exposure category or summary measures of exposure. Cross-sectional study: Report numbers of outcome events or summary measures.		Results, Figure 1, Table 1
Main results	16	(a) Give unadjusted estimates and, if applicable, confounder-adjusted estimates and their precision (e.g., 95% confidence interval). Make clear which confounders were adjusted for and		Results and Tables

		why they were included. (b) Report category boundaries when continuous variables were categorized. (c) If relevant, consider translating estimates of relative risk into absolute risk for a meaningful time period.		
Other analyses	17	Report other analyses done—e.g., analyses of subgroups and interactions and sensitivity analyses.		Methods & Results
**Discussion**				
Key results	18	Summarise key results with reference to study objectives.		Discussion
Limitations	19	Discuss limitations of the study, taking into account sources of potential bias or imprecision. Discuss both direction and magnitude of any potential bias.	RECORD 19.1: Discuss the implications of using data that were not created or collected to answer the specific research question(s). Include discussion of misclassification bias, unmeasured confounding, missing data, and changing eligibility over time, as they pertain to the study being reported.	Discussion
Interpretation	20	Give a cautious overall interpretation of results considering objectives, limitations, multiplicity of analyses, results from similar studies, and other relevant evidence.		Discussion
Generalisability	21	Discuss the generalisability (external validity) of the study results.		Discussion
**Other information**				
Funding	22	Give the source of funding and the role of the funders for the present study and, if		Title (under “Declarations”)

		applicable, for the original study on which the present article is based.		
Accessibility of protocol, raw data, and programming code		N/A	RECORD 22.1: Authors should provide information on how to access any supplemental information such as the study protocol, raw data, or programming code.	Title (under "Availability of data and material")

Abbreviations: N/A, not applicable; RECORD, REporting of studies Conducted using Observational Routinely collected health Data; STROBE, Strengthening the Reporting of Observational Studies in Epidemiology.

SUPPLEMENTARY RESULTS

MASLD Sensitivity Analysis – Additional results

Testing patterns in the MASLD cohort paralleled the primary MASH analysis (**Supplementary Table 7**): patients with baseline ESLD consistently had higher use of NITs, imaging tests, liver biopsy, and liver function tests than those without baseline ESLD. Testing in both groups increased from the diagnosis window (days 0,30) to follow-up (day 31+). Transient elastography and MRI were the most commonly used NIT and imaging tests, while MRE, LiverMultiScan, and FibroSure/Fibrotest were rare. Absolute rates of NITs, imaging tests, and liver biopsy during follow-up were modestly lower in the MASH cohort, but the direction of differences was unchanged.

Among patients without baseline ESLD, progression patterns in the MASLD cohort were directionally consistent with the MASH cohort (**Supplementary Table 8**), with DC as the most common first event and HCC and LT remaining rare. Composite ESLD progression was modestly higher in MASLD than in MASH (12.5% vs 10.7%) and occurred slightly sooner (median ~9–10 months vs ~10–11 months). High-risk subgroups (≥2 or ≥3 cardiometabolic risk factors, obesity, T2DM) showed the same gradient in both cohorts, with the highest progression proportions among those with T2DM.

References

1. Wong RJ, Kachru N, Martinez DJ, Moynihan M, Ozbay AB, Gordon SC. Real-world Comorbidity Burden, Health Care Utilization, and Costs of Nonalcoholic Steatohepatitis Patients With Advanced Liver Diseases. *Journal of clinical gastroenterology.* 2021;55(10):891-902.
2. Grønbæk L, Vilstrup H, Pedersen L, Christensen K, Jepsen P. Family occurrence of autoimmune hepatitis: A Danish nationwide registry-based cohort study. *Journal of hepatology.* 2018;69(4):873-877.
3. Kramer JR, Hartman C, White DL, et al. Validation of HIV-infected cohort identification using automated clinical data in the Department of Veterans Affairs. *HIV medicine.* 2019;20(8):567-570.
4. Han G, Turcotte K, Jivani K, Babul S, Pike I. *Poisonings in British Columbia, 2000 - 2005.* 2009.
5. Tapper EB, Bonafede M, Fishman J, et al. Healthcare resource utilization and costs of care in the United States for patients with non-alcoholic steatohepatitis. *Journal of medical economics.* 2023;26(1):348-356.
6. Allen AM, Van Houten HK, Sangaralingham LR, Talwalkar JA, McCoy RG. Healthcare Cost and Utilization in Nonalcoholic Fatty Liver Disease: Real-World Data From a Large U.S. Claims Database. *Hepatology.* 2018;68(6):2230-2238.
7. Pepin KM, Welle CL, Guglielmo FF, Dillman JR, Venkatesh SK. Magnetic resonance elastography of the liver: everything you need to know to get started. *Abdominal radiology (New York).* 2022;47(1):94-114.
8. Perspectum. LiverMultiScan. 2024; https://www.perspectum.com/our-products/livermultiscan-rollback-content-21apr23. Accessed July 16, 2024.
9. Research Data Assistance Center. How to Identify Hospital Claims for Emergency Room Visits in the Medicare Claims Data. 2015; https://resdac.org/articles/how-identify-hospital-claims-emergency-room-visits-medicare-claims-data.
10. Powell WR, Kaiksow FA, Kind AJH, Sheehy AM. What Is an Observation Stay? Evaluating the Use of Hospital Observation Stays in Medicare. *Journal of the American Geriatrics Society.* 2020;68(7):1568-1572.
11. Research Data Assistance Center. Identifying Observation Stays in the Medicare Fee-For-Service Claims Data. 2020; https://resdac.org/articles/identifying-observation-stays-medicare-fee-service-claims-data.
12. Sheehy AM, Shi F, Kind AJH. Identifying Observation Stays In Medicare Data: Policy Implications of a Definition. *Journal of hospital medicine.* 2019;14(2):96-100.
13. Alberti KGMM, Eckel RH, Grundy SM, et al. Harmonizing the Metabolic Syndrome. *Circulation.* 2009;120(16):1640-1645.
14. Alberti KGMM, Zimmet P, Shaw J. The metabolic syndrome - a new worldwide definition. *The Lancet.* 2005;366(9491):1059-1062.
15. Song S, Lee SE, Oh SK, et al. Demographics, treatment trends, and survival rate in incident pulmonary artery hypertension in Korea: A nationwide study based on the health insurance review and assessment service database. *PloS one.* 2018;13(12):e0209148.
16. Quan H, Khan N, Hemmelgarn BR, et al. Validation of a Case Definition to Define Hypertension Using Administrative Data. *Hypertension.* 2009;54(6):1423-1428.
17. Unger T, Borghi C, Charchar F, et al. 2020 International Society of Hypertension Global Hypertension Practice Guidelines. *Hypertension.* 2020;75(6):1334-1357.
18. Gabriela Dieguez F, Bruce Pyenson F, Tomicki S, Charles Steffens F. Obesity in A Claims-Based Analysis of the Commercially Insured Population: Prevalence, Cost, and the

Influence of Obesity Services and Anti-Obesity Medication Coverage on Health Expenditures - MILLIMAN REPORT. 2021.
19. Mesregah MK, Mgbam P, Fresquez Z, Wang JC, Buser Z. Impact of chronic hyperlipidemia on perioperative complications in patients undergoing lumbar fusion: a propensity score matching analysis. *European Spine Journal.* 2022;31(10):2579-2586.
20. Oake J, Aref-Eshghi E, Godwin M, et al. Using Electronic Medical Record to Identify Patients With Dyslipidemia in Primary Care Settings: International Classification of Disease Code Matters From One Region to a National Database. *Biomedical Informatics Insights.* 2017;9:1178222616685880.
21. Ramasamy A, Laliberté F, Aktavoukian SA, et al. Direct and Indirect Cost of Obesity Among the Privately Insured in the United States: A Focus on the Impact by Type of Industry. *Journal of occupational and environmental medicine.* 2019;61(11):877-886.
22. Suissa K, Schneeweiss S, Lin KJ, Brill G, Kim SC, Patorno E. Validation of obesity-related diagnosis codes in claims data. *Diabetes, obesity & metabolism.* 2021;23(12):2623-2631.
23. Chi GC, Li X, Tartof SY, Slezak JM, Koebnick C, Lawrence JM. Validity of ICD-10-CM codes for determination of diabetes type for persons with youth-onset type 1 and type 2 diabetes. *BMJ open diabetes research & care.* 2019;7(1):e000547.
24. Sacks DB, Arnold M, Bakris GL, et al. Guidelines and Recommendations for Laboratory Analysis in the Diagnosis and Management of Diabetes Mellitus. *Clinical Chemistry.* 2023;69(8):808-868.
25. Military Health System. *ANEMIA; IRON DEFICIENCY.* 2015.
26. Collaboration GBoCD. The Burden of Cardiovascular Diseases Among US States, 1990-2016. *JAMA Cardiology.* 2018;3(5):375-389.
27. Mabeza RM, Mao Y, Maynard K, Lee C, Benharash P, Yetasook A. Bariatric surgery outcomes in geriatric patients: a contemporary, nationwide analysis. *Surgery for Obesity and Related Diseases.* 2022;18(8):1005-1011.
28. Jolley RJ, Liang Z, Peng M, et al. Identifying Cases of Sleep Disorders through International Classification of Diseases (ICD) Codes in Administrative Data. *International journal of population data science.* 2018;3(1):448.
29. Havard A, Jorm LR, Lujic S. Risk adjustment for smoking identified through tobacco use diagnoses in hospital data: a validation study. *PloS one.* 2014;9(4):e95029.
30. Song YS, Kim KS, Kim SK, Cho YW, Choi HG. Screening Leads to Overestimated Associations of Thyroid Dysfunction and Thyroiditis with Thyroid Cancer Risk. *Cancers.* 2021;13(21).
31. Choi R, Cho SE, Lee SG, Lee EH. Recent Information on Vitamin D Deficiency in an Adult Korean Population Visiting Local Clinics and Hospitals. *Nutrients.* 2022;14(9).
32. Yu O, Christ JP, Schulze-Rath R, et al. Incidence, prevalence, and trends in polycystic ovary syndrome diagnosis: a United States population-based study from 2006 to 2019. *American Journal of Obstetrics and Gynecology.* 2023;229(1):39.e31-39.e12.
33. Pham ANQ, Cummings M, Yuksel N, et al. Development and validation of a case definition for problematic menopause in primary care electronic medical records. *BMC Medical Informatics and Decision Making.* 2023;23(1):202.
34. Friberg L, Gasparini A, Carrero JJ. A scheme based on ICD-10 diagnoses and drug prescriptions to stage chronic kidney disease severity in healthcare administrative records. *Clinical kidney journal.* 2018;11(2):254-258.
35. Gibertoni D, Voci C, Iommi M, et al. Developing and validating an algorithm to identify incident chronic dialysis patients using administrative data. *BMC Medical Informatics and Decision Making.* 2020;20(1):185.
36. Pooler BD, Hernando D, Reeder SB. Clinical Implementation of a Focused MRI Protocol for Hepatic Fat and Iron Quantification. *AJR American journal of roentgenology.* 2019;213(1):90-95.
