# Supplementary material for: Progression of Metabolic Dysfunction–Associated Steatohepatitis in US Adults Using Linked Records and Claims
Source: Gastro Hep Adv. 2026 Jun 10;5(9):101031. doi: 10.1016/j.gastha.2026.101031 (PMC13351128; doi:10.1016/j.gastha.2026.101031)
Supplement: Extended PDF [file mmc2.pdf]

Gastro Hep Advances 2026;5:101031

ORIGINAL RESEARCH—CLINICAL

Progression of Metabolic Dysfunction–Associated Steatohepatitis in US Adults Using Linked Records and Claims

Yestle Kim,[1] Romina Fakhraei,[2] John C. O'Donnell,[1] Karissa Johnston,[2] Melissa Bather,[2] Reem Mustafa,[2] Andrew R. Kennedy,[2] and Amreen Dinani[3]

[1]Madrigal Pharmaceuticals, Inc, West Conshohocken, Pennsylvania; [2]Broadstreet HEOR, Vancouver, British Columbia, Canada; and [3]Duke University Health System, Durham, North Carolina

BACKGROUND AND AIMS: Metabolic dysfunction–associated steatohepatitis (MASH) affects approximately 5% of adults globally and, without effective treatment, may progress to end-stage liver disease (ESLD), including compensated cirrhosis, decompensated cirrhosis (DC), hepatocellular carcinoma, and liver transplantation. Using real-world data, this study characterized the natural history and health-care impact of MASH among US adults. **METHODS:** A retrospective cohort study was conducted using Optum's deidentified Market Clarity Data (January 2021–March 2024). Noninvasive test (NIT) use at diagnosis (days 0–30) and during follow-up was described. Predictors of progression were estimated with modified Poisson regression, time to first ESLD was assessed with Cox models, and health-care resource utilization (HCRU) and costs with multivariable regression. **RESULTS:** Among 49,983 patients with MASH, 16,359 (32.7%) had ESLD at baseline (compensated cirrhosis 3965; DC 11,406; hepatocellular carcinoma 514; liver transplantation 474). Of the 33,624 without baseline ESLD, 15.9% progressed; median time to first ESLD event was 10.6 months (interquartile range: 3.8–19.7), with DC the most frequent first event. Around diagnosis, 3771 patients (7.5%) underwent ≥1 NIT or imaging test, slightly higher with baseline ESLD than without (8.1% vs 7.3%); NIT/imaging use increased during follow-up. Older age, hypertension, type 2 diabetes, cardiovascular disease, sleep apnea, smoking, and thyroid disease were associated with higher progression risk. Patients who progressed had higher HCRU and costs than those who did not progress. **CONCLUSION:** In this large US cohort, progression from MASH to ESLD was associated with high HCRU and costs, particularly among patients with ESLD or subsequent progression. These findings highlight the need for earlier risk stratification and targeted care to mitigate burden.

Keywords: Metabolic Dysfunction–Associated Steatohepatitis; Cirrhosis; Progression; Health-Care Resource Use

Introduction

Metabolic dysfunction-associated steatotic liver disease (MASLD) is the most common chronic liver disease worldwide, affecting approximately 30% of adult and is projected to exceed 55% by 2040.[1,2] It is defined as hepatic steatosis with at least 1 cardiometabolic risk factor, and is strongly linked to obesity, insulin resistance, and type 2 diabetes mellitus (T2DM).[2,3] Metabolic dysfunction–associated steatohepatitis (MASH) is the progressive form of MASLD, characterized by hepatic inflammation and fibrosis.[4,5] Approximately 5% of adults are affected globally, with an estimated 10%–30% of patients with MASLD advancing to MASH.[3–5] Recent data in middle-aged Americans reports a 14% prevalence of MASH, suggesting that the true prevalence may be higher.[6] Without effective treatment and proper risk stratification, MASH may progress to end-stage liver disease (ESLD), including compensated cirrhosis (CC), decompensated cirrhosis (DC), hepatocellular carcinoma (HCC), and liver failure. Untreated MASH is also associated with elevated cardiovascular disease (CVD) risk and all-cause mortality.[7–10]

Beyond clinical sequelae, MASH is linked to reduced health-related quality of life and substantial economic burden, with health-care resource utilization (HCRU) and costs rising sharply with advanced fibrosis and multimorbidity.[7,11,12] Historically, MASH management has focused on lifestyle interventions such as diet, exercise, and weight loss. More recently, therapeutic advances have been made with the conditional US Food and Drug Administration approvals of semaglutide (Wegovy), a glucagon-like peptide-1 receptor agonist, and resmetirom (Rezdiffra),

Abbreviations used in this paper: **aRR, adjusted risk ratio; ALT, alanine aminotransferase; APRI, aspartate aminotransferase to platelet ratio index; AST, aspartate aminotransferase; CC, compensated cirrhosis; CI, confidence interval; CVD, cardiovascular disease; DC, decompensated cirrhosis; EHR, electronic health record; ELF, enhanced liver fibrosis; ESLD, end-stage liver disease; FIB-4, Fibrosis-4 index; HCC, hepatocellular carcinoma; HCRU, health-care resource utilization; ICD-10, International Classification of Diseases, 10th Revision; IQR, interquartile range; LT, liver transplantation; MASH, metabolic dysfunction–associated steatohepatitis; MASLD, metabolic dysfunction–associated steatotic liver disease; MRE, magnetic resonance elastography; MRI, magnetic resonance imaging; NIT, noninvasive test; PPPY, per-person-per-year; T2DM, type 2 diabetes mellitus.**

Most current article

© 2026 The Author(s). Published by Elsevier Inc. on behalf of American Gastroenterological Association Institute. This is an open access article under the CC BY license (http://creativecommons.org/licenses/by/4.0/).
2772-5723
https://doi.org/10.1016/j.gastha.2026.101031

an oral, liver-directed thyroid hormone receptor beta agonist for adults with noncirrhotic MASH and moderate to advanced fibrosis.[13]

Despite rising prevalence, robust real-world evidence on MASH progression, its predictors, HCRU, and costs are limited.[10] To address this evidence gap, we conducted a population-based study using Optum's deidentified Market Clarity Data (Optum Market Clarity) to characterize the natural history and health-care impact of diagnosed MASH among US adults. Our objectives were to (1) describe patient characteristics around the time of MASH diagnosis (eg, comorbidities, cardiometabolic risk factors, and the use of noninvasive tests [NITs] and imaging), (2) evaluate disease progression over time by changes in NIT scores and incidence of advanced liver outcomes (eg, CC, DC, HCC, liver transplantation [LT]), and (3) quantify the impact of progression to ESLD on HCRU and costs.

Methods

Study Design and Data Source

We conducted a retrospective cohort study of US adults using Optum Market Clarity, which links electronic health records (EHRs) with administrative medical and pharmacy claims.[14] The dataset includes demographics, encounters, diagnoses, procedures, laboratory results, and medication orders/dispensing (Supplementary Methods). The study observation window was January 1, 2019, to March 31, 2025. Patients with MASH (International Classification of Diseases, 10th Revision [ICD-10] code K75.8) were identified between January 1, 2021, and March 31, 2024. The index date (day 0) was the first recorded diagnosis of MASH. We assessed comorbidities, metabolic risk factors, and medication use during the 12-month preindex period (days −365 to 0). We ascertained exclusion criteria and prior cirrhosis or ESLD during the 24-month preindex period (days −730 to 0). Recognizing that hepatic assessments (eg, NITs) may occur shortly after diagnosis, we included an index testing window from the MASH diagnosis date (day 0) through day 30 to capture key assessments (eg, liver function tests, NITs, imaging tests, and biopsies) obtained around diagnosis. Follow-up for these assessments began on day 31 and continued until disenrollment, a >30-day coverage gap, or death (whichever came first). Follow-up for progression outcomes, HCRU, and costs began on day 1 and continued until censor. ESLD stages were ordered by severity as CC, DC, HCC, and LT. See Supplementary Figure 1 for the study schematic.

Study Population

Eligible patients were adults aged ≥18 years with either ≥1 inpatient claim listing MASH as a primary or secondary diagnosis, or ≥2 outpatient claims for MASH on different dates, identified using ICD-10 code K75.81. Exclusions (Supplementary Table 1) were assessed during the preindex lookback (days −730 to 0) and included alternative chronic liver diseases and other prespecified conditions; patients with any resmetirom (Rezdiffra) exposure in this window were also excluded. Additional requirements included ≥24 months of enrollment prior to index and ≥12 months of continuous follow-up after index (unless deceased). Patients who developed a prespecified exclusionary condition during follow-up were removed from the cohort at the time the condition was first recorded.

The study population was stratified into 2 primary groups based on the presence or absence of ESLD at baseline. Among patients with baseline ESLD, we assigned a mutually exclusive ESLD category based on the most advanced ESLD manifestation observed in the 2-year preindex period (hierarchy: LT > HCC > DC > CC), using diagnosis and procedure codes (Supplementary Table 2). Patients without baseline ESLD were monitored for progression to ESLD stages during follow-up.

Outcomes

Study outcomes included the use of NITs, imaging tests, blood-based biomarker tests, and liver biopsy during the index diagnosis testing window and over follow-up, stratified by ESLD status (Supplementary Table 2). Among patients without baseline ESLD, liver disease progression was defined using validated diagnosis and procedure codes as first occurrence of CC, DC, HCC, LT, or death. Progression was defined by the first qualifying event after index and assumed to be unidirectional (ie, no reversion to earlier stages). All time-to-event analyses used this "first ESLD event" definition; for descriptive tables, progressors were additionally grouped into mutually exclusive ESLD categories using the LT > HCC > DC > CC hierarchy.

Baseline characteristics and covariates were evaluated as potential predictors of progression. NIT and imaging results (eg, magnetic resonance elastography [MRE], transient elastography, enhanced liver fibrosis [ELF]) were captured during the index diagnosis testing window and over follow-up. We derived the following 2 composite indices from routinely collected laboratory data (alanine aminotransferase [ALT], aspartate aminotransferase [AST], platelet count): Fibrosis-4 index (FIB-4) and Aspartate Aminotransferase to Platelet Ratio Index (APRI; definitions in Supplementary Table 2). Indices were summarized separately around the index testing window (days 0–30) and at the first available follow-up (day ≥31) using available-case data (no imputation). For each index, we reported the number of patients with all required components within the window, the median (interquartile range [IQR]), and the proportions below and above clinically relevant thresholds defined in practice guidelines.[15,16]

A composite clinical end point was defined as first occurrence of CC (among those without baseline ESLD), DC, HCC, LT, or all-cause mortality.

HCRU included inpatient admissions, emergency department visits, outpatient encounters (generalist and specialist), and medication dispensing. Encounter definitions were standardized by claim identifier and date to reflect billing granularity (Supplementary Table 2).

Statistical Analysis

Patient characteristics, testing patterns, treatments and outcomes were summarized descriptively; categorical variables were expressed as counts and percentages, while continuous variables were expressed as means with standard deviations or medians with IQRs. For descriptive summaries of ESLD progression, patients were grouped according to the worst ESLD stage reached during follow-up using the

predefined hierarchy (LT > HCC > DC > CC), whereas all time-to-event end points were defined using time from index to the first ESLD event of any type. Kaplan–Meier methods were used to summarize time to first event and to report median time-to-event (IQR) for each progression outcome category. For first ESLD event subtypes (CC, DC, HCC, LT), cumulative incidence functions under a competing-risks framework were estimated and plotted. For evaluation of progression, a multivariable Cox proportional hazards model was used to estimate adjusted hazard ratios (aHRs) with 95% confidence intervals (CIs), adjusting for demographic, regional, comorbidity, and clinical risk factors.

Cardiometabolic risk factors, including metabolic syndrome, hypertension, dyslipidemia, T2DM, and obesity, were evaluated overall and by subgroup, and patients were further classified by the number of risk factors present. Other comorbidities such as polycystic ovary syndrome, menopause, and sleep apnea were also assessed (Supplementary Table 2).

HCRU counts were modeled with multivariable negative binomial regression (offset for follow-up person-time) to estimate marginal means. For marginal means, 95% CIs were derived using a nonparametric patient-level bootstrap with 500 resamples (refitting all model components per replicate and using percentile intervals). Costs (presented in 2024 United States Dollar) were analyzed per-person-per-year (PPPY) using generalized linear models with log link and gamma distribution; two-part models were used if zero-inflation was evident. Because cost distributions are typically right skewed, both mean and median PPPY costs were reported. Multivariable models included adjustment for prespecified covariates (baseline demographics, comorbidities, and prior health utilization) and included robust variance estimators. Missingness in labs/NITs was handled using available-case analysis; components required to compute composite indices were not imputed. Analyses were conducted using R (version 4.5.1; R Foundation for Statistical Computing, Vienna, Austria). A two-sided $\alpha = 0.05$ defined statistical significance.

Sensitivity Analyses

To ensure the robustness of our findings, various sensitivity analyses were performed. First, we expanded the index diagnosis testing window to 15 days before index through 90 days after index to better capture NITs and laboratory measures in settings without same-day testing. Next, we expanded the MASH cohort to include MASLD patients (ICD-10-CM K76.0) and repeated several analyses with this larger cohort to contextualize disease burden and progression among the broader steatotic liver disease spectrum. Lastly, we repeated the progression and HCRU/cost analyses after reclassifying patients who experienced an ESLD event within the first 30 days after index as having baseline ESLD, to assess the potential impact of misclassifying prevalent ESLD as incident progression.[17]

This study used deidentified data from Optum Market Clarity that are compliant with the Health Insurance Portability and Accountability Act; therefore, institutional review board review was not required. All work adhered to the principles of the Declaration of Helsinki. Data access was governed by a data-use agreement with Optum.

Reporting followed the Reporting of studies Conducted using Observational Routinely collected health Data statement, an extension of the Strengthening the Reporting of Observational Studies in Epidemiology guidelines for observational studies using routinely collected health data[18]; see Supplementary Methods for completed checklist.

Results

Baseline Characteristics

From over 9 million individuals in Optum Market Clarity during the study period, 49,983 patients with MASH met inclusion criteria and comprised the study cohort (Figure 1). At baseline, 16,359 (32.7%) had evidence of ESLD—3965 with CC, 11,406 with DC, 514 with HCC, and 474 with prior LT. Among those without ESLD at baseline (n = 33,624), 5371 (15.9%) progressed to ESLD during follow-up and 28,253 did not. Median (IQR) follow-up was slightly longer for patients without ESLD at baseline (2.3 [1.7–3.1] years) than for those with ESLD (2.1 [1.5–2.9] years).

Baseline demographic and clinical characteristics, comorbidities, and metabolic risk factors are shown in Table 1. At index, patients with ESLD were older (mean age 63.3 years vs 55.4 years) and more likely to be female (63.3% vs 57.9%) compared with those without ESLD. A higher mean (standard deviation) comorbidity burden was seen in patients with ESLD at baseline on both the weighted Elixhauser Comorbidity Index (7.1 [9.3] vs 1.7 [6.9]) as well as the Diabetes Complications Severity Index (1.6 [1.5] vs 0.7 [1.0]). These patients exhibited a higher prevalence of CVD, anemia, and abdominal/pelvic pain as compared to those without ESLD at baseline. Metabolic risk factors, including obesity, T2DM, hypertension, and dyslipidemia, were common in both groups, though hypertension and T2DM were more frequent among patients with ESLD.

Among patients without ESLD at baseline, those who progressed to ESLD during follow-up were generally older (59.0 vs 54.7 years), more likely to be female (60.9% vs 57.3%) and had longer median (IQR) length of follow-up (2.5 [1.8–3.3] vs 2.3 [1.6–3.0] years) compared with those without ESLD. Patients who progressed to ESLD also had higher rates of most cardiometabolic risk factors, including hypertension (65.4% vs 54.9%), T2DM (48.3% vs 36.7%), obesity (67.3% vs 64.8%), and dyslipidemia (76.9% vs 74.6%) compared with those who remained free of progression.

Treatment Patterns at Baseline

At baseline, 15.5% of the entire cohort had evidence of glucagon-like peptide-1 receptor agonists use, most commonly semaglutide (7.7%) and dulaglutide (5.8%), with other agents used infrequently (Table 1). Nearly half of the cohort were on a statin (45.9%), whereas SGLT2 inhibitor use was lower (11.8%), with empagliflozin (5.8%) being the most frequently used drug in that class. Use of clopidogrel was uncommon (3.4%). Across all classes, medication use was consistently higher among patients

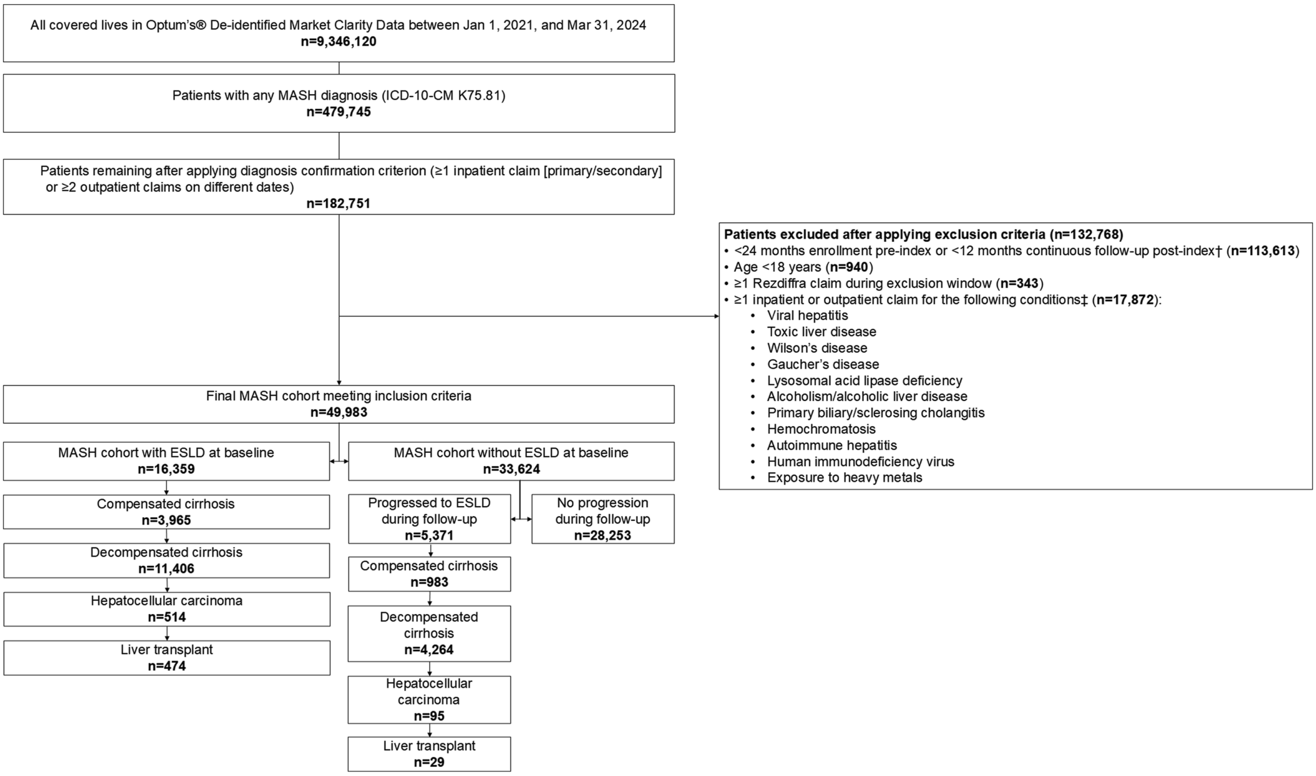

Figure 1. Study cohort diagram. ESLD, end-stage liver disease; ICD-10-CM, International Classification of Diseases 10th revision Clinical Modification; MASH, metabolic dysfunction–associated steatohepatitis; T2DM, type 2 diabetes mellitus. †This criteria is applied to all patients unless the patient had died during this time. ‡Diagnostic codes specified in Supplementary Table 1.

with baseline ESLD than those without ESLD, including any glucagon-like peptide-1 receptor agonists (17.9% vs 14.3%), any SGLT2 inhibitor (16.0% vs 9.7%), any statin (50.5% vs 43.7%), and clopidogrel (5.5% vs 2.3%; Table 1).

Noninvasive Tests, Imaging, Liver Tests, Biopsy, and Fibrosis Indices

The use of NITs, imaging, liver function blood tests, and liver biopsies during the index diagnosis window (days 0–30) varied by baseline ESLD status (Table 2). Overall, 3771 (7.5%) patients received ≥1 NIT or imaging test; uptake was higher with baseline ESLD (1318/16,359; 8.1%) than without (2453/33,624; 7.3%). Of those patients, 868 received only magnetic resonance imaging (MRI; 1.7%); uptake was higher with baseline ESLD (549/16,359; 3.4%) than without (319/33,624; 1.0%). Transient elastography was seen in 4.0% and 5.5%, respectively, while ELF was not observed.

During follow-up (day 31+), NIT and imaging test utilization increased. Among patients with baseline ESLD, 26.5% (4333/16,359) had ≥1 NIT or imaging test vs 18.0% (6050/33,624) without baseline ESLD (Table 2). Of those patients, 14.4% (2360/16,369) received only MRI with baseline ESLD vs 4.4% (1465/33,624) without baseline ESLD. MRI was frequently performed in patients with baseline ESLD (17.6%), whereas transient elastography predominated without baseline ESLD (11.4%). Other modalities—including MRE (1.3% and 1.1%), FibroSure/FibroTest (1.3% and 1.6%), LiverMultiScan (<0.1% and 0.1%), and ELF (0.1% and 0.2%)—were rare. Liver biopsy was slightly higher with baseline ESLD (4.6%) than without (3.7%).

Laboratory assessments were consistently more common in patients with baseline ESLD (Table 2). Within the index window, bilirubin and albumin/total protein were obtained in 14.5% and 14.6% with ESLD vs 11.5% and 11.4% without; AST and ALT were each measured in 14.1% and 14.2% vs 11.2% and 11.3%. Liver biopsies were similar in both groups (4.2% vs 3.7%). During follow-up, frequency of testing increased, including albumin/total protein (23.1% vs 19.8%), bilirubin (22.7% vs 19.5%), AST (22.5% vs 19.3%), and ALT (22.5% vs 19.4%), with the largest differential for prothrombin time (16.6% with ESLD vs 9.9% without). Additionally, the proportion of patients with computable indices increased at follow-up: both FIB-4 and Aspartate Aminotransferase to Platelet Ratio Index were calculable for 18.7% of patients with baseline ESLD and 17.7% without.

Disease Progression Events and Predictors of Disease Progression

Among patients without ESLD at baseline (n = 33,624), 15.9% (n = 5371) progressed to an ESLD

Table 1. Baseline Characteristics of Patients Diagnosed With MASH by Baseline ESLD and Progression Status

Characteristic	Total (n = 49,983) n (%)[a]	With ESLD at baseline (n = 16,359) n (%)[a]	Without ESLD at baseline (n = 33,624) n (%)[a]	With progression (n = 5371) n (%)[a]	Without progression (n = 28,253) n (%)[a]
Length of follow-up (y)					
Mean (SD)	2.3 (0.9)	2.2 (1.0)	2.4 (0.9)	2.5 (0.9)	2.4 (0.9)
Median (IQR)	2.2 (1.6–3.0)	2.1 (1.5–2.9)	2.3 (1.7–3.1)	2.5 (1.8–3.3)	2.3 (1.6–3.0)
Sociodemographic information					
Age at index (y)					
Mean (SD)	58.0 (13.9)	63.3 (12.6)	55.4 (13.7)	59.0 (13.6)	54.7 (13.6)
Median (IQR)	59 (49–68)	64 (56–73)	56 (47–65)	60 (50–69)	56 (46–64)
Age category (y)					
18–44 y	8545 (17.1)	1353 (8.3)	7192 (21.4)	815 (15.2)	6377 (22.6)
45–64 y	24,799 (49.6)	6943 (42.4)	17,856 (53.1)	2589 (48.2)	15,267 (54.0)
≥65 y	16,639 (33.3)	8063 (49.3)	8576 (25.5)	1967 (36.6)	6609 (23.4)
Sex					
Female	29,809 (59.6)	10,354 (63.3)	19,455 (57.9)	3271 (60.9)	16,184 (57.3)
Male	20,139 (40.3)	5989 (36.6)	14,150 (42.1)	2097 (39.0)	12,053 (42.7)
Unknown	35 (0.1)	16 (0.1)	19 (0.1)	3 (0.1)	16 (0.1)
Race					
White	39,072 (78.2)	13,546 (82.8)	25,526 (75.9)	4241 (79.0)	21,285 (75.3)
Black	2159 (4.3)	563 (3.4)	1596 (4.7)	228 (4.2)	1368 (4.8)
Asian	1816 (3.6)	351 (2.1)	1465 (4.4)	190 (3.5)	1275 (4.5)
Other/unknown	6936 (13.9)	1899 (11.6)	5037 (15.0)	712 (13.3)	4325 (15.3)
Ethnicity					
Hispanic	6454 (12.9)	1879 (11.5)	4575 (13.6)	676 (12.6)	3899 (13.8)
Non-Hispanic	37,106 (74.2)	12,583 (76.9)	24,523 (72.9)	4032 (75.1)	20,491 (72.5)
Unknown	6423 (12.9)	1897 (11.6)	4526 (13.5)	663 (12.3)	3863 (13.7)
Geographic region					
Northeast	11,415 (22.8)	3143 (19.2)	8272 (24.6)	1218 (22.7)	7054 (25.0)
Midwest	16,667 (33.3)	6129 (37.5)	10,538 (31.3)	1779 (33.1)	8759 (31.0)
South	13,697 (27.4)	4638 (28.4)	9059 (26.9)	1492 (27.8)	7567 (26.8)
West	5925 (11.9)	1752 (10.7)	4173 (12.4)	610 (11.4)	3563 (12.6)
Unknown	2279 (4.6)	697 (4.3)	1582 (4.7)	272 (5.1)	1310 (4.6)
Insurance type					
Commercial	17,435 (34.9)	4907 (30.0)	12,528 (37.3)	1813 (33.8)	10,715 (37.9)
Medicaid	1742 (3.5)	621 (3.8)	1121 (3.3)	187 (3.5)	934 (3.3)
Medicare	6279 (12.6)	3274 (20.0)	3005 (8.9)	702 (13.1)	2303 (8.2)
Clinical characteristics					
BMI, kg/m^2	14,203 (28.4)	4987 (30.5)	9216 (27.4)	1446 (26.9)	7770 (27.5)
Mean (SD)	34.8 (7.9)	34.8 (8.2)	34.8 (7.6)	35.4 (8)	34.7 (7.6)
Median (IQR)	33.7 (29.4–39.1)	33.6 (29–39.4)	33.7 (29.6–38.9)	34.3 (29.7–39.5)	33.6 (29.5–38.8)
BMI categories, kg/m^2					
<25	919 (1.8)	401 (2.5)	518 (1.5)	97 (1.8)	421 (1.5)
25–<30	3078 (6.2)	1085 (6.6)	1993 (5.9)	285 (5.3)	1708 (6.0)
≥30	10,206 (20.4)	3501 (21.4)	6705 (19.9)	1064 (19.8)	5641 (20.0)
Unknown	35,780 (71.6)	11,372 (69.5)	24,408 (72.6)	3925 (73.1)	20,483 (72.5)
Comorbidities					
Cardiometabolic risk factors					
Metabolic syndrome	10,233 (20.5)	3508 (21.4)	6725 (20.0)	1070 (19.9)	5655 (20.0)
Hypertension	31,029 (62.1)	11,999 (73.3)	19,030 (56.6)	3515 (65.4)	15,515 (54.9)
Dyslipidemia	37,825 (75.7)	12,617 (77.1)	25,208 (75.0)	4129 (76.9)	21,079 (74.6)
Obesity	33,045 (66.1)	11,136 (68.1)	21,909 (65.2)	3615 (67.3)	18,294 (64.8)
T2DM	23,277 (46.6)	10,307 (63.0)	12,970 (38.6)	2595 (48.3)	10,375 (36.7)
≥1 cardiometabolic risk factor	46,980 (94.0)	15,631 (95.5)	31,349 (93.2)	5075 (94.5)	26,274 (93.0)
≥2 cardiometabolic risk factors	39,612 (79.3)	14,014 (85.7)	25,598 (76.1)	4374 (81.4)	21,224 (75.1)
≥3 cardiometabolic risk factors	28,695 (57.4)	11,133 (68.1)	17,562 (52.2)	3219 (59.9)	14,343 (50.8)
Other comorbidities of interest					
Abdominal/pelvic pain	16,415 (32.8)	6719 (41.1)	9696 (28.8)	1892 (35.2)	7804 (27.6)

Table 1. Continued

Characteristic	Total (n = 49,983) n (%)[a]	With ESLD at baseline (n = 16,359) n (%)[a]	Without ESLD at baseline (n = 33,624) n (%)[a]	With progression (n = 5371) n (%)[a]	Without progression (n = 28,253) n (%)[a]
Anemia	9819 (19.6)	5777 (35.3)	4042 (12.0)	975 (18.2)	3067 (10.9)
Bariatric surgery	371 (0.7)	118 (0.7)	253 (0.8)	31 (0.6)	222 (0.8)
Cardiovascular disease	21,011 (42.0)	9831 (60.1)	11,180 (33.3)	2401 (44.7)	8779 (31.1)
Fatigue/insomnia	15,737 (31.5)	6623 (40.5)	9114 (27.1)	1729 (32.2)	7385 (26.1)
Peptic ulcer disease, dyspepsia, GERD, esophagitis	21,200 (42.4)	8871 (54.2)	12,329 (36.7)	2498 (46.5)	9831 (34.8)
Renal impairment	7758 (15.5)	4554 (27.8)	3204 (9.5)	800 (14.9)	2404 (8.5)
Sleep apnea	15,715 (31.4)	5960 (36.4)	9755 (29.0)	1868 (34.8)	7887 (27.9)
Smoking (current or past)	10,775 (21.6)	4923 (30.1)	5852 (17.4)	1274 (23.7)	4578 (16.2)
T1DM	1307 (2.6)	724 (4.4)	583 (1.7)	165 (3.1)	418 (1.5)
Thyroid disease	12,373 (24.8)	4821 (29.5)	7552 (22.5)	1428 (26.6)	6124 (21.7)
Thyroid cancer	355 (0.7)	116 (0.7)	239 (0.7)	40 (0.7)	199 (0.7)
Vitamin D deficiency	14,561 (29.1)	4691 (28.7)	9870 (29.4)	1672 (31.1)	8198 (29.0)
PCOS[b]	1026 (2.1)	217 (1.3)	809 (2.4)	97 (1.8)	712 (2.5)
Menopause[b]	2926 (5.9)	913 (5.6)	2013 (6.0)	325 (6.1)	1688 (6.0)
End-stage renal disease	563 (1.1)	471 (2.9)	92 (0.3)	27 (0.5)	65 (0.2)
Elixhauser Comorbidity Index					
Unweighted					
Mean (SD)	3.9 (2.7)	5.6 (2.9)	3.1 (2.1)	4 (2.4)	3 (2)
Median (IQR)	3 (2–5)	5 (3–7)	3 (2–4)	4 (2–5)	3 (2–4)
Weighted					
Mean (SD)	3.5 (8.2)	7.1 (9.3)	1.7 (6.9)	3.2 (7.8)	1.4 (6.7)
Median (IQR)	2 (−2 to 7)	6 (0–13)	1 (−3 to 6)	2 (−2 to 7)	0 (−3 to 5)
Diabetes Complications Severity Index (DCSI)					
Mean (SD)	1.0 (1.3)	1.6 (1.5)	0.7 (1.0)	1.1 (1.3)	0.6 (1.0)
Median (IQR)	1.0 (0–2)	1.0 (0–2)	0 (0–1)	1.0 (0–2)	0 (0–1)
Medication use at baseline					
GLP-1 receptor agonists (any)	7748 (15.5)	2925 (17.9)	4823 (14.3)	917 (17.1)	3906 (13.8)
Semaglutide	3832 (7.7)	1333 (8.1)	2499 (7.4)	442 (8.2)	2057 (7.3)
Liraglutide	1383 (2.8)	529 (3.2)	854 (2.5)	149 (2.8)	705 (2.5)
Dulaglutide	2879 (5.8)	1158 (7.1)	1721 (5.1)	368 (6.9)	1353 (4.8)
Tirzepatide	425 (0.9)	132 (0.8)	293 (0.9)	36 (0.7)	257 (0.9)
Exenatide	312 (0.6)	124 (0.8)	188 (0.6)	45 (0.8)	143 (0.5)
Lixisenatide	101 (0.2)	53 (0.3)	48 (0.1)	10 (0.2)	38 (0.1)
Statin use (any)	22,961 (45.9)	8260 (50.5)	14,701 (43.7)	2639 (49.1)	12,062 (42.7)
SGLT2 inhibitors (any)	5876 (11.8)	2615 (16)	3261 (9.7)	704 (13.1)	2557 (9.1)
Canagliflozin	434 (0.9)	178 (1.1)	256 (0.8)	49 (0.9)	207 (0.7)
Dapagliflozin	1326 (2.7)	561 (3.4)	765 (2.3)	145 (2.7)	620 (2.2)
Empagliflozin	2920 (5.8)	1219 (7.5)	1701 (5.1)	339 (6.3)	1362 (4.8)
Ertugliflozin	147 (0.3)	55 (0.3)	92 (0.3)	14 (0.3)	78 (0.3)
Bexagliflozin	0 (0.0)	0 (0.0)	0 (0.0)	0 (0.0)	0 (0.0)
Clopidogrel	1677 (3.4)	904 (5.5)	773 (2.3)	231 (4.3)	542 (1.9)

BMI, body mass index; DCSI, Diabetes Complications Severity Index; ESLD, end-stage liver disease; GERD, gastroesophageal reflux disease; GLP-1, glucagon-like peptide-1; IQR, interquartile range; MASH, metabolic dysfunction–associated steatohepatitis; PCOS, polycystic ovary syndrome; SD, standard deviation; T1DM, type 1 diabetes mellitus; T2DM, type 2 diabetes mellitus.
[a]n (%) unless otherwise specified.
[b]PCOS and menopause were identified among women only.

Table 2. Use of Noninvasive Tests (NITs), Liver Function Blood Tests, and Liver Biopsies Within MASH Index Diagnosis Testing Window (Day 0, 30) and During Follow-Up by Baseline ESLD Status

	Within index diagnosis testing window (day 0, 30)		During follow-up (day 31, end of follow-up)	
	With ESLD at baseline (n = 16,359)	Without ESLD at baseline (n = 33,624)	With ESLD at baseline (n = 16,359)	Without ESLD at baseline (n = 33,624)
Test type	n (%)[a]	n (%)[a]	n (%)[a]	n (%)[a]
Noninvasive tests (NITs) and imaging				
At least 1 NIT or imaging test (any)	1318 (8.1)	2453 (7.3)	4333 (26.5)	6050 (18.0)
Transient elastography	650 (4.0)	1835 (5.5)	1623 (9.9)	3817 (11.4)
Enhanced liver fibrosis (ELF)	0 (0.0)	0 (0.0)	19 (0.1)	64 (0.2)
Magnetic resonance elastography (MRE)	37 (0.2)	85 (0.3)	212 (1.3)	366 (1.1)
LiverMultiScan	n < 5[b]	n < 5[b]	7 (<0.1)	44 (0.1)
Fibrosure/Fibrotest	87 (0.5)	236 (0.7)	214 (1.3)	535 (1.6)
Magnetic resonance imaging (MRI)	603 (3.7)	414 (1.2)	2881 (17.6)	2168 (6.4)
Liver biopsy	694 (4.2)	1253 (3.7)	746 (4.6)	1242 (3.7)
Liver function blood tests				
Albumin and total protein	2389 (14.6)	3841 (11.4)	3779 (23.1)	6647 (19.8)
Bilirubin	2373 (14.5)	3868 (11.5)	3721 (22.7)	6552 (19.5)
Alkaline phosphatase (ALP)	2286 (14.0)	3639 (10.8)	3655 (22.3)	6455 (19.2)
Aspartate aminotransferase (AST)	2312 (14.1)	3764 (11.2)	3677 (22.5)	6506 (19.3)
Alanine aminotransferase (ALT)	2326 (14.2)	3788 (11.3)	3686 (22.5)	6521 (19.4)
Gamma-glutamyltransferase (GGT)	177 (1.1)	355 (1.1)	1901 (11.6)	3027 (9.0)
L-lactate dehydrogenase (LD)	222 (1.4)	114 (0.3)	1811 (11.1)	2703 (8.0)
Platelet count (PLT)	1701 (10.4)	2270 (6.8)	3492 (21.3)	6712 (20.0)
Prothrombin time (PT)	1288 (7.9)	702 (2.1)	2711 (16.6)	3334 (9.9)
Fasting glucose	21 (0.1)	67 (0.2)	111 (0.7)	314 (0.9)
Laboratory-based indices				
FIB-4, computable n (%)	1942 (11.8)	2662 (7.9)	3053 (18.7)	5963 (17.7)
Median (IQR)	2.6 (1.4–5.3)	1.2 (0.8–1.8)	2.3 (1.3–4.4)	1.2 (0.8–1.7)
FIB-4 <1.3[c]	410 (21.1)	1503 (56.5)	765 (25.0)	3311 (55.5)
FIB-4 ≥2.67[c]	960 (49.4)	282 (10.6)	1305 (42.7)	487 (8.2)
APRI, computable n (%)	1947 (11.9)	2678 (8.0)	3062 (18.7)	5971 (17.7)
Median (IQR)	0.5 (0.3–1.1)	0.3 (0.2–0.5)	0.4 (0.3–0.9)	0.3 (0.2–0.4)
APRI <1[c]	1433 (73.6)	2444 (91.3)	2438 (79.6)	5722 (95.8)
APRI ≥2[c]	203 (10.4)	107 (4.0)	192 (6.3)	46 (0.8)

ALP, alkaline phosphatase; ALT, alanine aminotransferase; APRI, Aspartate Aminotransferase to Platelet Ratio Index; AST, aspartate aminotransferase; CT, computed tomography; ELF, enhanced liver fibrosis; ESLD, end-stage liver disease; FIB-4, Fibrosis-4 index; GGT, gamma-glutamyltransferase; LD, L-lactate dehydrogenase; MRE, magnetic resonance elastography; MRI, magnetic resonance imaging; NIT, noninvasive test; PLT, platelet count; PT, prothrombin time.
[a]n (%) unless otherwise specified.
[b]Per agreement with data provider, results with n < 5 have been redacted.
[c]Percentages are calculated among patients with a computable index in the given window/stratum (ie, the denominator is those patients). The extreme categories (eg, FIB-4 <1.3 and ≥2.67; APRI <1 and ≥2) will not sum to 100% because the indeterminate band is not shown.

event during follow-up. DC was the most frequent first event (12.7%; n = 4264), followed by CC (2.9%; n = 983), HCC (0.3%; n = 95), and LT (0.1%; n = 29). The median time to first ESLD event was 10.6 months (IQR: 3.8–19.7), with time to CC shortest (8.1 months, 2.5–16.9) and time to LT longest among ESLD components (14.7 months [IQR: 9.2–24.4]). All-cause death occurred in 1.8% (n = 601; median 22.3 months [IQR: 10.5–33.5]).

Progression was slightly higher among patients with multiple comorbidities and occurred on a similar timeline (Table 3). The proportion progressing to any ESLD event was 11.0% among those with ≥2 cardiometabolic risk factors (n = 4374/39,612; median time from index 10.9 months [IQR: 4.0–20.1]), 11.2% with ≥3 risk factors (n = 3219/28,695; 10.7 months [IQR: 3.8–19.8]), 10.9% with obesity (n = 3615/33,045; 10.3 months [IQR: 3.6–19.7]), and 11.1% with T2DM (n = 2595/23,277; 10.8 months [IQR: 3.9–19.5]). Within these subgroups, DC remained the dominant first event (∼8%–9%), CC occurred in ∼2%, and HCC and LT were rare (≤0.2% and ≤0.1%, respectively); median times from index were closely aligned with the overall cohort (CC ∼8 months; DC ∼11 months).

In adjusted analyses (Figure 2; Supplementary Table 9), older age was associated with a slightly higher risk of progression (adjusted risk ratio [aRR]: 1.02 [95% CI: 1.02–1.02]). Compared with Caucasian patients, African American patients had lower risk of disease progression (aRR: 0.83 [95% CI: 0.74–0.94]). Relative to the Northeast, progression risk was modestly higher in the Midwest (aRR: 1.07 [95% CI: 1.00–1.14]) and South regions (aRR: 1.07 [95% CI: 1.00–1.14]). Presence of metabolic and clinical factors independently associated with higher progression risk included hypertension (aRR: 1.18 [95% CI: 1.12–1.26]), type 2 diabetes (aRR: 1.25 [95% CI: 1.19–1.32]), CVD (aRR: 1.18 [95% CI: 1.12–1.24]), sleep apnea (aRR: 1.13 [95% CI: 1.07–1.19]), smoking (aRR: 1.23 [95% CI: 1.16–1.30]), and thyroid disease (aRR: 1.11 [95% CI: 1.05–1.17]). Obesity, vitamin D deficiency and end-stage renal disease were not associated with a higher risk of progression after adjustment (Supplementary Table 9). Dyslipidemia showed an inverse association after adjustment (aRR: 0.80 [95% CI: 0.76–0.85]).

Cumulative Incidence of Death and First End-Stage Liver Disease Events

Cumulative incidence of all-cause mortality was consistently higher among MASH patients who progressed than among nonprogressors (Figure 3A). The curves separated early and continued to diverge over follow-up. By 24 months (730 days), mortality was 3.51% in progressors vs 0.59% in nonprogressors; by 47 months (1420 days), it was 10.75% vs 2.64%. Despite a smaller at-risk population at baseline (5371 vs 28,253),

Table 3. Progression Events Among Patients With MASH Without Baseline ESLD, Overall, and in High-Risk Subgroups

Progression category	Definition	Without ESLD at baseline (n = 33,624)		≥2 cardiometabolic risk factors (n = 39,612)[a]		≥3 cardiometabolic risk factors (n = 28,695)[a]		Obesity (n = 33,045)		T2DM (n = 23,277)	
		n (%)	Median (IQR)[b]	n (%)	Median (IQR)[b]	n (%)	Median (IQR)[b]	n (%)	Median (IQR)[b]	n (%)	Median (IQR)[b]
Compensated cirrhosis (CC)	CC, no prior DC/HCC/LT	983 (2.9)	8.1 (2.5–16.9)	799 (2.0)	8.3 (2.5–17.5)	602 (2.1)	7.9 (2.2–16.0)	672 (2.0)	8.1 (2.5–16.4)	530 (2.3)	8.2 (2.7–16.2)
Decompensated cirrhosis (DC)	DC, no prior HCC/LT	4264 (12.7)	11.1 (4.1–20.3)	3475 (8.8)	11.4 (4.5–20.7)	2546 (8.9)	11.3 (4.3–20.5)	2862 (8.7)	10.8 (3.9–20.3)	2001 (8.6)	11.4 (4.4–20.2)
Hepatocellular carcinoma (HCC)	HCC, no prior LT	95 (0.3)	10.2 (4.2–18.5)	79 (0.2)	10.6 (4.8–18.5)	56 (0.2)	10.7 (5.9–19.4)	64 (0.2)	10.1 (5.0–18.9)	49 (0.2)	10.6 (5.6–22.2)
Liver transplantation (LT)	LT, regardless of previous state	29 (0.1)	14.7 (9.2–24.4)	21 (0.1)	18.1 (12.1–24.9)	15 (0.1)	21.0 (18.0–28.6)	17 (0.1)	19.1 (13.6–26.7)	15 (0.1)	19.1 (12.4–25.8)
Death	All-cause	601 (1.8)	22.3 (10.5–33.5)	535 (1.4)	22.7 (11.4–33.8)	399 (1.4)	23.5 (12.3–34.1)	363 (1.1)	23.8 (12.1–34.6)	350 (1.5)	22.2 (11.3–33.9)
Composite ESLD progression	First of CC, DC, HCC, LT	5371 (15.9)	10.6 (3.8–19.7)	4374 (11.0)	10.9 (4.0–20.1)	3219 (11.2)	10.7 (3.8–19.8)	3615 (10.9)	10.3 (3.6–19.7)	2595 (11.1)	10.8 (3.9–19.5)

CC, compensated cirrhosis; DC, decompensated cirrhosis; ESLD, end-stage liver disease; HCC, hepatocellular carcinoma; IQR, interquartile range; LT, liver transplantation; T2DM, type 2 diabetes mellitus.
[a]Cardiometabolic risk factors include metabolic syndrome, hypertension, dyslipidemia, T2DM, and obesity.
[b]Median time-to-event (months).

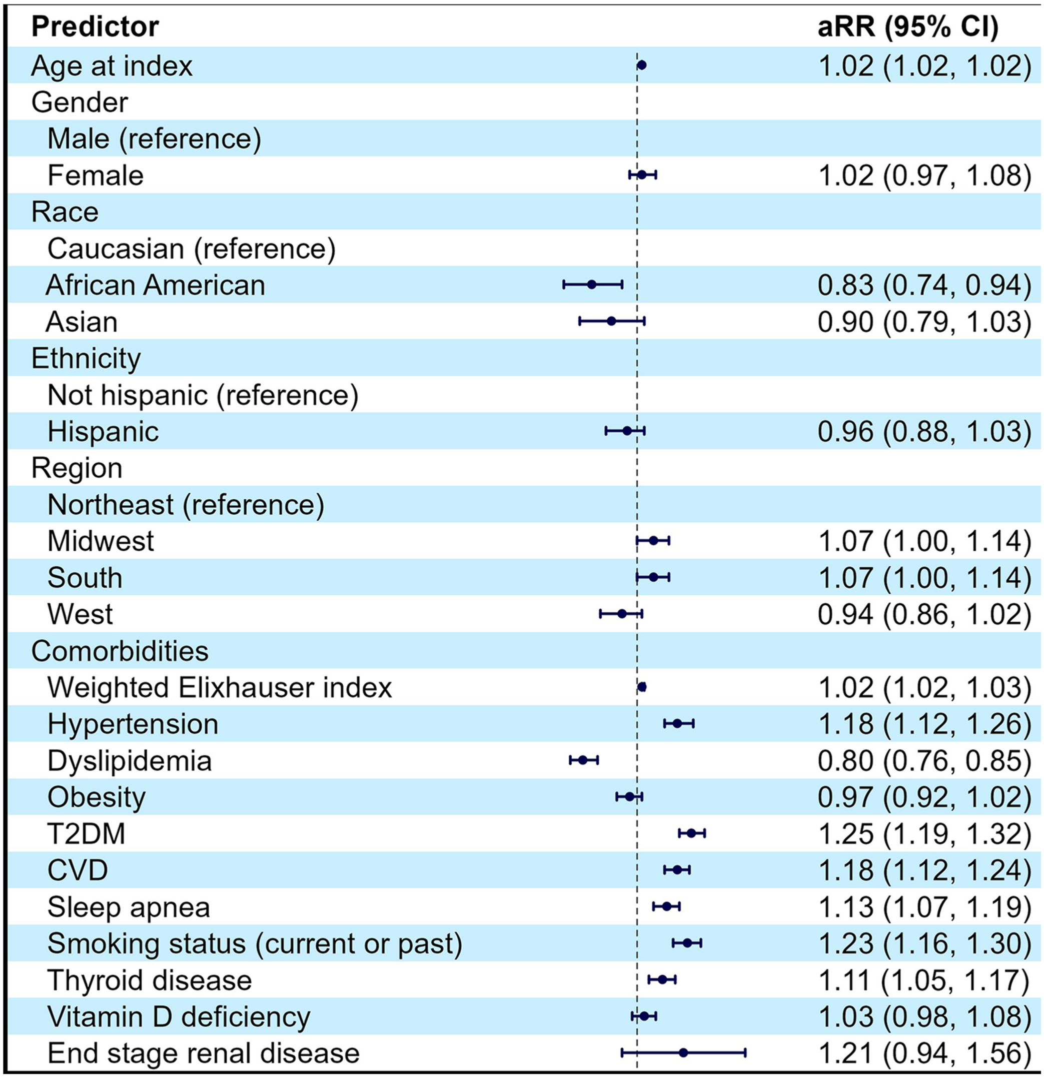

Figure 2. Predictors of composite liver disease progression: adjusted relative risks (aRR)[†]. aRR, adjusted relative risk; CI, confidence interval; CVD, cardiovascular disease; RR, relative risk; T2DM, type 2 diabetes mellitus. [†]Model adjusted for age, gender, race, ethnicity, region, Elixhauser Comorbidity Index, hypertension, dyslipidemia, obesity, T2DM, smoking, CVD, sleep apnea, thyroid disease, vitamin D deficiency, and end-stage renal disease. See Supplementary Table 3 for unadjusted values.

more deaths accrued among progressors by approximately 4 years (317 vs 270).

Within the first year after MASH diagnosis, the cumulative incidence of first progression was highest for DC (∼13%), followed by CC (∼3%), HCC (<0.5%), and LT (<0.2%). Most events occurred early, within 30–60 days of diagnosis, after which the curves plateaued (Figure 3B).

Health-Care Resource Utilization and Costs

During follow-up, patients with ESLD at baseline (n = 16,359) incurred substantially higher PPPY total costs than those without ESLD (n = 33,624): mean $134,674 (median $71,527 [IQR: 27,483–164,022]) vs $70,159 (median $30,237 [IQR: 10,108–75,334]) (Supplementary Figure 2A); adjusted mean $27,372 (95% CI: 25,975–28,554) vs $21,489 (95% CI: 20,483–22,401) (Supplementary Table 4).

HCRU was consistently higher with baseline ESLD (Supplementary Figure 2B): adjusted inpatient admissions 0.6 vs 0.3 PPPY; ED visits 2.2 vs 1.5; outpatient visits 54.8 vs 45.0; GP visits 5.3 vs 4.3; specialist visits 47.5 vs 38.8; and total medication dispensations 93.8 vs 88.2 (Supplementary Table 4;). Component costs (PPPY-adjusted mean) showed the same pattern, ESLD vs no ESLD: inpatient $11,001 vs $6497; ED $1155 vs $843; outpatient $8156 vs $6710; GP $208 vs $179; specialist $8033 vs $6622; total medications $21,014 vs $13,289.

Among patients without ESLD at baseline, those who progressed (n = 5371) had higher total costs than

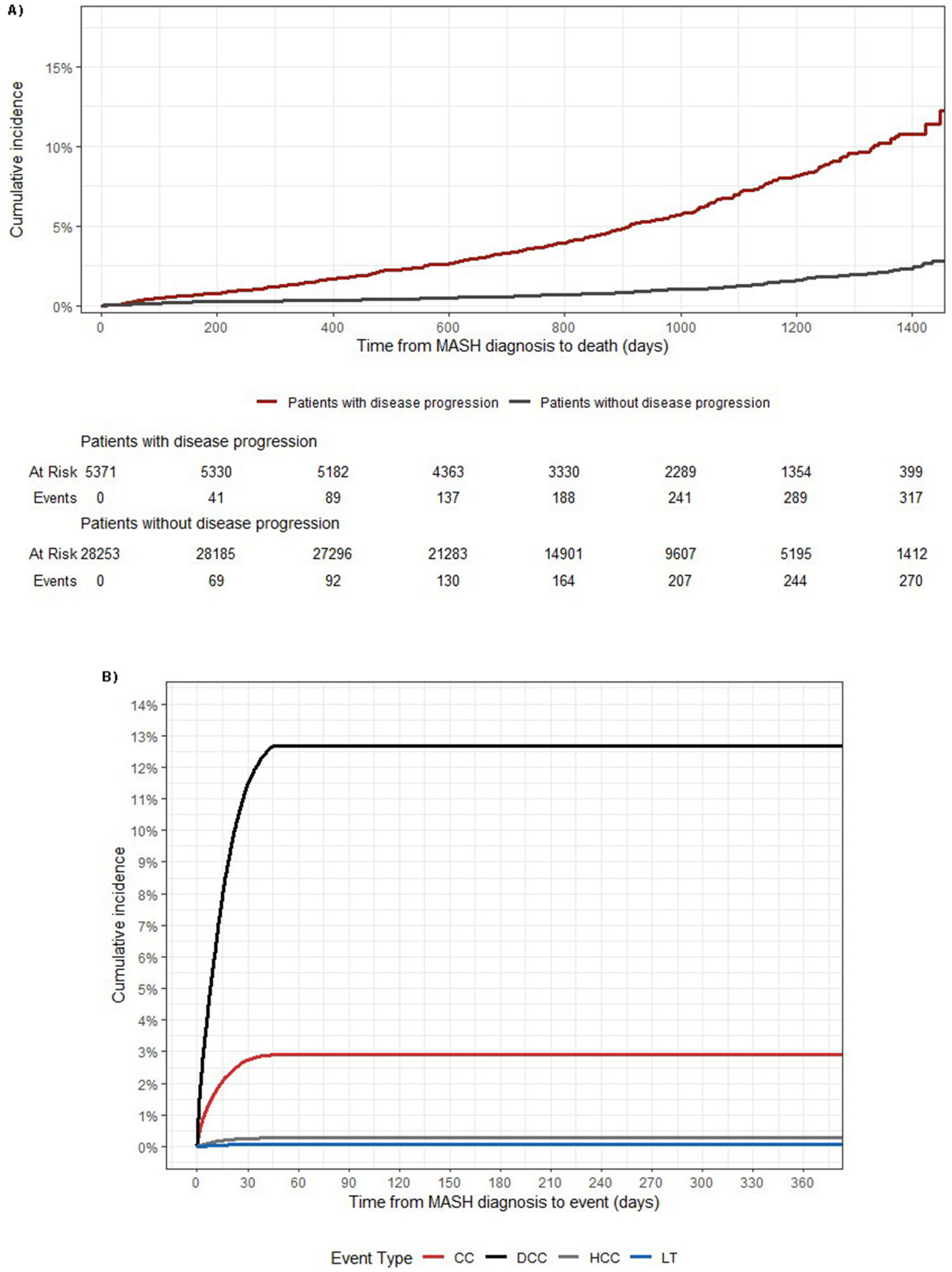

Figure 3. Cumulative incidence of (A) all-cause death after MASH diagnosis, by progression status and (B) first progression event type after MASH diagnosis among those without ESLD at baseline. CC, compensated cirrhosis; DCC, decompensated cirrhosis; ESLD, end-stage liver disease; HCC, hepatocellular carcinoma; LT, liver transplantation; MASH, metabolic dysfunction–associated steatohepatitis. Note: (A) X-axis truncated at 1,420 days for illustration purposes to avoid small–risk-set artifacts near study end; (B) X-axis truncated at 365 days for illustration purposes; curves plateau thereafter.

nonprogressors (n = 28,253) (Supplementary Figure 2A and B): adjusted mean $36,498 (95% CI 34,444–38,358) vs $16,305 (15,438–17,027) and greater utilization: inpatient admissions 0.7 vs 0.2 PPPY; ED visits 2.3 vs 1.1; outpatient visits 57.8 vs 40.4; GP visits 4.6 vs 3.2; specialist visits 51.8 vs 35.6; and total medication dispensations 89.1 vs 78.5 (Supplementary Table 5). Component costs (PPPY-adjusted mean) were likewise higher with progression: inpatient $18,506 vs $3308; ED $1647 vs $618;

outpatient $11,326 vs $5826; GP $262 vs $172; specialist $11,194 vs $5729; and medications $8471 vs $6300 (Supplementary Table 5).

Sensitivity Analyses

In the sensitivity analysis for which an extended index diagnosis testing window was considered (day −15 to day 90), overall ascertainment of testing increased but the relative patterns by ESLD status were unchanged compared with the main 0 to 30-day baseline window (Supplementary Table 6). Among patients with baseline ESLD, any NIT or imaging test rose from 8.1% (1318/16,359) to 13.8% (2258/16,359), with MRI remaining the predominant modality (3.7%–7.5%); liver biopsy increased slightly (4.2%–5.8%). Among those without ESLD, any NIT or imaging test rose from 7.3% (2453/33,624) to 11.1% (3740/33,624), with transient elastography remaining most common (5.5%–7.8%); liver biopsy increased from 3.7% to 4.8%. Liver function test capture increased across analytes in both groups, but fasting glucose remained rarely recorded (<1%).

In the MASLD sensitivity cohort, 462,224 patients met the inclusion criteria (Supplementary Figure 3). At baseline, 76,950 (16.6%) had ESLD: 7328 with CC; 67,658 with DC; 965 with HCC; and 999 with LT. The remaining 385,274 had no baseline ESLD; of these, 57,873 (15.0%) progressed to ESLD during follow-up (CC n = 4355 [7.5%]; DC n = 52,459 [90.6%]; HCC n = 695 [1.2%]; LT n = 364 [0.6%]), while 327,401 did not progress.

Testing patterns in the MASLD cohort paralleled the primary MASH analysis, with higher use of NITs, imaging tests, liver biopsy, and liver function tests among patients with baseline ESLD and increased testing from the diagnosis window to follow-up (see Supplementary Table 7, Supplementary Results). Among patients without baseline ESLD, progression patterns in the MASLD cohort were directionally consistent with the MASH cohort (Supplementary Table 8, Supplementary Results). The median time to the first ESLD event was 9.7 months. Among individual outcomes, LT occurred earliest (median 8.1 months; IQR: 3.0–18.3), while HCC occurred latest (median 11.4 months; IQR: 3.5–21.5). All-cause death was observed in 1.9% of patients (n = 6300), with a median time of 18.9 months (IQR: 8.6–30.8).

Broadening to MASLD yielded a similar pattern of predictors of progression as in the primary MASH cohort: higher risk with older age, greater comorbidity burden, hypertension, T2DM, CVD, sleep apnea, and smoking. Relative to MASH, 2 differences were notable as follows: Asian race showed a stronger protective association (aRR: 0.77 [95% CI: 0.73–0.80]), and end-stage renal disease exhibited a stronger positive association with progression (aRR: 1.46 [95% CI: 1.36–1.56]). Overall, the direction and magnitude of most associations were consistent with our primary analysis (Supplementary Table 9).

Patterns in health-care use and costs in the larger MASLD cohort mirrored primary MASH findings. Patients with baseline ESLD had higher adjusted total PPPY costs than those without ESLD, with consistently greater spending across components (Supplementary Table 10). Among patients without baseline ESLD, those who progressed had markedly higher adjusted total costs and higher HCRU across all settings than nonprogressors (Supplementary Table 11).

In a sensitivity analysis that reclassified patients who experienced an ESLD event within 30 days of MASH diagnosis (n = 563) as having baseline ESLD, the proportion with progression to ESLD decreased from 15.9% to 14.5%. Among those without ESLD at baseline, the median time to first ESLD progression event increased from 10.6 months (IQR: 3.8–19.7) in the primary analysis to 12.1 months (IQR: 5.8–21.0) after reclassifying these early events. Predictors of ESLD progression were consistent with primary analysis findings (Supplementary Table 12). HCRU and cost outcomes showed a similar pattern to the primary findings, with even higher utilization and costs among patients with ESLD at baseline and among those who progressed after reclassifying early ESLD events as baseline ESLD (Supplementary Tables 13 and 14).

Lastly, in a post-hoc sensitivity analysis of the MASH cohort with additional adjustment for baseline statin use, the association between dyslipidemia and risk of progression was unchanged, with a similar risk estimate to the primary analysis (aRR: 0.81 [0.76–0.86]).

Discussion

In this large, real-world cohort of 49,983 US adults with MASH, one-third had evidence of ESLD at baseline, and among those without ESLD, 16% progressed during follow-up. DC was the most common first progression event, and the median time to first ESLD event was short (∼11 months). This rapid progression in observed diagnoses is in contrast to prior natural history studies that have reported overall progression from MASH to cirrhosis or ESLD of approximately 7%–12% over follow-up periods of 7–20 years.[19–22] The observed substantially higher prevalence and timing of progression status in our study likely reflects the capture of clinical recognition, rather than true disease onset and reflects real-world risk following identification. Thus, these findings highlight a rapid trajectory from MASH identification to clinically meaningful liver disease and echo prior evidence linking advancing fibrosis with stepwise increases in decompensation and mortality.[23] From a health-system perspective, patients with baseline ESLD and those who progressed incurred substantially higher utilization and costs across care settings, consistent with prior US real-world studies showing sharp cost increases at advanced fibrosis, decompensation, and HCC.[24] Importantly, among patients without ESLD at baseline, those who

progressed reached mean annual costs comparable to baseline ESLD patients within a short interval after diagnosis. These data underscore the clinical and economic urgency of earlier identification and risk-directed management.

Use of NITs and imaging tests around diagnosis was modest overall, though higher with baseline ESLD; during follow-up, NIT and imaging test utilization increased in both strata. Given current guidance that recommends NIT utilization when MASH is suspected and subsequent routine first-line fibrosis risk stratification (eg, FIB-4) in primary and specialty care,[15,25,26] these data suggest a gap between guidance and practice and an opportunity to standardize early risk assessment, particularly for high-risk patients. Furthermore, liver biopsy was infrequently used in this cohort, with only 4% of patients undergoing the procedure for diagnosis. This is consistent with prior reports that biopsy is performed in only about 10% of patients before a MASH diagnosis, despite its role as the reference standard for confirming liver disease.[15,27,28] Other economic analyses demonstrate that sequential use of NITs in primary care is an effective way to rationalize secondary care referrals and is associated with substantial cost savings of over 40%.[29] Further evidence suggests that when primary care physicians identify patients at risk of advanced fibrosis via NITs and refer them to specialists, diagnosis of the condition increases fourfold.[30] When promptly followed by intensive lifestyle interventions or pharmacological treatments, research supports this as a cost-effective approach to managing MASH.[30]

Several baseline characteristics were independently associated with higher progression risk in our study, including older age, hypertension, T2DM, CVD, sleep apnea, smoking and thyroid disease. These findings are consistent with the broader MASLD literature. A multicenter, biopsy-based study ($n = 511$) reported that hypertension and T2DM were independently associated with advanced fibrosis ($\geq$F3).[31] An individual-participant meta-analysis linked obstructive sleep apnea severity with hepatic steatosis.[32] Cohort and meta-analytic data associate smoking with greater fibrosis severity and worse outcomes.[33] CVD reflects a higher-risk cardiometabolic phenotype that tracks with advanced liver disease.[34] Thyroid dysfunction has been associated with MASLD and higher odds of advanced fibrosis.[35] Lastly, the inverse association with dyslipidemia is unlikely to reflect true biological protection, as noted in prior observational analyses.[36,37] Although the post-hoc sensitivity analysis that additionally adjusted for baseline statin use yielded a risk estimate similar to the primary analysis, residual confounding is likely and may explain the observed protective association.

Mortality diverged sharply by progression status as follows: cumulative mortality separated early and continued to widen, and despite a smaller at-risk population, more deaths accrued among progressors than nonprogressors. Preventing progression, and identifying patients approaching decompensation, is therefore critical to reduce death and downstream health-care use.[16] These observations align with a US multicenter prospective cohort showing stepwise increases in decompensation and mortality with advancing fibrosis and ~7-fold higher all-cause mortality after any decompensation.[38]

Strengths of this study include leveraging a large, real-world cohort of 49,983 adults with clinically identified MASH drawn from a well-validated database linking EHR and claims, enabling comprehensive capture of diagnoses, procedures, laboratory tests, imaging/NITs, health-care utilization, and costs. The use of linked EHR claims further supports laboratory and baseline risk profiles observed in prior studies and provides actionable insight into patient stratification, gaps between clinical guidance and real-world practice, and opportunities to refine management strategies. Unlike prior research,[10,17] we used a 24-month preindex lookback to better identify prevalent cirrhosis and ESLD, reducing misclassification at index and yielding a more stable baseline for risk stratification and cost estimation. In addition, we excluded patients exposed to resmetirom (Rezdiffra) to characterize natural history and economic burden in the pretreatment era in the United States. We applied a rigorous cohort design with an extended baseline/exclusion horizon to minimize misclassification of prevalent ESLD and to define a cleaner at-risk population, alongside a standardized first-event ordering for progression end points (CC, DC, HCC, and LT). Importantly, the patterns of progression, HCRU, and costs were robust when the cohort was expanded to MASLD, with similar gradients by baseline ESLD and by progression status.

Limitations

This study has several limitations. First, although we used a large, well-validated EHR–claims database, data accuracy depends on the quality and completeness of source documentation and coding. Misclassification is possible for exposures, comorbidities, and outcomes; BMI and some laboratory measures were sparsely populated, which may impair risk adjustment and subgroup definitions. In particular, very early ESLD "progression" events (eg, within 1 month of MASH diagnosis) likely reflect a lag between true disease onset and when an ICD code is first recorded, or patients whose first presentation is with decompensation, rather than rapid progression from incident MASH. To address this, we conducted a sensitivity analysis reclassifying early ESLD events as baseline ESLD, which produced results similar to the primary analysis, but some residual misclassification of undiagnosed or uncoded MASH is still likely. Second, based on a source population of more than 9 million individuals in Optum Market Clarity, we would expect more than 49,983 MASH diagnoses assuming a prevalence of 14% in middle-aged Americans with a similar median age to this cohort.[6] It is likely that ICD-10-coded MASH diagnoses are under ascertained and preferentially recorded in patients with more advanced or clinically apparent disease. Consequently, the study cohort likely represents a higher severity population rather than the full

spectrum of MASH. However, results of our sensitivity analysis using a broader MASLD population (n = 462,224; closely aligned with expected patient numbers) yielded similar results, which supports the robustness of our findings to alternative cohort definitions and partially mitigates concerns about underascertainment of ICD-coded MASH. As a result, observed event rates and time-to-event should be interpreted with caution as they may overstate progression rates in the general MASH population. Instead, these findings highlight a real-world signal for the need of earlier identification and intervention in routine care.

Additionally, as with any claims-based analysis, misclassification due to coding errors, potentially including upcoding to meet coverage criteria, may have biased estimates, particularly for ESLD component outcomes, by inflating apparent disease severity and progression. Next, differential surveillance (eg, imaging, NIT uptake) may have led to ascertainment bias, with higher test intensity increasing the likelihood of detecting progression. Although liver MRI was more frequently observed among patients with baseline ESLD than among those without, these findings should be interpreted with caution when assessing NIT and imaging utilization. In this population, MRI use likely reflects a mix of cross-sectional imaging for HCC surveillance, evaluation of portal hypertension, and MRI-PDFF for MASH assessment, which cannot be reliably differentiated due to the absence of specific CPT codes.[39] Therefore, NIT and imaging utilization were not separated, and as a result NIT usage without imaging in the baseline ESLD cohort is likely lower. Consistent with this interpretation, MRE was infrequently used in the overall cohort. Generalizability may also be limited, as the study primarily reflects commercially insured US patients and may not represent uninsured, publicly insured, or non-US populations or other care settings. Finally, the study period overlaps with the COVID-19 pandemic, during which disruptions in healthcare delivery and shifts in coding and utilization patterns could confound observed utilization and progression rates.

Conclusion

In this large US real-world MASH cohort, progression risk was higher with older age and presence of cardiometabolic comorbidities. MASH was associated with substantial healthcare costs, which increased markedly among patients with cirrhosis at baseline and among those who progressed during follow-up. The relatively low uptake of NITs and imaging tests at diagnosis highlights a practical opportunity to strengthen clinical care pathways, particularly in high-risk patients, to identify advanced disease earlier and expedite referral.

From a health-system perspective, prioritizing targeted fibrosis assessment and longitudinal monitoring in patients with obesity, T2DM, CVD, and related metabolic disorders may improve risk stratification and enable earlier intervention. Standardizing early testing, optimizing risk-factor control, and timely initiation of effective pharmacologic therapy may reduce decompensation, hospitalizations, and downstream costs. Future work should evaluate the cost-effectiveness of these strategies, assess whether patients receiving MASH-indicated therapies experience slower progression and better clinical outcomes, and examine subgroups to guide implementation at scale.

Supplementary Materials

Material associated with this article can be found, in the online version, at https://doi:10.1016/j.gastha.2026.101031.

References

1. Younossi ZM, Golabi P, Paik JM, et al. The global epidemiology of nonalcoholic fatty liver disease (NAFLD) and nonalcoholic steatohepatitis (NASH): a systematic review. Hepatology 2023;77(4):1335–1347.
2. Younossi ZM, Kalligeros M, Henry L. Epidemiology of metabolic dysfunction-associated steatotic liver disease. Clin Mol Hepatol 2025;31(Suppl):S32–S50.
3. Zhang W, Lu W, Jiao Y, et al. Identifying disease progression biomarkers in metabolic associated steatotic liver disease (MASLD) through weighted gene co-expression network analysis and machine learning. J Transl Med 2025;23(1):472.
4. Wang S, Friedman SL. Found in translation-fibrosis in metabolic dysfunction-associated steatohepatitis (MASH). Sci Transl Med 2023;15(716):adi0759.
5. Lazarus JV, Brennan PN, Mark HE, et al. A call for doubling the diagnostic rate of at-risk metabolic dysfunction-associated steatohepatitis. Lancet Reg Health Eur 2025;54:101320.
6. Harrison SA, Gawrieh S, Roberts K, et al. Prospective evaluation of the prevalence of non-alcoholic fatty liver disease and steatohepatitis in a large middle-aged US cohort. J Hepatology 2021;75(2):284–291.
7. Younossi ZM, Zelber-Sagi S, Lazarus JV, et al. Global consensus recommendations for metabolic dysfunction-associated steatotic liver disease and steatohepatitis. Gastroenterology 2025;169:1017–1032.e2.
8. Luthra R, Sheth A. Understanding MASH: an examination of progression and clinical outcomes by disease severity in the TARGET-NASH database. Adv Ther 2025; 42(2):1165–1195.
9. Pericàs JM, Anstee QM, Augustin S, et al. A roadmap for clinical trials in MASH-related compensated cirrhosis. Nat Rev Gastroenterol Hepatol 2024;21(11):809–823.
10. Charlton M, Tonnu-Mihara I, Teng CC, et al. The clinical and economic burdens of metabolic dysfunction-associated steatohepatitis. J Med Econ 2024;27(1):919–930.
11. Witkowski M, Moreno SI, Fernandes J, et al. The economic burden of non-alcoholic steatohepatitis: a systematic review. Pharmacoeconomics 2022;40(8):751–776.
12. Younossi ZM, Mangla KK, Chandramouli AS, et al. Estimating the economic impact of comorbidities in patients with MASH and defining high-cost burden in patients with noncirrhotic MASH. Hepatol Commun 2024;8(8):e0488.
13. (FDA) USFDA. FDA approves first treatment for patients with liver scarring due to fatty liver disease. 2024.

https://www.fda.gov/news-events/press-announcements/fda-approves-first-treatment-patients-liver-scarring-due-fatty-liver-disease. Accessed March 27, 2025.
14. Optum Inc. Optum for business - market clarity data. 2025. https://business.optum.com/en/data-analytics/life-sciences/real-world-data/market-clarity-data.html. Accessed March 27, 2025.
15. Rinella ME, Neuschwander-Tetri BA, Siddiqui MS, et al. AASLD Practice Guidance on the clinical assessment and management of nonalcoholic fatty liver disease. Hepatology 2023;77(5):1797–1835.
16. Dulai PS, Singh S, Patel J, et al. Increased risk of mortality by fibrosis stage in nonalcoholic fatty liver disease: systematic review and meta-analysis. Hepatology 2017;65(5):1557–1565.
17. Fishman JC, Qian C, Kim Y, et al. Cost burden of cirrhosis and liver disease progression in metabolic dysfunction-associated steatohepatitis: a US cohort study. J Manag Care Spec Pharm 2024;30(9):929–941.
18. Benchimol EI, Smeeth L, Guttmann A, et al. The REporting of studies Conducted using Observational Routinely-collected health Data (RECORD) statement. PLoS Med 2015;12(10):e1001885.
19. Angulo P, Kleiner DE, Dam-Larsen S, et al. Liver fibrosis, but no other histologic features, is associated with long-term outcomes of patients with nonalcoholic fatty liver disease. Gastroenterology 2015;149(2):389–397.e310.
20. Hagström H, Nasr P, Ekstedt M, et al. Fibrosis stage but not NASH predicts mortality and time to development of severe liver disease in biopsy-proven NAFLD. J Hepatol 2017;67(6):1265–1273.
21. Nasr P, Ignatova S, Kechagias S, et al. Natural history of nonalcoholic fatty liver disease: a prospective follow-up study with serial biopsies. Hepatol Commun 2018;2(2):199–210.
22. Younes R, Caviglia GP, Govaere O, et al. Long-term outcomes and predictive ability of non-invasive scoring systems in patients with non-alcoholic fatty liver disease. J Hepatol 2021;75(4):786–794.
23. World Health Organization. In: Guidelines for the prevention, diagnosis, care and treatment for people with chronic hepatitis B infection. Geneva: World Health Organization, 2024. https://www.who.int/publications/i/item/9789240090903. Accessed March 27, 2025.
24. Gordon SC, Kachru N, Parker E, et al. Health care use and costs among patients with nonalcoholic steatohepatitis with advanced fibrosis using the fibrosis-4 score. Hepatol Commun 2020;4(7):998–1011.
25. Kim MN, Han JW, An J, et al. KASL clinical practice guidelines for noninvasive tests to assess liver fibrosis in chronic liver disease. Clin Mol Hepatol 2024;30(Suppl):S5–S105.
26. Wattacheril JJ, Abdelmalek MF, Lim JK, et al. AGA clinical practice update on the role of noninvasive biomarkers in the evaluation and management of nonalcoholic fatty liver disease: expert review. Gastroenterology 2023;165(4):1080–1088.
27. Kim Y, Rydqvist P, Ramezani T, et al. Metabolic dysfunction-associated steatohepatitis diagnosis and management in Germany: insights from an expert consensus panel. Liver Int 2025;45(8):e70225.
28. Gbadamosi SO, Evans KA, Brady BL, et al. Noninvasive tests and diagnostic pathways to MASH diagnosis in the United States: a retrospective observational study. J Med Econ 2025;28(1):314–322.
29. Crossan C, Majumdar A, Srivastava A, et al. Referral pathways for patients with NAFLD based on non-invasive fibrosis tests: diagnostic accuracy and cost analysis. Liver Int 2019;39(11):2052–2060.
30. Lazarus JV, Agirre-Garrido L, Díaz LA, et al. Cost-effectiveness of MASH diagnosis and management approaches among those with type 2 diabetes. JAMA Netw Open 2025;8(11):e2542750.
31. Inukai Y, Ito T, Yokoyama S, et al. Type 2 diabetes and hypertension as risk factors for advanced fibrosis in biopsy proven metabolic dysfunction-associated steatotic liver disease. J Dig Dis 2024;25(11–12):685–693.
32. Jullian-Desayes I, Trzepizur W, Boursier J, et al. Obstructive sleep apnea, chronic obstructive pulmonary disease and NAFLD: an individual participant data meta-analysis. Sleep Med 2021;77:357–364.
33. Zein CO, Unalp A, Colvin R, et al. Smoking and severity of hepatic fibrosis in nonalcoholic fatty liver disease. J Hepatol 2011;54(4):753–759.
34. Driessen S, Francque SM, Anker SD, et al. Metabolic dysfunction-associated steatotic liver disease and the heart. Hepatology 2025;82(2):487–503.
35. Mantovani A, Csermely A, Bilson J, et al. Association between primary hypothyroidism and metabolic dysfunction-associated steatotic liver disease: an updated meta-analysis. Gut 2024;73(9):1554–1561.
36. Kezer CA, Schmidt KA, Shah VH. Statin the course: navigating unchartered territory in cirrhosis. Hepatol Commun 2024;8(6):e0456.
37. Singh S, Singh PP, Singh AG, et al. Statins are associated with a reduced risk of hepatocellular cancer: a systematic review and meta-analysis. Gastroenterology 2013;144(2):323–332.
38. Sanyal AJ, Van Natta Mark L, Clark J, et al. Prospective study of outcomes in adults with nonalcoholic fatty liver disease. N Engl J Med 2021;385(17):1559–1569.
39. European Association for the Study of the Liver (EASL); , European Association for the Study of Diabetes (EASD); European Association for the Study of Obesity (EASO). EASL-EASD-EASO clinical practice guidelines on the management of metabolic dysfunction-associated steatotic liver disease (MASLD). J Hepatology 2024;81(3):492–542.

Received February 23, 2026. Accepted June 4, 2026.

Correspondence:
Address correspondence to: Romina Fakhraei, PhD, Broadstreet HEOR, 177 W 7th Ave, Vancouver, British Columbia, Canada. e-mail: rfakhraei@broadstreetheor.com.

Authors' Contributions:
Yestle Kim, Romina Fakhraei, Karissa Johnston, John C. O'Donnell, and Amreen Dinani: Helped to conceive the study design and analytical approach. Melissa Bather: Performed data analysis under the supervision of Reem Mustafa. Yestle Kim, Romina Fakhraei, Romina Fakhraei, Melissa Bather, and Karissa Johnston: Drafted the manuscript. All of the authors provided intellectual contributions to the study conception and design, contributed to data interpretation, revised the manuscript for important intellectual content, and approved the final version to be published and agreed to be accountable for all aspects of the work.

Conflicts of Interest:
These authors disclose the following: Romina Fakhraei, Andrew R. Kennedy, Reem Mustafa, Melissa Bather, and Karissa Johnston are employees of Broadstreet HEOR, which received funds from Madrigal Pharmaceuticals for

this work. Yestle Kimand and John C. O'Donnell are employees of Madrigal Pharmaceuticals. Amreen Dinani has received funds from Madrigal Pharmaceuticals, Novo Nordisk, Petauri Kinect, and the National Institutes of Health. The remaining authors disclose no conflicts.

Funding:
This study was funded by Madrigal Pharmaceuticals Inc. Medical writing/editorial support was provided by Romina Fakhraei, Andrew R. Kennedy, Reem Mustafa, and Karissa Johnston from Broadstreet HEOR and funded by the study sponsor.

Ethical Statement:
This study was exempt from institutional review board review because it used Optum data comprising deidentified health information, consistent with the Health Insurance Portability and Accountability Act Privacy Rule.

Data Transparency Statement:
The data that supports the findings of this study are from Optum's deidentified Market Clarity Data available through Optum. Restrictions apply to the availability of these data, which were used under license for this study. An active third-party agreement between the authors and Optum is required for sharing of the patient-level data.

Reporting Guidelines:
RECORD.

Gastro Hep Advances, Volume 5

Supplemental information

Progression of Metabolic Dysfunction–Associated Steatohepatitis in US Adults Using Linked Records and Claims

Yestle Kim, Romina Fakhraei, John C. O'Donnell, Karissa Johnston, Melissa Bather, Reem Mustafa, Andrew R. Kennedy, and Amreen Dinani

SUPPLEMENTARY MATERIALS

Supplementary Figure 1. Study design diagram

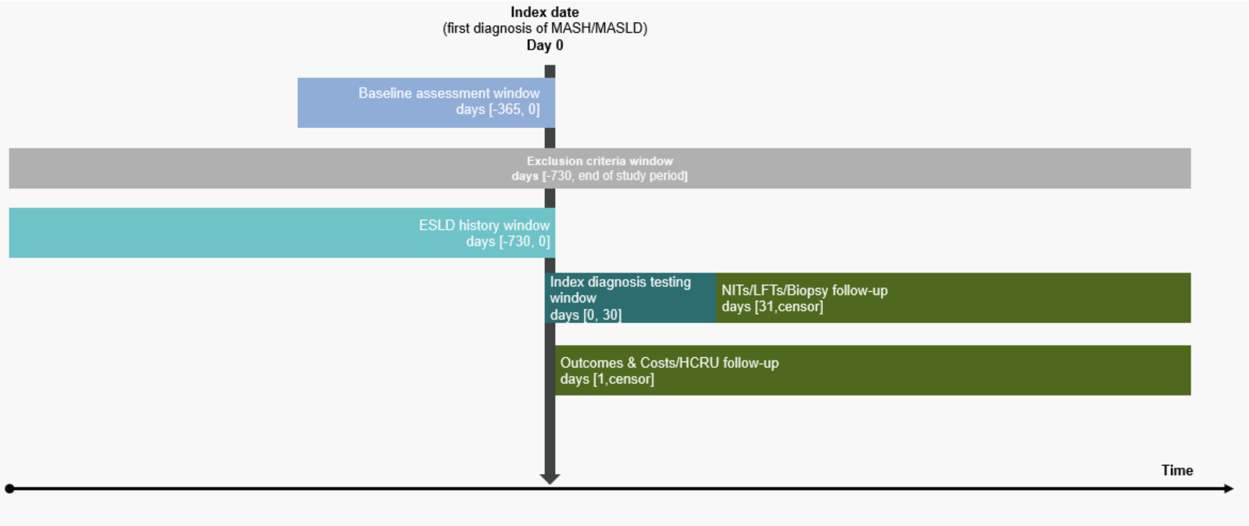

Abbreviations: HCRU, healthcare resource use; LFT, liver function test; MASH, metabolic dysfunction-associated steatohepatitis; MASLD, metabolic dysfunction-associated steatotic liver disease; NIT, non-invasive test.

Supplementary Figure 2a. Mean annual unadjusted per-person per-year healthcare costs by baseline ESLD and progression status

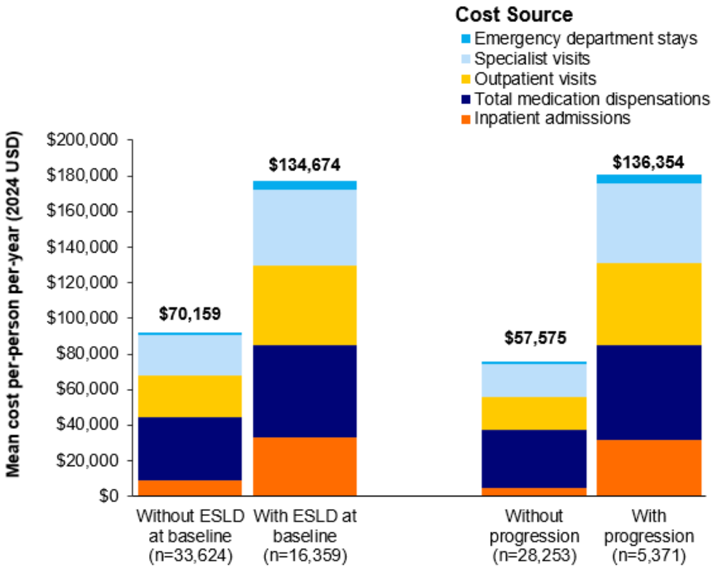

Abbreviations: ESLD, end stage liver disease; USD, United States dollar.

Supplementary Figure 2b. Unadjusted healthcare resource use by baseline ESLD and progression status

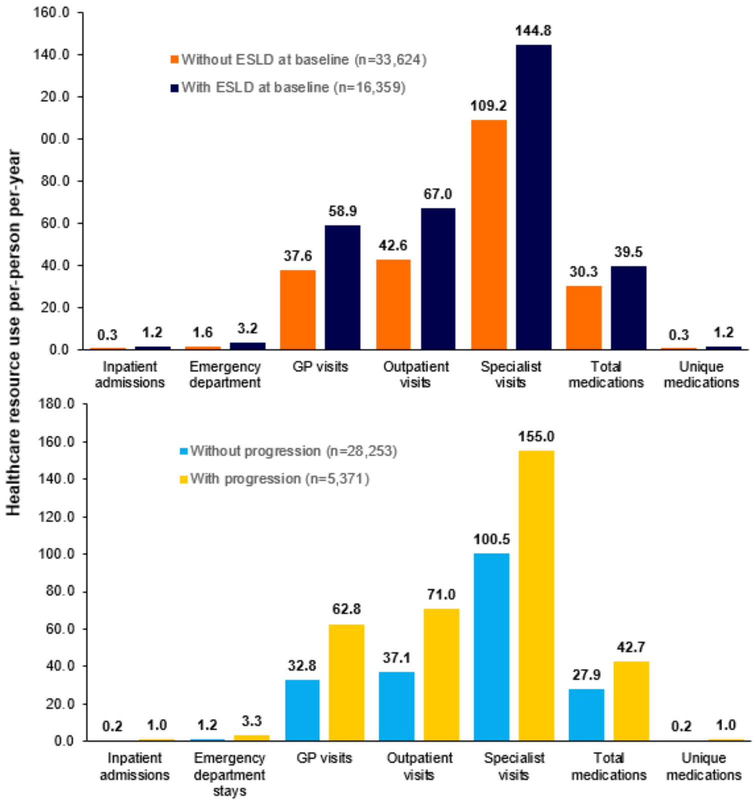

Abbreviations: ESLD, end stage liver disease; GP, general practitioner.

Supplementary Figure 3. Study flow diagram for sensitivity cohort creation (MASH and MASLD)

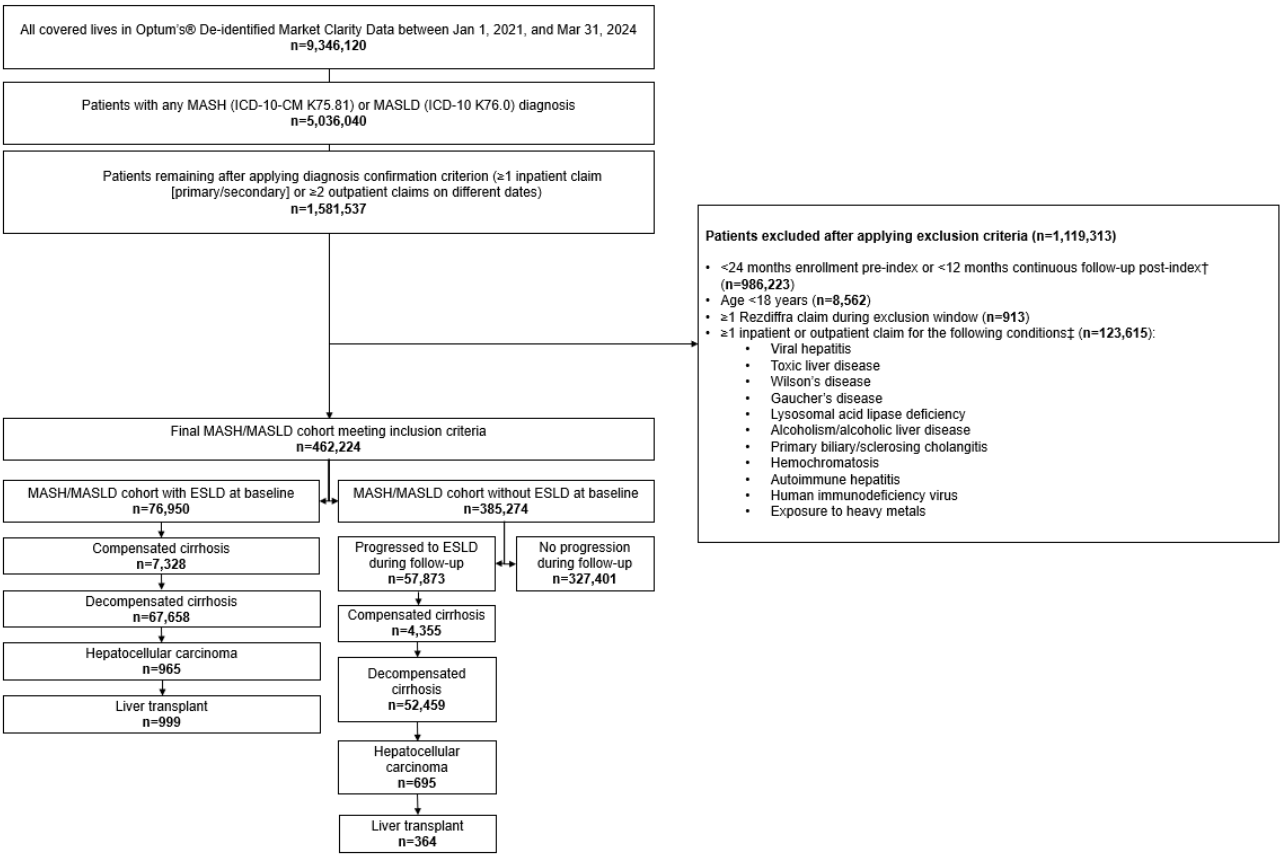

Abbreviations: ESLD, end stage liver disease; ICD-10-CM, International Classification of Diseases 10th revision Clinical Modification; MASH, metabolic dysfunction-associated steatohepatitis; MASLD, metabolic dysfunction-associated steatotic liver disease; T2DM, type 2 diabetes mellitus.
†This criteria is applied to all patients unless the patient had died during this time; ‡Diagnostic codes specified in Supplement Table 1.

Supplementary Table 1. Diagnostic codes for conditions that were excluded from study cohort

Exclusion criteria	Code type	Codes
Viral hepatitis[1]	ICD-10-CM	K75.3, B16.0, B16.1, B16.2, B16.9, B17.0, B18.0, B18.1, B19.10, B19.11, Z22.51, B17.10, B17.11, B18.2, B19.20, B19.21, Z22.52, B00.81, B15.0, B15.9, B17.2, B17.8, B17.9, B18.8, B18.9, B19.0, B19.9, B25.1, B26.81, B94.2, O98.411, O98.412, O98.413, O98.419, O98.42, O98.43, Z22.50, Z22.59
	HCPCS	G8459, G8461, G8463
Toxic liver disease[1]	ICD-10-CM	K71.0, K71.10, K71.11, K71.2, K71.3, K71.4, K71.50, K71.51, K71.6, K71.7, K71.8, K71.9
Wilson's disease[1]	ICD-10-CM	E83.01
Gaucher's disease[1]	ICD-10-CM	E75.22
Lysosomal acid lipase deficiency[1]	ICD-10-CM	E75.5, E75.6
Alcoholism / alcoholic liver disease[1]	ICD-10-CM	E24.4, F10.10, F10.120, F10.121, F10.129, F10.14, F10.150, F10.151, F10.159, F10.180, F10.181, F10.182, F10.188, F10.19, F10.20, F10.21, F10.220, F10.221, F10.229, F10.230, F10.231, F10.232, F10.239, F10.24, F10.250, F10.251, F10.259, F10.26, F10.27, F10.280, F10.281, F10.282, F10.288, F10.29, F10.920, F10.921, F10.929, F10.94, F10.950, F10.951, F10.959, F10.96, F10.97, F10.980, F10.981, F10.982, F10.988, F10.99, G31.2, G62.1, G72.1, I42.6, K29.20, K29.21, K70.0, K70.10, K70.11, K70.2, K70.3, K70.30, K70.31, K70.40, K70.41<br>K70.9, K85.2, K86.0, O35.4XX0, O35.4XX1, O35.4XX2, O35.4XX3, O35.4XX4, O35.4XX5, O35.4XX9, O99.310, O99.311, O99.312, O99.313, O99.314, O99.315
Primary biliary/sclerosing cholangitis[1]	ICD-10-CM	K74.3, K74.4, K74.5, K83.0
Hemochromatosis[1]	ICD-10-CM	E83.118, E83.119
Autoimmune hepatitis[2]	ICD-10-CM	K73.2, K75.4
Human immunodeficiency virus[3]	ICD-10-CM	B20.X, B21.X, B22.X, B23.X, B24, R75, Z21
Exposure to heavy metals[4]	ICD-10-CM	T56.X

Abbreviations: HCPCS, Healthcare Common Procedure Coding System; ICD-10-CM, International Classification of Diseases 10th revision Clinical Modification.

Supplementary Table 2. Codes and definitions used to define study variables

Study variable	Code type(s)	Code(s) or definitions
**End stage liver disease events**		
Compensated cirrhosis (CC)	ICD-10-CM	K74.60, K74.6, K74.4, K74.5, K74.69
Decompensated cirrhosis (DC)	CPT	49083, 49082, 43460, 43244, 43243, 43205, 43227, 43204, 49427, 49425, 37182, 43400, 49084, 78291, 49080, 49081, 49426, 37183, 43401, 43255, 37145, 37160, 37140, 37181, 37180
	HCPCS	C1040
	ICD-10-CM Diagnosis	K72.01, K72.00, K76.2, K72.11, K72.10, K22.8, I85.0, I85.00, I85.01, I85.10, I86.4, I98.20, I98.3, K92.2, K92.0, K72.91, K72.90, K76.81, K76.82, K76.6, K76.7, R17, R18.8, K92.1, J90, I85.11, K65.2, G93.40, G93.41, G93.49, K67, K65.9
	ICD-10-CM Procedures	0D9W00Z, 0D9W30Z, 0D9W40Z, 0D9W0ZZ, 0D9W0ZX, 0D9W3ZZ, 0D9W3ZX, 0D9W4ZZ, 0D9W4ZX, 0DL57DZ, 0DL58DZ
Liver transplant (LT)	CPT	47135, 47136
	DRG	005, 006, 405, 406, 407
	HCPCS	S2053
	ICD-10-CM Diagnosis	T86.40, T86.41, T86.42, T86.43, T86.49, Z48.23, Z94.4
	ICD-10-CM Procedures	0FY00Z0, 0FY00Z1, 0FY00Z2
Hepatic cell carcinoma (HCC)	ICD-10-CM Diagnosis	C22.0, C22.1, C22.8, C24.0
**Fibrosis staging tests**		
Liver biopsy[5]	CPT	37200, 47100, 47001, 47562, 47379, 47000
**Non-invasive tests (NITs)**		
Transient elastography[6]	CPT	91200
Abdominal ultrasound[6]	CPT	76700, 76705
Computed tomography[6]	CPT	74150, 74160, 74170, 74174, 74175, 74176, 74177, 74178
Magnetic resonance imaging[6]	CPT	74181, 74182, 74183, 74185
Magnetic resonance elastography[7†]	CPT	76391
LiverMultiScan[8]	CPT	0648T,0649T
ELF	CPT	81517
Fibrosure/Fibrotest	CPT	81596
**Laboratory-based indices**		
Fibrosis-4 (FIB-4)	Computed using laboratory values	$FIB4 = \frac{Age\ (years) \times AST\ level\ (U/L)}{Platelet\ count\ (10^9/L) \times \sqrt{ALT\ level\ (U/L)}}$
Aspartate aminotransferase-to-platelet ratio index (APRI)	Computed using laboratory values	$APRI = \frac{AST\ level\ (U/L)/AST\ (Upper\ Limit\ of\ Normal\ \ddagger)(U/L)}{Platelet\ count\ (10^9/L)} \times 100$
**Care settings**		
Emergency department[9]	Revenue code	0450, 0451, 0452, 0456, 0459, 0981
Observation unit[10-12]	Revenue code	0762
**Metabolic risk factors**		
Metabolic syndrome (MetS)[13,14]	ICD-10-CM	E88.810
	Laboratory values	Fasting glucose (≥5.6 mmol/L)<br>Triglycerides (≥1.7 mmol/L)

Study variable	Code type(s)	Code(s) or definitions
		HDL-c (<1.29 mmol/L in females; <1.03 mmol/L in males)<br>Systolic blood pressure (≥130 mmHg)<br>Diastolic blood pressure (≥85 mmHg)
	Anthropometric values	Waist circumference (> ethnicity specific values)<br>BMI (≥30kg/m^2)
Hypertension[15-17]	ICD-10-CM	I10, I11, I12, I13, I15
	Laboratory values	Systolic blood pressure (≥140 mmHg)<br>Diastolic blood pressure (≥90 mmHg)
Dyslipidemia[18-20]	ICD-10-CM	E78.0x, E78.1, E78.2, E78.3, E78.4x, E78.5, E78.6, E78.70, E78.89, E78.9, E88.2
	Laboratory values	LDL-c (>3.4 mmol/L)<br>HDL-c (<1.0 mmol/L)<br>Total cholesterol (>5.2 mmol/L)<br>Triglycerides (>1.7 mmol/L)
Obesity*[21,22]	ICD-10-CM	Z68.3x, Z68.4x, E66.x, E65
	Anthropometric values	BMI (≥30kg/m^2)<br>Waist circumference (> ethnicity specific values)
Type 2 diabetes[23,24]	ICD-10-CM	E11.x
	Laboratory values	HbA1c (≥48 mmol/mol)<br>Fasting plasma glucose (≥7.0 mmol/L)<br>2-hour plasma glucose on OGTT (≥11.1 mmol/L)
**Other comorbidities**		
Type 1 diabetes mellitus[23]	ICD-10-CM	E10, E10.3x (excluding E**.34X and E**.35x)
Anemia[25]	ICD-10-CM	D50.x, D53.9, D64.9
Cardiovascular disease[26]		
Rheumatic heart disease	ICD-10-CM	I01-I01.9, I02.0, I05-I09.9
Ischemic heart disease	ICD-10-CM	I20-I21.6, I21.9-I25.9, Z82.4-Z82.49
Cerebrovascular disease	ICD-10-CM	G45-G46.8, I60-I64, I64.1, I65-I69.998, Z82.3
Ischemic stroke	ICD-10-CM	G45-G46.8, I63-I63.9, I65-I66.9, I67.2-I67.848, I69.3-I69.4
Hemorrhagic stroke	ICD-10-CM	I60-I62.9, I67.0-I67.1, I69.0- I69.298
Hypertensive heart disease	ICD-10-CM	I11-I11.2, I11.9
Cardiomyopathy and myocarditis	ICD-10-CM	B33.2-B33.20, B33.22-B33.24, D86.85, I40-I41.8, I42-I43.8, I51.4-I51.6
Myocarditis	ICD-10-CM	B33.2-B33.20, B33.22-B33.24, D86.85, I40-I41.8, I51.4-I51.6
Alcoholic cardiomyopathy	ICD-10-CM	I42.6
Other cardiomyopathy	ICD-10-CM	I42.0-I42.5, I42.7-I43.8
Atrial fibrillation and flutter	ICD-10-CM	I48-I48.92
Peripheral artery disease	ICD-10-CM	I70.2-I70.92, I73-I73.9
Endocarditis	ICD-10-CM	B33.21, I33-I33.9, I38-I38.0, I39-I39.9
Other cardiovascular and circulatory diseases	ICD-10-CM	I30-I32.8, I34-I37.9, I51-I51.3, I51.7-I52.8, I72-I72.9, I77-I83.93, I86-I89.0, I89.9, I95.0-I95.1, I98, I98.8-I99.9, K75.1
Bariatric surgery[27]	ICD-10-PCS	0DB64Z3, 0D16479, 0D1647A, 0D1647B, 0D1647L, 0D164J9, 0D164JA, 0D164JB, 0D164JL, 0D164K9, 0D164KA, 0D164KB, 0D164KL, 0D164Z9, 0D164ZA, 0D164ZB, 0D164ZL, 0D16879, 0D1687A, 0D1687B, 0D1687L, 0D168J9, 0D168JA, 0D168JB, 0D168JL, 0D168K9, 0D168K9, 0D168KA, 0D168KB, 0D168KL, 0D168Z9, 0D168ZA, 0D168ZB, 0D168ZL

Study variable	Code type(s)	Code(s) or definitions
Fatigues/insomnia[§]	ICD-10-CM	R53.0, R53.1, R53.8x, F51, F51.0, F51.1, F51.11, F51.13, F51.19, F51.2, G47.0
Peptic ulcer disease, dyspepsia, GERD, esophagitis	ICD-10-CM	K20, K21, K25, K26, K27, K28, K30
	HCPCS	PBL, PBU, DR016, DR024
Sleep apnea[28]	ICD-10-CM	G47.3, G47.30, G47.30o, G47.31, G47.310, G47.33, G47.34, G47.37, G47.38o, G47.39, R06.81
Smoking, current or past[†29]	ICD-10-AM	F17.1, F17.2, Z72.0, Z86.43, F17.2, Z72.0
Thyroid disease[30]	ICD-10-CM	E02, E03, E05, E05.0, E06, E06.3,
Thyroid cancer[30]	ICD-10-CM	C73
Vitamin D deficiency[31]	ICD-10-CM	E55
PCOS[32]	ICD-10-CM	E28.2
Menopause[33¶]	ICD-10-CM	N95
	NDC (drug classes/generic drug names)[1]	Natural and semisynthetic estrogens
Renal impairment[34]	ICD-10-CM	N18, N18.1, N18.2, N18.3, N18.4, N18.5, N17, Z99.2, N49.2
	HCPCS codes	PBL, PBU, DR016, DR024
	NDC (drug classes/generic drug names)[1]	Dialysis solutions, electrolyte depleters, electrolyte maintenance, erythropoiesis-stimulating agents, kidney stone agents, mineral replacement, multivitamin preparations
End-stage-renal disease[35]	ICD-10-CM	I12.0, I13.11, N18.5, N18.6, Z49.31, Z49.32, Z91.151, Z91.158, Z99.2

Abbreviations: ALT, alanine aminotransferase; AST, aspartate aminotransferase; BMI, body mass index; CC, compensated cirrhosis; CKD, chronic kidney disease; CPT, Current Procedural Terminology; DC, decompensated cirrhosis; DRG, diagnosis-related group; ELF, enhanced liver fibrosis; HbA1c, hemoglobin A1c; HCC, hepatocellular carcinoma; HCPCS, Healthcare Common Procedure Coding System; HDL-c, high-density lipoprotein cholesterol; ICD-10-CM, International Classification of Diseases 10th Revision Clinical Modification; ICD-10-PCS, International Classification of Diseases 10th Revision Procedure Coding System; LDL-c, low-density lipoprotein cholesterol; LT, liver transplant; LSM, liver stiffness measure; NDC, National Drug Code; OGTT, oral glucose tolerance test; PCOS, polycystic ovary syndrome.

[†]Captures magnetic resonance imaging derived proton density fat fraction (MRIDPPFF), since MRIDPPFF does not have unique CPT codes[6,36]; [‡]A fixed value of 40 U/L was used for the Upper Limit of Normal; [§]Note that there will likely be limitations to capturing these with claims data, where positive predictive value is high but negative predictive value is low[22]; [¶]this algorithm used International Classification of Diseases, 9th Revision, Clinical Modification and these codes were converted to ICD-10-CM to reflect the current nomenclature.

Supplementary Table 3. Unadjusted and adjusted risk ratios (RRs) for predictors of disease progression (composite outcome) among patients with MASH

Predictor	Unadjusted RR (95% CI)	Adjusted RR (95% CI)†
**Age**		
Age at index	1.02 (1.02, 1.03)	1.02 (1.02, 1.02)
**Gender**		
Male (reference)	-	-
Female	1.13 (1.07, 1.18)	1.02 (0.97, 1.08)
**Race**		
Caucasian (reference)	-	-
African American	0.86 (0.77, 0.97)	0.83 (0.74, 0.94)
Asian	0.75 (0.66, 0.86)	0.90 (0.79, 1.03)
Other/Unknown	0.82 (0.77, 0.89)	0.96 (0.89, 1.08)
**Ethnicity**		
Not Hispanic (reference)	-	-
Hispanic	0.87 (0.81, 0.94)	0.96 (0.88, 1.03)
Unknown	0.87 (0.81, 0.94)	0.96 (0.88, 1.04)
**Region**		
Northeast (reference)	-	-
Midwest	1.17 (1.10, 1.25)	1.07 (1.00, 1.14)
South	1.14 (1.07, 1.22)	1.07 (1.00, 1.14)
West	0.99 (0.91, 1.08)	0.94 (0.86, 1.02)
Other/Unknown	1.18 (1.05, 1.33)	1.15 (1.03, 1.29)
**Comorbidities**		
Weighted Elixhauser index	1.03 (1.03, 1.04)	1.02 (1.02, 1.03)
Hypertension	1.50 (1.42, 1.58)	1.18 (1.12, 1.26)
Dyslipidemia	1.13 (1.07, 1.19)	0.80 (0.76, 0.85)
Obesity	1.07 (1.02, 1.13)	0.97 (0.92, 1.02)
T2DM	1.51 (1.44, 1.58)	1.25 (1.19, 1.32)
CVD	1.70 (1.63, 1.79)	1.18 (1.12, 1.24)
Sleep apnea	1.30 (1.24, 1.37)	1.13 (1.07, 1.19)
Smoking status (current or past)	1.51 (1.43, 1.60)	1.23 (1.16, 1.30)
Thyroid disease	1.27 (1.21, 1.34)	1.11 (1.05, 1.17)
Vitamin D deficiency	1.08 (1.03, 1.14)	1.03 (0.98, 1.08)
End stage renal disease	2.21 (1.69, 2.88)	1.21 (0.94, 1.56)

Abbreviations: CI, confidence interval; CVD, cardiovascular disease; N/A, not applicable; RR, risk ratio; T2DM, type 2 diabetes mellitus.
†Risk ratios adjusted for age, gender, race, ethnicity, region, Elixhauser index, hypertension, dyslipidemia, obesity, T2DM, smoking, CVD, sleep apnea, thyroid disease, vitamin D deficiency, and end-stage renal disease.

Supplementary Table 4. Healthcare resource utilization and costs during follow-up for MASH cohort (n=49,983), stratified by baseline ESLD

Category	With ESLD at baseline (n=16,359)				Without ESLD at baseline (n=33,624)			
	n (%)	Mean (SD)	Median (IQR)	Adjusted Mean (CI)†	n (%)	Mean (SD)	Median (IQR)	Adjusted Mean (CI)†
**Healthcare resource utilization (PPPY)**								
Inpatient admissions	4,924 (30.1)	1.2 (3.2)	0 (0, 1)	0.6 (0.5, 0.7)	2,754 (8.2)	0.3 (1.5)	0 (0, 0)	0.3 (0.3, 0.3)
ED	8,804 (53.8)	3.2 (6.5)	1 (0, 4)	2.2 (2.0, 2.3)	9,951 (29.6)	1.6 (4.3)	0 (0, 2)	1.5 (1.4, 1.6)
Outpatient visits	16,220 (99.2)	67.0 (72.8)	47 (23, 87)	54.8 (52.9, 56.9)	32,990 (98.1)	42.6 (48.7)	28 (14, 54)	45.0 (43.4, 46.6)
GP visits	16,114 (98.5)	58.9 (66.5)	40 (19, 76)	5.3 (4.9, 5.7)	32,532 (96.8)	37.6 (45.1)	24 (11, 48)	4.3 (4.0, 4.7)
Specialist visits	15,325 (93.7)	144.8 (154.5)	102 (44, 193)	47.5 (45.6, 49.3)	31,314 (93.1)	109.2 (123.2)	74 (32, 143)	38.8 (37.4, 40.3)
Medication dispensations (total)	15,325 (93.7)	39.5 (31.9)	33 (18, 54)	93.8 (90.1, 97.6)	31,314 (93.1)	30.3 (25.4)	25 (13, 41)	88.2 (84.9, 91.7)
Medication dispensations (unique)	4,924 (30.1)	1.2 (3.2)	0 (0, 1)	32.2 (31.2, 33.3)	2,754 (8.2)	0.3 (1.5)	0 (0, 0)	29.5 (28.6, 30.4)
**Healthcare costs (PPPY, 2024 USD)**								
Inpatient admissions	6,359 (38.9)	33,362 (83,640)	0 (0, 28,632)	11,001 (10,164, 11,929)	5,208 (15.5)	8,929 (40,854)	0 (0, 0)	6,497 (6,043, 6,999)
ED stays	10,714 (65.5)	4,989 (11,030)	1,468 (0, 5,663)	1,155 (1,093, 1,215)	15,275 (45.4)	1,947 (5,344)	0 (0, 1868)	843 (798, 891)
Outpatient visits	16,226 (99.2)	44,542 (70,696)	22,839 (8,839, 52,560)	8,156 (7,703, 8,629)	33,224 (98.8)	23,399 (43,871)	10,537 (4,024, 25,815)	6,710 (6,378, 7,092)
GP visits	10,816 (66.1)	2,051 (5,804)	428 (0, 2,007)	208 (181, 237)	19,704 (58.6)	1,179 (4,436)	183 (0, 1,142)	179 (156, 204)
Specialist visits	16,151 (98.7)	42,491 (69,575)	21,156 (7,871, 49,531)	8,033 (7,609, 8508)	32,996 (98.1)	22,220 (43,012)	9,649 (3,497, 24,451)	6,622 (6,294, 6,999)

Category	With ESLD at baseline (n=16,359)				Without ESLD at baseline (n=33,624)			
	n (%)	Mean (SD)	Median (IQR)	Adjusted Mean (CI)†	n (%)	Mean (SD)	Median (IQR)	Adjusted Mean (CI)†
Medication dispensations (total)‡	15,455 (94.5)	51,781 (166,379)	15,285 (2,457, 50,379)	21,014 (20,529, 21,472)	31,996 (95.2)	35,885 (103,226)	7,047 (1,098, 32,148)	13,289 (13,150, 13,433)
Total healthcare cost	16,337 (99.9)	134,674 (222,532)	71,527 (27,483, 164,022)	27,372 (25,975, 28,554)	33,579 (99.9)	70,159 (132,823)	30,237 (10,108, 75,334)	21,489 (20,483, 22,401)

Abbreviations: CI, confidence interval; ED, emergency department; ESLD, end stage liver disease; IQR, interquartile range; GP, general practitioner; PPPY, per-person-per-year; SD, standard deviation; USD, United States Dollar.
†Multivariable models were adjusted for adjusted for age, gender, race, ethnicity, region, Elixhauser index, hypertension, dyslipidemia, obesity, T2DM, smoking, CVD, sleep apnea, thyroid disease, vitamin D deficiency, end-stage renal disease and prior cost and healthcare resource utilization. ‡For medication dispensations, the full multivariable cost model did not converge because of overdispersion; therefore, adjusted means for this row are based on a model including only the exposure (i.e., history of ESLD at baseline) and not the full set of covariates.

Supplementary Table 5. Healthcare resource utilization and costs during follow-up for MASH cohort without baseline ESLD (n=33,624), stratified by progression status

Category	Progression ESLD (n=5,371)				No progression ESLD (n=28,253)			
	n (%)	Mean (SD)	Median (IQR)	Adjusted Mean (CI)†	n (%)	Mean (SD)	Median (IQR)	Adjusted Mean (CI)†
**Healthcare resource utilization (PPPY)**								
Inpatient admissions	729 (13.6)	1.0 (2.9)	0 (0, 1)	0.7 (0.6, 0.9)	2,025 (7.2)	0.2 (0.9)	0 (0, 0)	0.2 (0.1, 0.2)
ED	2,083 (38.8)	3.3 (6.9)	1 (0, 4)	2.3 (2.0, 2.7)	7,868 (27.8)	1.2 (3.5)	0 (0, 1)	1.1 (1.0, 1.3)
Outpatient visits	5,312 (98.9)	71 (71.4)	51 (27, 90)	57.8 (53.7, 62.3)	27,678 (98.0)	37.1 (40.8)	25 (12, 48)	40.4 (37.6, 43.4)
GP visits	5,266 (98.0)	62.8 (65.8)	44 (23, 81)	4.6 (3.9, 5.4)	27,266 (96.5)	32.8 (38.1)	22 (10, 42)	3.2 (2.7, 3.8)
Specialist visits	5,062 (94.2)	155 (156.6)	110 (55, 208)	51.8 (47.9, 56.1)	26,252 (92.9)	100.5 (113.8)	68 (30, 132)	35.6 (33.0, 38.5)
Medication dispensations (total)	5,062 (94.2)	42.7 (32.1)	36 (21, 58)	89.1 (81.9, 97.0)	26,252 (92.9)	27.9 (23.2)	23 (12, 38)	78.5 (72.3, 85.3)
Medication dispensations (unique)	729 (13.6)	1.0 (2.9)	0 (0, 1)	29.8 (27.9, 31.9)	2,025 (7.2)	0.2 (0.9)	0 (0, 0)	25.6 (24.0, 27.4)
**Healthcare costs (PPPY, 2024 USD)**								
Inpatient admissions	1,983 (36.9)	31,948 (82,020)	0 (0, 24,442)	18,506 (16,912, 20,263)	3,225 (11.4)	4,552 (24,244)	0 (0, 0)	3,308 (2,986, 3,632)
ED stays	3,613 (67.3)	4,917 (9,810)	1,594 (0, 5,738)	1,647 (1,561, 1,740)	11,662 (41.3)	1,382 (3,702)	0 (0, 1,354)	618 (580, 659)
Outpatient visits	5,349 (99.6)	46,414 (74,980)	24,600 (11,034, 53,481)	11,326 (10,384, 12,210)	27,875 (98.7)	19,024 (33,199)	8,911 (3,507, 21,301)	5,826 (5,348, 6,308)
GP visits	3,614 (67.3)	2,168 (7,187)	468 (0, 2,126)	262 (210, 423)	16,090 (56.9)	991 (3,657)	152 (0, 1,010)	172 (137, 273)
Specialist visits	5,329 (99.2)	44,245 (73,699)	22,967 (9,811, 50,664)	11,194 (10,267, 12,068)	27,667 (97.9)	18,033 (32,551)	8,130 (2,954, 20,042)	5,729 (5,273, 6,198)

Category	Progression ESLD (n=5,371)				No progression ESLD (n=28,253)			
	n (%)	Mean (SD)	Median (IQR)	Adjusted Mean (CI)†	n (%)	Mean (SD)	Median (IQR)	Adjusted Mean (CI)†
Medication dispensations (total)‡	5,169 (96.2)	53,075 (126,018)	15,178 (2,770, 51,347)	8,471 (8,458, 8,484)	26,827 (95.0)	32,617 (97,959)	5,896 (952, 28,943)	6,300 (6,300, 6,301)
Total healthcare cost	5,371 (100)	136,354 (191,352)	72,254 (29,287, 163,163)	36,498 (34,444, 38,358)	28,208 (99.8)	57,575 (114,214)	25,587 (8,616, 63,020)	16,305 (15,438, 17,027)

Abbreviations: CI, confidence interval; ESLD, end stage liver disease; IQR, interquartile range; GP, general practitioner; PPPY, per-person-per-year; SD, standard deviation; USD, United States Dollar.

†Multivariable models were adjusted for adjusted for age, gender, race, ethnicity, region, Elixhauser index, hypertension, dyslipidemia, obesity, T2DM, smoking, CVD, sleep apnea, thyroid disease, vitamin D deficiency, end-stage renal disease and prior cost and healthcare resource utilization. ‡For medication dispensations, the full multivariable cost model did not converge because of overdispersion; therefore, adjusted means for this row are based on a model including only the exposure (i.e., ESLD progression) and not the full set of covariates.

Supplementary Table 6. Sensitivity analysis - Use of non-invasive tests, liver function blood tests and liver biopsies with extended index diagnosis window [day -15, day 90]

Test type	Total (n=49,983)	With ESLD at baseline (n=16,359)	Without ESLD at baseline (n=33,624)
	n (%)	n (%)	n (%)
**Non-invasive tests (NITs) and imaging**			
At least one NIT or imaging test (any)	5,998 (12)	2,258 (13.8)	3,740 (11.1)
Transient elastography	3,619 (7.2)	978 (5.9)	2,641 (7.8)
Enhanced Liver Fibrosis (ELF)	0 (0)	0 (0)	0 (0)
Magnetic resonance elastography (MRE)	122 (0.24)	37 (0.2)	85 (0.3)
LiverMultiScan	8 (0.02)	n<5[†]	6 (0.02)
Fibrosure / Fibrotest	481 (1.0)	127 (0.8)	354 (1.1)
Magnetic resonance imaging (MRI)	2,105 (4.2)	1,231 (7.5)	874 (2.6)
**Liver biopsy**	2,560 (5.1)	945 (5.8)	1,615 (4.8)
**Liver function blood tests**			
Albumin and total protein	7,743 (15.5)	2,905 (17.8)	4,838 (14.4)
Bilirubin	7,679 (15.4)	2,864 (17.5)	4,815 (14.3)
Alkaline phosphatase (ALP)	7,515 (15.0)	2,815 (17.2)	4,700 (14.0)
Aspartate transaminase (AST)	7,613 (15.2)	2,841 (17.4)	4,772 (14.2)
Alanine transaminase (ALT)	7,644 (15.3)	2,850 (17.4)	4,794 (14.3)
Gamma-glutamyltransferase (GGT)	4,580 (9.2)	1,781 (10.9)	2,799 (8.3)
L-lactate dehydrogenase (LD)	4,273 (8.6)	1,702 (10.4)	2,571 (7.7)
Platelet count (PLT)	6,455 (12.9)	2,525 (15.4)	3,930 (11.7)
Prothrombin time (PT)	4,993 (10.0)	2,159 (13.2)	2,834 (8.4)
Fasting glucose	213 (0.4)	51 (0.31)	162 (0.5)

Abbreviations: ALP, alkaline phosphatase; ALT, alanine transaminase; AST, aspartate transaminase; CT, computed tomography; ELF, enhanced liver fibrosis; ESLD, end stage liver disease; GGT, gamma-glutamyltransferase; LD, L-lactate dehydrogenase; NIT, non-invasive test; PLT, platelet count; PT, prothrombin time.[†]Per agreement with data provider, results with n<5 have been redacted.

Supplementary Table 7. Sensitivity analysis - Use of non-invasive tests (NITs), liver function blood tests and liver biopsies at MASH/MASLD index diagnosis testing window (day 0,30) and during follow-up, by baseline ESLD status

Test type	Within index diagnosis testing window (day 0, 30)		During follow-up (day 31, end of follow-up)	
	With ESLD at baseline (n=76,950)	Without ESLD at baseline (n=385,274)	With ESLD at baseline (n=76,950)	Without ESLD at baseline (n=385,274)
	n (%)	n (%)	n (%)	n (%)
**Non-invasive tests (NITs) and imaging**				
At least one NIT or imaging test (any)	6,891 (9.0)	26,675 (6.9)	14,157 (18.4)	53,111 (13.8)
Transient elastography	2,574 (3.3)	11,434 (2.9)	5,323 (6.9)	24,920 (6.5)
Enhanced Liver Fibrosis (ELF)	0 (0.0)	0 (0.0)	62 (0.1)	310 (0.1)
Magnetic resonance elastography (MRE)	138 (0.2)	446 (0.1)	527 (0.7)	1,959 (0.5)
LiverMultiScan	n<5[†]	12 (<0.1)	32 (<0.1)	131 (<0.1)
Fibrosure / Fibrotest	415 (0.5)	1,954 (0.5)	866 (1.1)	4,668 (1.2)
Magnetic resonance imaging (MRI)	4,038 (5.2)	13,751 (3.6)	9,112 (11.8)	27,447 (7.1)
**Liver biopsy**	1,815 (2.4)	6,585 (1.7)	3,250 (4.2)	14,313 (3.7)
**Liver function blood tests**				
Albumin and total protein	10,349 (13.4)	42,257 (11.0)	17,218 (22.4)	74,655 (19.4)
Bilirubin	10,543 (13.7)	43,624 (11.3)	17,017 (22.1)	73,650 (19.1)
Alkaline phosphatase (ALP)	9,961 (12.9)	40,785 (10.6)	16,698 (21.7)	72,398 (18.8)
Aspartate transaminase (AST)	10,112 (13.1)	41,699 (10.8)	16,817 (21.9)	73,047 (19.0)
Alanine transaminase (ALT)	10,174 (13.2)	41,985 (10.9)	16,828 (21.9)	73,153 (19.0)
Gamma-glutamyltransferase (GGT)	711 (0.9)	3,178 (0.8)	8,369 (10.9)	34,047 (8.8)
L-lactate dehydrogenase (LD)	877 (1.1)	1,680 (0.4)	8,143 (10.6)	31,786 (8.3)
Platelet count (PLT)	7,680 (10.0)	27,839 (7.2)	15,947 (20.7)	75,753 (19.7)
Prothrombin time (PT)	4,104 (5.3)	7,090 (1.8)	10,302 (13.4)	36,897 (9.6)
Fasting glucose	106 (0.1)	716 (0.2)	547 (0.7)	3,104 (0.8)

Abbreviations: ALP, alkaline phosphatase; ALT, alanine transaminase; AST, aspartate transaminase; CT, computed tomography; ELF, enhanced liver fibrosis; ESLD, end stage liver disease; GGT, gamma-glutamyltransferase; LD, L-lactate dehydrogenase; MRE, magnetic resonance elastography; MRI, magnetic resonance imaging; NIT, non-invasive test; PLT, platelet count; PT, prothrombin time. [†]Per agreement with data provider, results with n<5 have been redacted.

Supplementary Table 8. Sensitivity analysis - Progression events among patients with MASH/MASLD without ESLD at baseline, overall and in high-risk subgroups

Progression category	Definition	Without ESLD at baseline (n=385,274)		≥2 cardiometabolic risk factors (n=132,966)†		≥3 cardiometabolic risk factors (n=243,094)†		Obesity (n=180,935)		T2DM (n=308,780)	
		n (%)	Median (IQR)‡	n (%)	Median (IQR)‡	n (%)	Median (IQR)‡	n (%)	Median (IQR)‡	n (%)	Median (IQR)‡
Compensated Cirrhosis (CC)	CC, no prior DC/HCC/LT	4,355 (0.9)	9.0 (2.8, 18.6)	3,403 (1.0)	9.5 (3.0, 19.2)	2,459 (1.1)	9.5 (3.0, 19.2)	2,769 (1.0)	9.3 (3.0, 18.7)	2,074 (1.4)	9.2 (2.9, 18.8)
Decompensated Cirrhosis (DC)	DC, no prior HCC/LT	52,459 (11.3)	9.7 (2.9, 19.0)	38,758 (11.8)	10.1 (3.1, 19.3)	26,787 (12.2)	10.1 (3.2, 19.3)	32,019 (11.4)	9.6 2.9, 18.9)	19,365 (12.6)	10.4 (3.4, 19.5)
Hepatocellular carcinoma (HCC)	HCC, no prior LT	695 (0.2)	11.4 (3.5, 21.5)	550 (0.2)	12.2 (4.0, 22.3)	401 (0.2)	12.3 (3.8, 23.0)	416 (0.1)	11.3 (3.4, 22.0)	338 (0.2)	12.4 (4.6, 22.7)
Liver Transplant (LT)	LT, regardless of previous state*	364 (0.1)	8.1 (3.0, 18.3)	275 (0.1)	8.7 (3.1, 18.3)	204 (0.1)	8.2 (2.9, 18.2)	232 (0.1)	9.1 (3.4, 18.5)	164 (0.1)	9.3 (3.2, 18.7)
Death	All-cause	7,592 (1.6)	19.1 (8.6, 30.7)	6,300 (1.9)	18.9 (8.6, 30.8)	4,654 (2.1)	19.0 (8.5, 31.0)	4,380 (1.6)	19.1 (8.3, 30.7)	3,771 (2.5)	19.3 (8.9, 31.6)
Composite ESLD progression	First of CC, DC, HCC, LT	57,873 (12.5)	9.7 (2.9, 19.0)	42,986 (13.1)	10.1 (3.1, 19.3)	29,851 (13.6)	10.1 (3.2, 19.4)	35,436 (12.6)	9.6 (3.0, 18.9)	21,941 (14.3)	10.3 (3.4, 19.5)

Abbreviations: DC, decompensated cirrhosis; ESLD, end stage liver disease; HCC, hepatocellular carcinoma; IQR, interquartile range; LT, liver transplant; T2DM, type 2 diabetes mellitus.

†Cardiometabolic risk factors include metabolic syndrome, hypertension, dyslipidemia, T2DM, and obesity; ‡Median time-to-event (months)

Supplementary Table 9. Sensitivity analysis - Unadjusted and adjusted risk ratios (RRs) for predictors of disease progression (composite outcome) among patients with MASH/MASLD

Predictor	Unadjusted RR (95% CI)	Adjusted RR (95% CI)†
**Age**		
Age at index	1.02 (1.02, 1.02)	1.01 (1.01, 1.02)
**Gender**		
Male (reference)	-	-
Female	1.05 (1.04, 1.07)	1.03 (1.01, 1.04)
**Race**		
Caucasian (reference)	-	-
African American	1.00 (0.97, 1.03)	0.94 (0.91, 0.97)
Asian	0.67 (0.64, 0.70)	0.77 (0.73, 0.80)
Other/Unknown	0.82 (0.80, 0.84)	0.94 (0.92, 0.96)
**Ethnicity**		
Not Hispanic (reference)	-	-
Hispanic	0.85 (0.83, 0.87)	0.92 (0.90, 0.94)
Unknown	0.88 (0.86, 0.89)	0.97 (0.95, 1.00)
**Region**		
Northeast (reference)	-	-
Midwest	1.05 (1.03, 1.07)	0.98 (0.96, 1.00)
South	1.06 (1.04, 1.08)	0.99 (0.98, 1.01)
West	0.96 (0.94, 0.99)	0.93 (0.91, 0.95)
Other/Unknown	1.10 (1.06, 1.14)	1.06 (1.02, 1.10)
**Comorbidities**		
Weighted Elixhauser index	1.04 (1.04, 1.04)	1.03 (1.03, 1.03)
Hypertension	1.46 (1.43, 1.48)	1.21 (1.19, 1.23)
Dyslipidemia	1.13 (1.11, 1.15)	0.83 (0.82, 0.85)
Obesity	1.03 (1.02, 1.05)	0.92 (0.91, 0.94)
T2DM	1.40 (1.38, 1.42)	1.18 (1.16, 1.19)
CVD	1.74 (1.71, 1.76)	1.23 (1.21, 1.25)
Sleep apnea	1.25 (1.23, 1.27)	1.10 (1.08, 1.11)
Smoking status (current or past)	1.48 (1.46, 1.50)	1.22 (1.20, 1.24)
Thyroid disease	1.20 (1.18, 1.22)	1.06 (1.04, 1.08)
Vitamin D deficiency	1.03 (1.01, 1.04)	0.98 (0.96, 0.99)
End stage renal disease	2.59 (2.42, 2.79)	1.46 (1.36, 1.56)

Abbreviations: CI, confidence interval; CVD, cardiovascular disease; N/A, not applicable; RR, risk ratio; T2DM, type 2 diabetes mellitus.
†Risk ratios adjusted for age, gender, race, ethnicity, region, Elixhauser index, hypertension, dyslipidemia, obesity, T2DM, smoking, CVD, sleep apnea, thyroid disease, vitamin D deficiency, and end-stage renal disease.

Supplementary Table 10. Sensitivity analysis - Healthcare resource utilization and costs during follow-up for MASH/MASLD cohort (n=462,224), stratified by baseline ESLD

Category	With ESLD at baseline (n=76,950)				Without ESLD at baseline (n=385,274)			
	n (%)	Mean (SD)	Median (IQR)	Adjusted Mean (CI)†	n (%)	Mean (SD)	Median (IQR)	Adjusted Mean (CI)†
**Healthcare resource utilization (PPPY)**								
Inpatient admissions	25,454 (33.1)	1.2 (3.2)	0 (0, 1)	0.6 (0.5, 0.6)	40,308 (10.5)	0.4 (1.5)	0 (0, 0)	0.4 (0.4, 0.4)
Emergency department stays	46,354 (60.2)	3.9 (9.4)	1 (0, 4)	2.2 (2.2, 2.3)	144,661 (37.5)	1.9 (4.7)	1 (0, 2)	1.8 (1.7, 1.8)
Outpatient visits	76,258 (99.1)	65 (73.2)	44 (21, 84)	47.8 (47.1, 48.5)	377,343 (97.9)	41.2 (48)	27 (13, 53)	41.6 (41.0, 42.3)
GP visits	75,740 (98.4)	57.4 (67.4)	38 (17, 74)	4.5 (4.4, 4.6)	371,841 (96.5)	36.2 (44.3)	23 (10, 46)	4.0 (3.9, 4.1)
Specialist visits	72,467 (94.2)	137.8 (156.1)	92 (39, 181)	41.9 (41.2, 42.6)	357,244 (92.7)	99.7 (116.5)	66 (27, 130)	36.3 (35.8, 36.9)
Medication dispensations (total)	72,467 (94.2)	39 (32.7)	32 (17, 53)	84.1 (82.6, 85.5)	357,244 (92.7)	29.2 (25)	23 (12, 40)	81.7 (80.4, 83.1)
Medication dispensations (unique)	25,454 (33.1)	1.2 (3.2)	0 (0, 1)	30.2 (29.8, 30.6)	40,308 (10.5)	0.4 (1.5)	0 (0, 0)	28.7 (28.3, 29.0)
**Healthcare costs (PPPY, 2024 USD)**								
Inpatient admissions	28,586 (37.1)	32,669 (89,634)	0 (0, 24,613)	11,000 (10,164, 11,929)	69,950 (18.2)	10,095 (41,824)	0 (0, 0)	6,497 (6,043, 6,999)
Emergency department stays	45,696 (59.4)	4,179 (10,835)	784 (0, 4,309)	1,155 (1,093, 1,215)	168,344 (43.7)	1,724 (5,152.5)	0 (0, 1,632)	843 (798, 891)
Outpatient visits	71,990 (93.6)	28,006 (65,870)	8,276 (1,347, 28,245)	8,156 (7,703, 8,629)	344,876 (89.5)	14,465 (41,985)	3,295 (331, 13,577)	6,710 (6,378, 7,092)
GP visits	32,968 (42.8)	816 (5,518)	0 (0, 134)	208 (181, 237)	141,799 (36.8)	448 (3,605)	0 (0, 27)	179 (156, 204)
Specialist visits	70,832 (92.0)	27,190 (65,168)	7,705 (1,145, 27,124)	8,034 (7,609, 8,508)	335,369 (87.0)	14,016 (41,542)	3,020 (227, 12,968)	6,622 (6,294, 6,999)

Category	With ESLD at baseline (n=76,950)				Without ESLD at baseline (n=385,274)			
	n (%)	Mean (SD)	Median (IQR)	Adjusted Mean (CI)†	n (%)	Mean (SD)	Median (IQR)	Adjusted Mean (CI)†
Medication dispensations (total)‡	73,076 (95.0)	43,491 (139, 713)	9,943 (1,603, 36,898)	21,014 (20,529, 21,472)	366,276 (95.1)	29,524 (106,752)	4,525 (793, 23,724)	13,289 (13,150, 13,433)
Total healthcare cost	76,334 (99.2)	108,345 (201,249)	44,778 (12,861, 122,077)	27,372 (25,975, 28,554)	382,131 (99.2)	55,807 (133,117)	19,090 (4,422, 55,854)	21,489 (20,483, 22,401)

Abbreviations: CI, confidence interval; ESLD, end stage liver disease; IQR, interquartile range; GP, general practitioner; PPPY, per-person-per-year; SD, standard deviation; USD, United States Dollar.

†Multivariable models were adjusted for adjusted for age, gender, race, ethnicity, region, Elixhauser index, hypertension, dyslipidemia, obesity, T2DM, smoking, CVD, sleep apnea, thyroid disease, vitamin D deficiency, end-stage renal disease and prior cost and healthcare resource utilization. ‡For medication dispensations, the full multivariable cost model did not converge because of overdispersion; therefore, adjusted means for this row are based on a model including only the exposure (i.e., history of ESLD at baseline) and not the full set of covariates.

Supplementary Table 11. Sensitivity analysis - Healthcare resource utilization and costs during follow-up for MASH/MASLD cohort without baseline ESLD (n=385,274), stratified by progression status

Category	Progression ESLD (n=57,873)				No progression ESLD (n=327,401)			
	n (%)	Mean (SD)	Median (IQR)	Adjusted Mean (CI)†	n (%)	Mean (SD)	Median (IQR)	Adjusted Mean (CI)†
**Healthcare resource utilization (PPPY)**								
Inpatient admissions	10,235 (17.7)	1.2 (3.1)	0 (0, 1)	0.9 (0.9, 1.0)	30,073 (9.2)	0.2 (0.9)	0 (0, 0)	0.2 (0.2, 0.3)
Emergency department stays	27,790 (48.0)	4 (7.8)	2 (0, 5)	2.9 (2.8, 3.0)	116,871 (35.7)	1.5 (3.9)	0 (0, 2)	1.4 (1.4, 1.5)
Outpatient visits	57,105 (98.7)	69.5 (69.9)	50 (26, 90)	56.7 (55.4, 57.9)	320,238 (97.8)	36.2 (40.9)	24 (12, 47)	37.6 (36.8, 38.4)
GP visits	56,575 (97.8)	61.6 (64.3)	43 (22, 80)	4.9 (4.7, 5.1)	315,266 (96.3)	31.8 (38)	21 (9, 41)	3.5 (3.4, 3.7)
Specialist visits	54,266 (93.8)	142.8 (153.5)	99 (46, 187)	50.4 (49.2, 51.6)	302,978 (92.5)	92.1 (106.8)	61 (25, 121)	32.8 (32.1, 33.6)
Medication dispensations (total)	54,266 (93.8)	41.6 (32.5)	35 (19, 56)	88.4 (86.2, 90.6)	302,978 (92.5)	27 (22.8)	22 (11, 37)	75.3 (73.5, 77.1)
Medication dispensations (unique)	10,235 (17.7)	1.2 (3.1)	0 (0, 1)	31.5 (30.9, 32.1)	30,073 (9.2)	0.2 (0.9)	0 (0, 0)	26.0 (25.5, 26.5)
**Healthcare costs (PPPY, 2024 USD)**								
Inpatient admissions	24,783 (42.8)	36,703 (86,816)	0 (0, 33,997)	18,507 (16,912, 20,263)	45,167 (13.8)	5,391 (24,061)	0 (0, 0)	3,308 (2,987, 3,633)
Emergency department stays	37,864 (65.4)	4,306 (8,996)	1,355 (0, 5,076)	1,648 (1,561, 1,740)	130,480 (39.9)	1,267 (3,943)	0 (0, 1185)	619 (581, 659)
Outpatient visits	55,411 (95.7)	30,534 (69,462)	10,534 (2,306, 30,919)	11,326 (10,385, 12,210)	289,465 (88.4)	11,624 (34,172)	2,622 (209, 11,149)	5,826 (5,348, 6,309)
GP visits	25,515 (44.1)	829 (5,632)	0 (0, 151)	262 (210, 424)	116,284 (35.5)	381 (3,108)	0 (0, 14)	172 (137, 273)
Specialist visits	54,752 (94.6)	29,705 (68,812)	9,947 (2,081, 29,727)	11,194 (10,267, 12,069)	280,617 (85.7)	11,243 (33,802)	2,377 (132, 10,574)	5,730 (5,273, 6,198)

Category	Progression ESLD (n=57,873)				No progression ESLD (n=327,401)			
	n (%)	Mean (SD)	Median (IQR)	Adjusted Mean (CI)†	n (%)	Mean (SD)	Median (IQR)	Adjusted Mean (CI)†
Medication dispensations (total)‡	55,620 (96.1)	43,979 (149,736)	10,126 (1,853, 37,223)	8,471 (8,458, 8,485)	310,656 (94.9)	26,969 (96,973)	3,858 (699, 21,485)	6,301 (6,301, 6,301)
Total healthcare cost	57,749 (99.8)	115,521 (206,512)	52,190 (16,904, 135,065)	36,498 (34,444, 38,358)	324,382 (99.1)	45,251 (112,126)	15,842 (3,647, 46,239)	16,306 (15,438, 17,027)

Abbreviations: CI, confidence interval; ESLD, end stage liver disease; IQR, interquartile range; GP, general practitioner; PPPY, per-person-per-year; SD, standard deviation; USD, United States Dollars

†Multivariable models were adjusted for adjusted for age, gender, race, ethnicity, region, Elixhauser index, hypertension, dyslipidemia, obesity, T2DM, smoking, CVD, sleep apnea, thyroid disease, vitamin D deficiency, end-stage renal disease and prior cost and healthcare resource utilization. ‡For medication dispensations, the full multivariable cost model did not converge because of overdispersion; therefore, adjusted means for this row are based on a model including only the exposure (i.e., ESLD progression) and not the full set of covariates.

Supplementary Table 12. Sensitivity analysis - Unadjusted and adjusted risk ratios (RRs) for predictors of disease progression (composite outcome) after reclassifying progression events within the first 30 days as baseline ESLD

Predictor	Primary analysis results		Sensitivity analysis results	
	Unadjusted RR (95% CI)	Adjusted RR (95% CI)†	Unadjusted RR (95% CI)	Adjusted RR (95% CI)†
**Age**				
Age at index	1.02 (1.02, 1.03)	1.02 (1.02, 1.02)	1.03 (1.02, 1.03)	1.02 (1.02, 1.02)
**Gender**				
Male (reference)	-	-	-	-
Female	1.13 (1.07, 1.18)	1.02 (0.97, 1.08)	1.15 (1.09, 1.21)	1.04 (0.98, 1.10)
**Race**				
Caucasian (reference)	-	-	-	-
African American	0.86 (0.77, 0.97)	0.83 (0.74, 0.94)	0.87 (0.77, 0.99)	0.84 (0.74, 0.96)
Asian	0.75 (0.66, 0.86)	0.90 (0.79, 1.03)	0.77 (0.67, 0.89)	0.93 (0.81, 1.07)
Other/Unknown	0.82 (0.77, 0.89)	0.96 (0.89, 1.08)	0.82 (0.76, 0.89)	0.97 (0.89, 1.05)
**Ethnicity**				
Not Hispanic (reference)	-	-	-	-
Hispanic	0.87 (0.81, 0.94)	0.96 (0.88, 1.03)	0.87 (0.80, 0.94)	0.96 (0.88, 1.04)
Unknown	0.87 (0.81, 0.94)	0.96 (0.88, 1.04)	0.87 (0.80, 0.94)	0.96 (0.88, 1.05)
**Region**				
Northeast (reference)	-	-	-	-
Midwest	1.17 (1.10, 1.25)	1.07 (1.00, 1.14)	1.15 (1.08, 1.24)	1.06 (0.99, 1.13)
South	1.14 (1.07, 1.22)	1.07 (1.00, 1.14)	1.10 (1.02, 1.18)	1.03 (0.96, 1.11)
West	0.99 (0.91, 1.08)	0.94 (0.86, 1.02)	0.97 (0.89, 1.07)	0.92 (0.83, 1.00)
Other/Unknown	1.18 (1.05, 1.33)	1.15 (1.03, 1.29)	1.18 (1.04, 1.33)	1.15 (1.02, 1.29)
**Comorbidities**				
Weighted Elixhauser index	1.03 (1.03, 1.04)	1.02 (1.02, 1.03)	1.03 (1.03, 1.04)	1.02 (1.02, 1.03)

Predictor	Primary analysis results		Sensitivity analysis results	
	Unadjusted RR (95% CI)	Adjusted RR (95% CI)†	Unadjusted RR (95% CI)	Adjusted RR (95% CI)†
Hypertension	1.50 (1.42, 1.58)	1.18 (1.12, 1.26)	1.53 (1.45, 1.61)	1.19 (1.12, 1.26)
Dyslipidemia	1.13 (1.07, 1.19)	0.80 (0.76, 0.85)	1.16 (1.09, 1.23)	0.81 (0.76, 0.87)
Obesity	1.07 (1.02, 1.13)	0.97 (0.92, 1.02)	1.06 (1.01, 1.12)	0.96 (0.91, 1.02)
T2DM	1.51 (1.44, 1.58)	1.25 (1.19, 1.32)	1.54 (1.46, 1.62)	1.26 (1.20, 1.34)
CVD	1.70 (1.63, 1.79)	1.18 (1.12, 1.24)	1.72 (1.64, 1.81)	1.17 (1.11, 1.24)
Sleep apnea	1.30 (1.24, 1.37)	1.13 (1.07, 1.19)	1.32 (1.25, 1.39)	1.15 (1.09, 1.21)
Smoking status (current or past)	1.51 (1.43, 1.60)	1.23 (1.16, 1.30)	1.52 (1.43, 1.61)	1.23 (1.16, 1.30)
Thyroid disease	1.27 (1.21, 1.34)	1.11 (1.05, 1.17)	1.28 (1.21, 1.36)	1.11 (1.05, 1.17)
Vitamin D deficiency	1.08 (1.03, 1.14)	1.03 (0.98, 1.08)	1.08 (1.03, 1.14)	1.03 (0.97, 1.09)
End stage renal disease	2.21 (1.69, 2.88)	1.21 (0.94, 1.56)	2.32 (1.75, 3.07)	1.27 (0.97, 1.65)

Abbreviations: CI, confidence interval; CVD, cardiovascular disease; N/A, not applicable; RR, risk ratio; T2DM, type 2 diabetes mellitus.

†Risk ratios adjusted for age, gender, race, ethnicity, region, Elixhauser index, hypertension, dyslipidemia, obesity, T2DM, smoking, CVD, sleep apnea, thyroid disease, vitamin D deficiency, and end-stage renal disease.

Supplementary Table 13. Sensitivity analysis of follow-up healthcare resource utilization and costs in the MASH cohort (n=49,983), by baseline ESLD status, after reclassifying progression events within 30 days as baseline ESLD

Category	With ESLD at baseline (n=16,922)				Without ESLD at baseline (n=33,061)			
	n (%)	Mean (SD)	Median (IQR)	Adjusted Mean (CI)†	n (%)	Mean (SD)	Median (IQR)	Adjusted Mean (CI)†
**Healthcare resource utilization (PPPY)**								
Inpatient admissions	5,019 (29.7)	1.2 (3.1)	0 (0, 1)	0.6 (0.5, 0.7)	2,659 (8.0)	0.3 (1.5)	0 (0, 0)	0.3 (0.3, 0.3)
ED	9,063 (53.6)	3.2 (6.5)	1 (0, 4)	2.2 (2.0, 2.4)	9,692 (29.3)	1.6 (4.3)	0 (0, 2)	1.5 (1.4, 1.6)
Outpatient visits	16,778 (99.1)	66.6 (72.4)	47 (23, 86)	55.0 (53.1, 57.1)	32,432 (98.1)	42.3 (48.4)	28 (13, 54)	44.8 43.2, 46.4)
GP visits	16,665 (98.5)	58.6 (66.1)	40 (19, 76)	5.3 (4.9, 5.7)	31,981 (96.7)	37.4 (44.9)	24 (11, 48)	4.3 (4.0, 4.7)
Specialist visits	15,859 (93.7)	144.2 (153.8)	102 (44, 192)	47.6 (45.8, 49.5)	30,780 (93.1)	108.9 (123.1)	74 (32, 143)	38.7 (37.2, 40.2)
Medication dispensations (total)	15,859 (93.7)	39.4 (31.7)	33 (18, 54)	93.9 (90.2, 97.7)	30,780 (93.1)	30.2 (25.3)	24 (13, 41)	88.1 (84.7, 91.6)
Medication dispensations (unique)	5,019 (29.7)	1.2 (3.1)	0 (0, 1)	32.3 (31.3, 33.4)	2,659 (8.0)	0.3 (1.5)	0 (0, 0)	29.4 (28.5, 30.4)
**Healthcare costs (PPPY, 2024 USD)**								
Inpatient admissions	6,534 (38.6)	33,003 (83,015)	0 (0, 27,786)	11,757 (9,682, 13,680)	5,033 (15.2)	8,696 (40,378)	0 (0, 0)	4,459 (3,637, 5,219)
ED stays	11,049 (65.3)	4,936 (10,911)	1,437 (0, 5,601)	1,846 (1,613, 2,104)	14,940 (45.2)	1,923 (5,322)	0 (0, 1,835)	1,044 (905, 1,203)
Outpatient visits	16,785 (99.2)	44,109 (70,094)	22,640 (8,806, 51,909)	16,004 (14,636, 17,386)	32,665 (98.8)	23,260 (43,788)	10,425 (3,978, 25,596)	10,986 (10,045 11,963)
GP visits	11,174 (66.0)	2,054 (5,929)	425 (0, 1,994)	786 (682, 887)	19,346 (58.5)	1,163 (4,320)	180 (0, 1,135)	617 (539, 697)
Specialist visits	16,704 (98.7)	42,055 (68,973)	20,964 (7,827, 48,845)	15,417 (14,067, 16,807)	32,443 (98.1)	22,098 (42,939)	9,533 (3,464, 24,310)	10,517 (9,599, 11,503)

Category	With ESLD at baseline (n=16,922)				Without ESLD at baseline (n=33,061)			
	n (%)	Mean (SD)	Median (IQR)	Adjusted Mean (CI)†	n (%)	Mean (SD)	Median (IQR)	Adjusted Mean (CI)†
Medication dispensations (total)‡	15,993 (94.5)	51,588 (164,961)	15,053 (2,421, 50,085)	25,215 (24,090, 26,445)	31,458 (95.2)	35,712 (102,979)	7,021 (1,094, 32,103)	15,622 (15,154, 16,118)
Total healthcare cost	16,900 (99.9)	133,635 (220,659)	70,659 (27,268, 162,886)	41,118 (37,384, 45,086)	33,016 (99.9)	69,592 (132,366)	29,854 (10,013, 74,751)	27,336 (25,002, 29,964)

Abbreviations: CI, confidence interval; ED, emergency department; ESLD, end stage liver disease; IQR, interquartile range; GP, general practitioner; PPPY, per-person-per-year; SD, standard deviation; USD, United States Dollar.
†Multivariable models were adjusted for adjusted for age, gender, race, ethnicity, region, Elixhauser index, hypertension, dyslipidemia, obesity, T2DM, smoking, CVD, sleep apnea, thyroid disease, vitamin D deficiency, end-stage renal disease and prior cost and healthcare resource utilization. ‡For medication dispensations, the full multivariable cost model did not converge because of overdispersion; therefore, adjusted means for this row are based on a model including only the exposure (i.e., history of ESLD at baseline) and not the full set of covariates.

Supplementary Table 14. Sensitivity analysis of follow-up healthcare resource utilization and costs in the MASH cohort without baseline ESLD, stratified by progression status, after reclassifying progression events within 30 days as baseline ESLD

Category	Progression ESLD (n=4,808)				No progression ESLD (n=28,253)			
	n (%)	Mean (SD)	Median (IQR)	Adjusted Mean (CI)†	n (%)	Mean (SD)	Median (IQR)	Adjusted Mean (CI)†
**Healthcare resource utilization (PPPY)**								
Inpatient admissions	634 (13.2)	1.1 (3.0)	0 (0, 1)	0.7 (0.6, 0.9)	2,025 (7.2)	0.2 (0.9)	0 (0, 0)	0.2 (0.1, 0.2)
ED	1,824 (37.9)	3.4 (7.1)	2 (0, 4)	2.4 (2.0, 2.8)	7,868 (27.8)	1.2 (3.5)	0 (0, 1)	1.1 (1.0, 1.3)
Outpatient visits	4,754 (98.9)	72.8 (72.5)	52 (28, 93)	58.6 (54.4, 63.2)	27,678 (98.0)	37.1 (40.8)	25 (12, 48)	40.7 (37.9, 43.8)
GP visits	4,715 (98.1)	64.4 (66.8)	45 (24, 82)	4.6 (3.9, 5.4)	27,266 (96.5)	32.8 (38.1)	22 (10, 42)	3.2 (2.7, 3.8)
Specialist visits	4,528 (94.2)	158.4 (159.0)	115 (57, 212)	52.6 (48.5, 57.0)	26,252 (92.9)	100.5 (113.8)	68 (30, 132)	36.0 (33.3, 38.9)
Medication dispensations (total)	4,528 (94.2)	43.5 (32.5)	37 (22, 58)	89.2 (81.8, 97.2)	26,252 (92.9)	27.9 (23.2)	23 (12, 38)	78.3 (72.1, 85.2)
Medication dispensations (unique)	634 (13.2)	1.1 (3.0)	0 (0, 1)	29.7 (27.8, 31.8)	2,025 (7.2)	0.2 (0.9)	0 (0, 0)	25.6 (24.0, 27.3)
**Healthcare costs (PPPY, 2024 USD)**								
Inpatient admissions	1,808 (37.6)	33,046 (84,051)	0 (0, 25,678)	11,800 (8,232, 15,493)	3,225 (11.4)	4,552 (24,244)	0 (0, 0)	2,147 (1,410, 2,969)
ED stays	3,278 (68.2)	5,098 (10,123)	1,713 (0, 5,941)	1,791 (1,497, 2,076)	11,662 (41.3)	1,382 (3,702)	0 (0, 1,354)	720 (569, 867)
Outpatient visits	4.790 (99.6)	48,158 (77,353)	25,450 (11,494, 56,071)	16,239 (12,969, 19,899)	27,875 (98.7)	19,024 (33,199)	8,911 (3,507, 21,301)	9,030 (7,257, 10,962)
GP visits	3,256 (67.7)	2,173 (6,970)	487 (0, 2,186)	706 (560, 860)	16,090 (56.9)	991 (3,657)	152 (0, 1,010)	485 (387, 590)
Specialist visits	4,776 (99.3)	45,985 (76,059)	23,960 (10,239, 53,001)	15,662 (12,398, 19,297)	27,667 (97.9)	18,033 (32,551)	8,130 (2,954, 20,042)	8,624 (6,869, 10,558)

Category	Progression ESLD (n=4,808)				No progression ESLD (n=28,253)			
	n (%)	Mean (SD)	Median (IQR)	Adjusted Mean (CI)†	n (%)	Mean (SD)	Median (IQR)	Adjusted Mean (CI)†
Medication dispensations (total)‡	4,631 (96.3)	53,904 (127,078)	16,108 (3,024, 53,165)	21,849 (20,379, 23,305)	26,827 (95.0)	32,617 (97,959)	5,896 (952, 28,943)	14,615 (14,122, 15,083)
Total healthcare cost	4,808 (100.0)	140,205 (194.921)	74,870 (30,607, 167,543)	37,728 (32,235, 43,956)	28,208 (99.8)	57,575 (114,214)	25,587 (8,616, 63,020)	19,782 (16,861, 22,673)

Abbreviations: CI, confidence interval; ESLD, end stage liver disease; IQR, interquartile range; GP, general practitioner; PPPY, per-person-per-year; SD, standard deviation; USD, United States Dollar.

†Multivariable models were adjusted for adjusted for age, gender, race, ethnicity, region, Elixhauser index, hypertension, dyslipidemia, obesity, T2DM, smoking, CVD, sleep apnea, thyroid disease, vitamin D deficiency, end-stage renal disease and prior cost and healthcare resource utilization. ‡For medication dispensations, the full multivariable cost model did not converge because of overdispersion; therefore, adjusted means for this row are based on a model including only the exposure (i.e., ESLD progression) and not the full set of covariates.

SUPPLEMENTARY METHODS

Information on data source:

Optum's de-identified Market Clarity Data (Optum® Market Clarity) is an integrated, multi-source medical claims, pharmacy claims, and electronic health records data set. Optum® Market Clarity links electronic health record data - including lab results, vital signs and measurements, diagnoses, procedures and information derived from unstructured clinical notes using natural language processing - with historical, linked administrative claim data - including pharmacy claims, physician claims, clinical information facility claims and medications prescribed and administered. Optum® Market Clarity is statistically de-identified under the HIPAA Privacy Rule's Expert Determination method and managed according to Optum® customer data use agreements.

The RECORD checklist

	Item number	STROBE items	RECORD items	Location in manuscript
**Title and abstract**				
	1	(a) Indicate the study's design with a commonly used term in the title or the abstract. (b) Provide in the abstract an informative and balanced summary of what was done and what was found.	RECORD 1.1: The type of data used should be specified in the title or abstract. When possible, the name of the databases used should be included. RECORD 1.2: If applicable, the geographic region and time frame within which the study took place should be reported in the title or abstract. RECORD 1.3: If linkage between databases was conducted for the study, this should be clearly stated in the title or abstract.	Title, Abstract
**Introduction**				
Background rationale	2	Explain the scientific background and rationale for the investigation being reported.		Introduction
Objectives	3	State specific objectives, including any prespecified hypotheses.		Introduction
**Methods**				
Study design	4	Present key elements of study design early in the paper.		Methods
Setting	5	Describe the setting, locations, and relevant dates, including periods of recruitment, exposure, follow-up, and data collection.		Methods, Supplementary Methods
Participants	6	(a) Cohort study: Give the eligibility criteria and the sources and methods of selection of participants. Describe	RECORD 6.1: The methods of study population selection (such as codes or algorithms used to	Methods, Supplementary Table 1 and 2

		methods of follow-up. Case-control study: Give the eligibility criteria and the sources and methods of case ascertainment and control selection. Give the rationale for the choice of cases and controls. Cross-sectional study: Give the eligibility criteria and the sources and methods of selection of participants. (b) Cohort study: For matched studies, give matching criteria and number of exposed and unexposed. Case-control study: For matched studies, give matching criteria and the number of controls per case.	identify subjects) should be listed in detail. If this is not possible, an explanation should be provided. RECORD 6.2: Any validation studies of the codes or algorithms used to select the population should be referenced. If validation was conducted for this study and not published elsewhere, detailed methods and results should be provided. RECORD 6.3: If the study involved linkage of databases, consider use of a flow diagram or other graphical display to demonstrate the data linkage process, including the number of individuals with linked data at each stage.	
Variables	7	Clearly define all outcomes, exposures, predictors, potential confounders, and effect modifiers. Give diagnostic criteria, if applicable.	RECORD 7.1: A complete list of codes and algorithms used to classify exposures, outcomes, confounders, and effect modifiers should be provided. If these cannot be reported, an explanation should be provided.	Methods, Supplementary Table 2
Data sources/ measurement	8	For each variable of interest, give sources of data and details of methods of assessment (measurement). Describe comparability of assessment methods if there is more than one group.		Methods
Bias	9	Describe any efforts to address potential sources of bias.		Methods & Results (Sensitivity Analyses)
Study size	10	Explain how the study size was arrived at.		Methods, Figure 1

Quantitative methods	11	Explain how quantitative variables were handled in the analyses. If applicable, describe which groupings were chosen and why.		Methods, Supplementary Table 2
Statistical methods	12	(a) Describe all statistical methods, including those used to control for confounding. (b) Describe any methods used to examine subgroups and interactions. (c) Explain how missing data were addressed. (d) Cohort study: If applicable, explain how loss to follow-up was addressed. Case-control study: If applicable, explain how matching of cases and controls was addressed. Cross-sectional study: If applicable, describe analytical methods taking account of sampling strategy. (e) Describe any sensitivity analyses.		Methods (Statistical Analysis)
Data access and cleaning methods		N/A	RECORD 12.1: Authors should describe the extent to which the investigators had access to the database population used to create the study population. RECORD 12.2: Authors should provide information on the data cleaning methods used in the study.	Methods, Figure 1
Linkage		N/A	RECORD 12.3: State whether the study included person-level, institutional-level, or other data linkage across two or more databases. The methods of linkage and methods of linkage	Methods, Supplementary Methods

			quality evaluation should be provided.	
**Results**				
Participants	13	(a) Report the numbers of individuals at each stage of the study (e.g., numbers potentially eligible, examined for eligibility, confirmed eligible, included in the study, completing follow-up, and analysed). (b) Give reasons for nonparticipation at each stage. (c) Consider use of a flow diagram.	RECORD 13.1: Describe in detail the selection of the persons included in the study (i.e., study population selection), including filtering based on data quality, data availability, and linkage. The selection of included persons can be described in the text and/or by means of the study flow diagram.	Figure 1
Descriptive data	14	(a) Give characteristics of study participants (e.g., demographic, clinical, and social) and information on exposures and potential confounders. (b) Indicate the number of participants with missing data for each variable of interest. (c) Cohort study: summarise follow-up time (e.g., average and total amount).		Results, Table 1
Outcome data	15	Cohort study: Report numbers of outcome events or summary measures over time. Case-control study: Report numbers in each exposure category or summary measures of exposure. Cross-sectional study: Report numbers of outcome events or summary measures.		Results, Figure 1, Table 1
Main results	16	(a) Give unadjusted estimates and, if applicable, confounder-adjusted estimates and their precision (e.g., 95% confidence interval). Make clear which confounders were adjusted for and		Results and Tables

		why they were included. (b) Report category boundaries when continuous variables were categorized. (c) If relevant, consider translating estimates of relative risk into absolute risk for a meaningful time period.		
Other analyses	17	Report other analyses done—e.g., analyses of subgroups and interactions and sensitivity analyses.		Methods & Results
**Discussion**				
Key results	18	Summarise key results with reference to study objectives.		Discussion
Limitations	19	Discuss limitations of the study, taking into account sources of potential bias or imprecision. Discuss both direction and magnitude of any potential bias.	RECORD 19.1: Discuss the implications of using data that were not created or collected to answer the specific research question(s). Include discussion of misclassification bias, unmeasured confounding, missing data, and changing eligibility over time, as they pertain to the study being reported.	Discussion
Interpretation	20	Give a cautious overall interpretation of results considering objectives, limitations, multiplicity of analyses, results from similar studies, and other relevant evidence.		Discussion
Generalisability	21	Discuss the generalisability (external validity) of the study results.		Discussion
**Other information**				
Funding	22	Give the source of funding and the role of the funders for the present study and, if		Title (under "Declarations")

		applicable, for the original study on which the present article is based.		
Accessibility of protocol, raw data, and programming code		N/A	RECORD 22.1: Authors should provide information on how to access any supplemental information such as the study protocol, raw data, or programming code.	Title (under "Availability of data and material")

Abbreviations: N/A, not applicable; RECORD, REporting of studies Conducted using Observational Routinely collected health Data; STROBE, Strengthening the Reporting of Observational Studies in Epidemiology.

SUPPLEMENTARY RESULTS

MASLD Sensitivity Analysis – Additional results

Testing patterns in the MASLD cohort paralleled the primary MASH analysis (**Supplementary Table 7**): patients with baseline ESLD consistently had higher use of NITs, imaging tests, liver biopsy, and liver function tests than those without baseline ESLD. Testing in both groups increased from the diagnosis window (days 0,30) to follow-up (day 31+). Transient elastography and MRI were the most commonly used NIT and imaging tests, while MRE, LiverMultiScan, and FibroSure/Fibrotest were rare. Absolute rates of NITs, imaging tests, and liver biopsy during follow-up were modestly lower in the MASH cohort, but the direction of differences was unchanged.

Among patients without baseline ESLD, progression patterns in the MASLD cohort were directionally consistent with the MASH cohort (**Supplementary Table 8**), with DC as the most common first event and HCC and LT remaining rare. Composite ESLD progression was modestly higher in MASLD than in MASH (12.5% vs 10.7%) and occurred slightly sooner (median ~9–10 months vs ~10–11 months). High-risk subgroups (≥2 or ≥3 cardiometabolic risk factors, obesity, T2DM) showed the same gradient in both cohorts, with the highest progression proportions among those with T2DM.

References

1. Wong RJ, Kachru N, Martinez DJ, Moynihan M, Ozbay AB, Gordon SC. Real-world Comorbidity Burden, Health Care Utilization, and Costs of Nonalcoholic Steatohepatitis Patients With Advanced Liver Diseases. *Journal of clinical gastroenterology.* 2021;55(10):891-902.
2. Grønbæk L, Vilstrup H, Pedersen L, Christensen K, Jepsen P. Family occurrence of autoimmune hepatitis: A Danish nationwide registry-based cohort study. *Journal of hepatology.* 2018;69(4):873-877.
3. Kramer JR, Hartman C, White DL, et al. Validation of HIV-infected cohort identification using automated clinical data in the Department of Veterans Affairs. *HIV medicine.* 2019;20(8):567-570.
4. Han G, Turcotte K, Jivani K, Babul S, Pike I. *Poisonings in British Columbia, 2000 - 2005.* 2009.
5. Tapper EB, Bonafede M, Fishman J, et al. Healthcare resource utilization and costs of care in the United States for patients with non-alcoholic steatohepatitis. *Journal of medical economics.* 2023;26(1):348-356.
6. Allen AM, Van Houten HK, Sangaralingham LR, Talwalkar JA, McCoy RG. Healthcare Cost and Utilization in Nonalcoholic Fatty Liver Disease: Real-World Data From a Large U.S. Claims Database. *Hepatology.* 2018;68(6):2230-2238.
7. Pepin KM, Welle CL, Guglielmo FF, Dillman JR, Venkatesh SK. Magnetic resonance elastography of the liver: everything you need to know to get started. *Abdominal radiology (New York).* 2022;47(1):94-114.
8. Perspectum. LiverMultiScan. 2024; https://www.perspectum.com/our-products/livermultiscan-rollback-content-21apr23. Accessed July 16, 2024.
9. Research Data Assistance Center. How to Identify Hospital Claims for Emergency Room Visits in the Medicare Claims Data. 2015; https://resdac.org/articles/how-identify-hospital-claims-emergency-room-visits-medicare-claims-data.
10. Powell WR, Kaiksow FA, Kind AJH, Sheehy AM. What Is an Observation Stay? Evaluating the Use of Hospital Observation Stays in Medicare. *Journal of the American Geriatrics Society.* 2020;68(7):1568-1572.
11. Research Data Assistance Center. Identifying Observation Stays in the Medicare Fee-For-Service Claims Data. 2020; https://resdac.org/articles/identifying-observation-stays-medicare-fee-service-claims-data.
12. Sheehy AM, Shi F, Kind AJH. Identifying Observation Stays In Medicare Data: Policy Implications of a Definition. *Journal of hospital medicine.* 2019;14(2):96-100.
13. Alberti KGMM, Eckel RH, Grundy SM, et al. Harmonizing the Metabolic Syndrome. *Circulation.* 2009;120(16):1640-1645.
14. Alberti KGMM, Zimmet P, Shaw J. The metabolic syndrome - a new worldwide definition. *The Lancet.* 2005;366(9491):1059-1062.
15. Song S, Lee SE, Oh SK, et al. Demographics, treatment trends, and survival rate in incident pulmonary artery hypertension in Korea: A nationwide study based on the health insurance review and assessment service database. *PloS one.* 2018;13(12):e0209148.
16. Quan H, Khan N, Hemmelgarn BR, et al. Validation of a Case Definition to Define Hypertension Using Administrative Data. *Hypertension.* 2009;54(6):1423-1428.
17. Unger T, Borghi C, Charchar F, et al. 2020 International Society of Hypertension Global Hypertension Practice Guidelines. *Hypertension.* 2020;75(6):1334-1357.
18. Gabriela Dieguez F, Bruce Pyenson F, Tomicki S, Charles Steffens F. Obesity in A Claims-Based Analysis of the Commercially Insured Population: Prevalence, Cost, and the

Influence of Obesity Services and Anti-Obesity Medication Coverage on Health Expenditures - MILLIMAN REPORT. 2021.
19. Mesregah MK, Mgbam P, Fresquez Z, Wang JC, Buser Z. Impact of chronic hyperlipidemia on perioperative complications in patients undergoing lumbar fusion: a propensity score matching analysis. *European Spine Journal.* 2022;31(10):2579-2586.
20. Oake J, Aref-Eshghi E, Godwin M, et al. Using Electronic Medical Record to Identify Patients With Dyslipidemia in Primary Care Settings: International Classification of Disease Code Matters From One Region to a National Database. *Biomedical Informatics Insights.* 2017;9:1178222616685880.
21. Ramasamy A, Laliberté F, Aktavoukian SA, et al. Direct and Indirect Cost of Obesity Among the Privately Insured in the United States: A Focus on the Impact by Type of Industry. *Journal of occupational and environmental medicine.* 2019;61(11):877-886.
22. Suissa K, Schneeweiss S, Lin KJ, Brill G, Kim SC, Patorno E. Validation of obesity-related diagnosis codes in claims data. *Diabetes, obesity & metabolism.* 2021;23(12):2623-2631.
23. Chi GC, Li X, Tartof SY, Slezak JM, Koebnick C, Lawrence JM. Validity of ICD-10-CM codes for determination of diabetes type for persons with youth-onset type 1 and type 2 diabetes. *BMJ open diabetes research & care.* 2019;7(1):e000547.
24. Sacks DB, Arnold M, Bakris GL, et al. Guidelines and Recommendations for Laboratory Analysis in the Diagnosis and Management of Diabetes Mellitus. *Clinical Chemistry.* 2023;69(8):808-868.
25. Military Health System. *ANEMIA; IRON DEFICIENCY.* 2015.
26. Collaboration GBoCD. The Burden of Cardiovascular Diseases Among US States, 1990-2016. *JAMA Cardiology.* 2018;3(5):375-389.
27. Mabeza RM, Mao Y, Maynard K, Lee C, Benharash P, Yetasook A. Bariatric surgery outcomes in geriatric patients: a contemporary, nationwide analysis. *Surgery for Obesity and Related Diseases.* 2022;18(8):1005-1011.
28. Jolley RJ, Liang Z, Peng M, et al. Identifying Cases of Sleep Disorders through International Classification of Diseases (ICD) Codes in Administrative Data. *International journal of population data science.* 2018;3(1):448.
29. Havard A, Jorm LR, Lujic S. Risk adjustment for smoking identified through tobacco use diagnoses in hospital data: a validation study. *PloS one.* 2014;9(4):e95029.
30. Song YS, Kim KS, Kim SK, Cho YW, Choi HG. Screening Leads to Overestimated Associations of Thyroid Dysfunction and Thyroiditis with Thyroid Cancer Risk. *Cancers.* 2021;13(21).
31. Choi R, Cho SE, Lee SG, Lee EH. Recent Information on Vitamin D Deficiency in an Adult Korean Population Visiting Local Clinics and Hospitals. *Nutrients.* 2022;14(9).
32. Yu O, Christ JP, Schulze-Rath R, et al. Incidence, prevalence, and trends in polycystic ovary syndrome diagnosis: a United States population-based study from 2006 to 2019. *American Journal of Obstetrics and Gynecology.* 2023;229(1):39.e31-39.e12.
33. Pham ANQ, Cummings M, Yuksel N, et al. Development and validation of a case definition for problematic menopause in primary care electronic medical records. *BMC Medical Informatics and Decision Making.* 2023;23(1):202.
34. Friberg L, Gasparini A, Carrero JJ. A scheme based on ICD-10 diagnoses and drug prescriptions to stage chronic kidney disease severity in healthcare administrative records. *Clinical kidney journal.* 2018;11(2):254-258.
35. Gibertoni D, Voci C, Iommi M, et al. Developing and validating an algorithm to identify incident chronic dialysis patients using administrative data. *BMC Medical Informatics and Decision Making.* 2020;20(1):185.
36. Pooler BD, Hernando D, Reeder SB. Clinical Implementation of a Focused MRI Protocol for Hepatic Fat and Iron Quantification. *AJR American journal of roentgenology.* 2019;213(1):90-95.
